# Supplementary material for: The yeast mitophagy receptor Atg32 is ubiquitinated and degraded by the proteasome
Source: PLoS One. 2020 Dec 23;15(12):e0241576. doi: 10.1371/journal.pone.0241576 (PMC7757876; doi:10.1371/journal.pone.0241576)
Supplement: S1 Fig — (A) atg32Δ mutant cells grown in a CMS-L medium and expressing Atg32-V5 protein were harvested in a mid-exponential phase of growth. Then, cells were lysed and purified mitochondria prepared as described in Vigie et al. [34] were loaded on a 20–60% sucrose gradient. Fractions were collected and analyzed by immunodetection. Anti-V5 antibody was used to visualize Atg32-V5, and anti-Porin antibody was used to detect mitochondria-containing fractions. (B) Mitophagy was assessed using mitophagy-dependent processing of the Idp1-GFP tool in atg32Δ mutant and atg32Δ–expressing Atg32-V5. Cells were harvested at indicated times. The total protein extracts corresponding to 5 x 10^6^ cells/50 ug proteins per line were separated by 12.5% SDS-PAGE and analyzed by immunodetection using anti-GFP antibody. (PDF) [file pone.0241576.s001.pdf]

# Figure S1

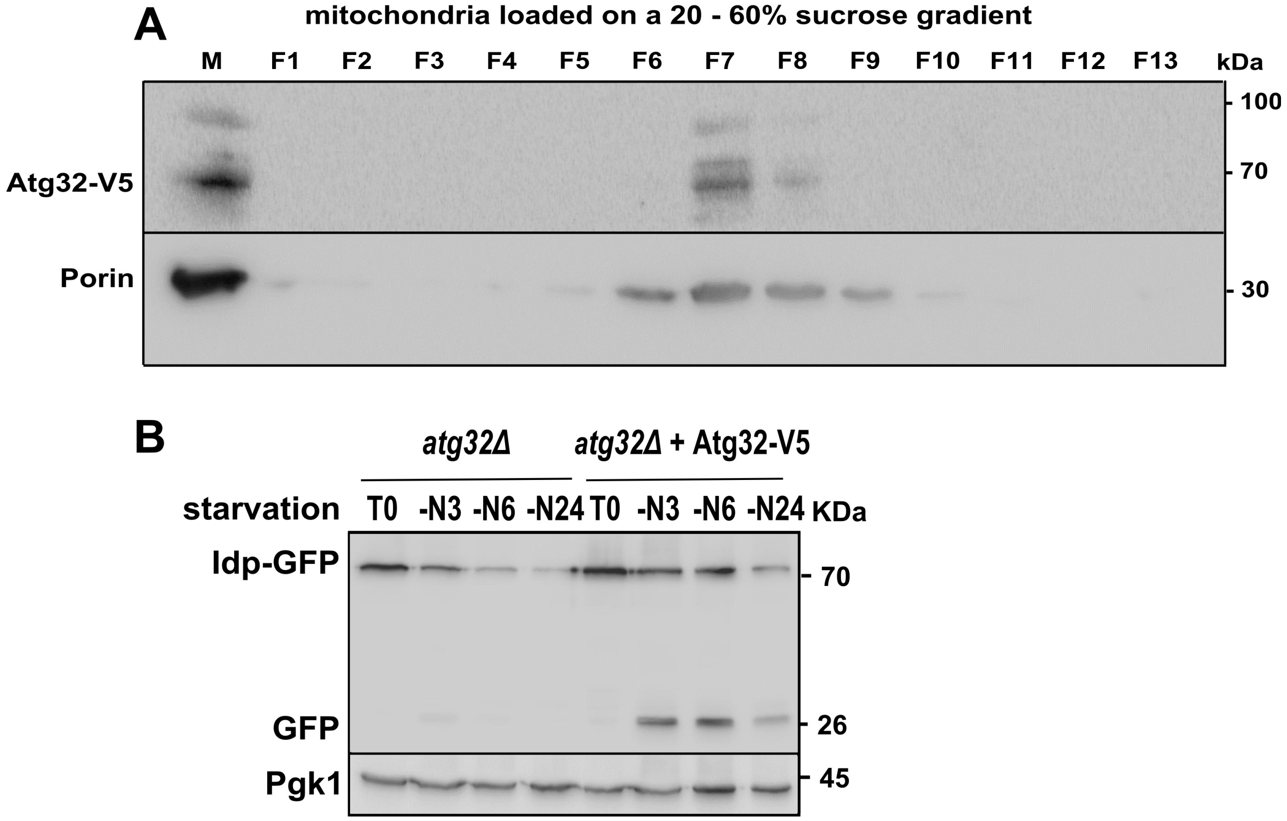


**Figure S1 : Atg32-V5 recombinant protein localizes into mitochondria and restores mitophagy in *atg32Δ* mutant cells upon nitrogen starvation. (A)** *atg32Δ* mutant cells grown in a CMS-L medium and expressing Atg32-V5 protein were harvested in a mid-exponential phase of growth. Then, cells were lysed and purified mitochondria prepared as described in Vigie et al. [34] were loaded on a 20–60% sucrose gradient. Fractions were collected and analyzed by immunodetection. Anti-V5 antibody was used to visualize Atg32-V5, and anti-Porin antibody was used to detect mitochondria-containing fractions. **(B)** Mitophagy was assessed using mitophagy-dependent processing of the Idp1-GFP tool in *atg32Δ* mutant and *atg32Δ*–expressing Atg32-V5. Cells were harvested at indicated times. The total protein extracts corresponding to 5 x $10^6$ cells/50 ug proteins per line were separated by 12.5% SDS-PAGE and analyzed by immunodetection using anti-GFP antibody.
