# Supplementary material for: The yeast mitophagy receptor Atg32 is ubiquitinated and degraded by the proteasome
Source: PLoS One. 2020 Dec 23;15(12):e0241576. doi: 10.1371/journal.pone.0241576 (PMC7757876; doi:10.1371/journal.pone.0241576)
Supplement: S2 Fig — (A) atg32Δ mutant cells grown in a CMS-L medium and expressing Atg32-V5 protein were harvested at indicated times. To inhibit the proteasome, 75 μM MG-132 was added to the cell culture at the beginning of starvation. Total protein extracts were prepared afterwards, and samples were analyzed by western blots. Anti-V5 antibody was used to visualize Atg32-V5 protein; Pgk1 was used as a loading control. Anti-ubiquitin (Ub) was used to detect the level of ubiquitinated proteins. (B) The Atg32-V5/Pgk1 ratio was quantified for all tested conditions. (PDF) [file pone.0241576.s002.pdf]

Figure S2

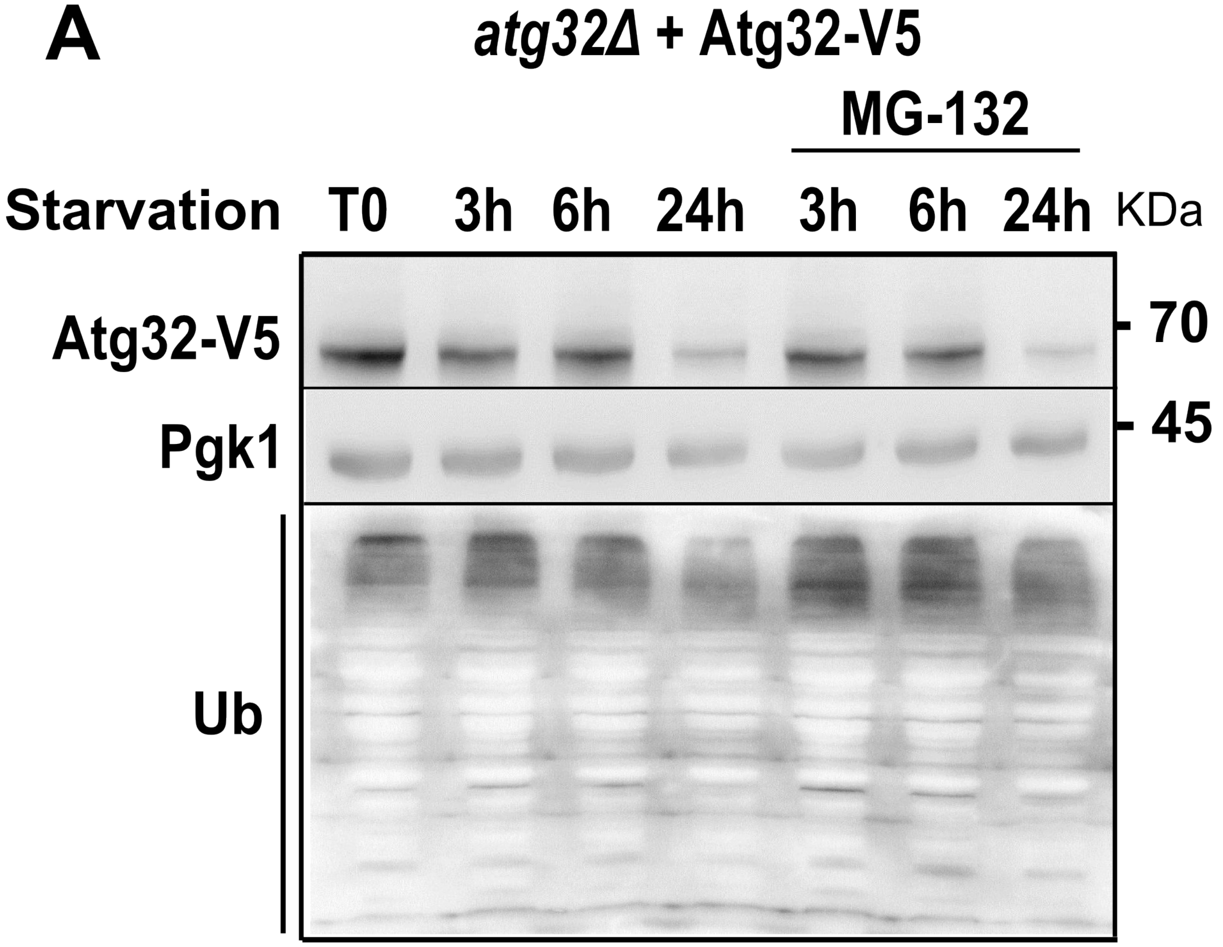
A
atg32Δ + Atg32-V5
MG-132
Starvation T0 3h 6h 24h 3h 6h 24h KDa
Atg32-V5
Pgk1
Ub
70
45

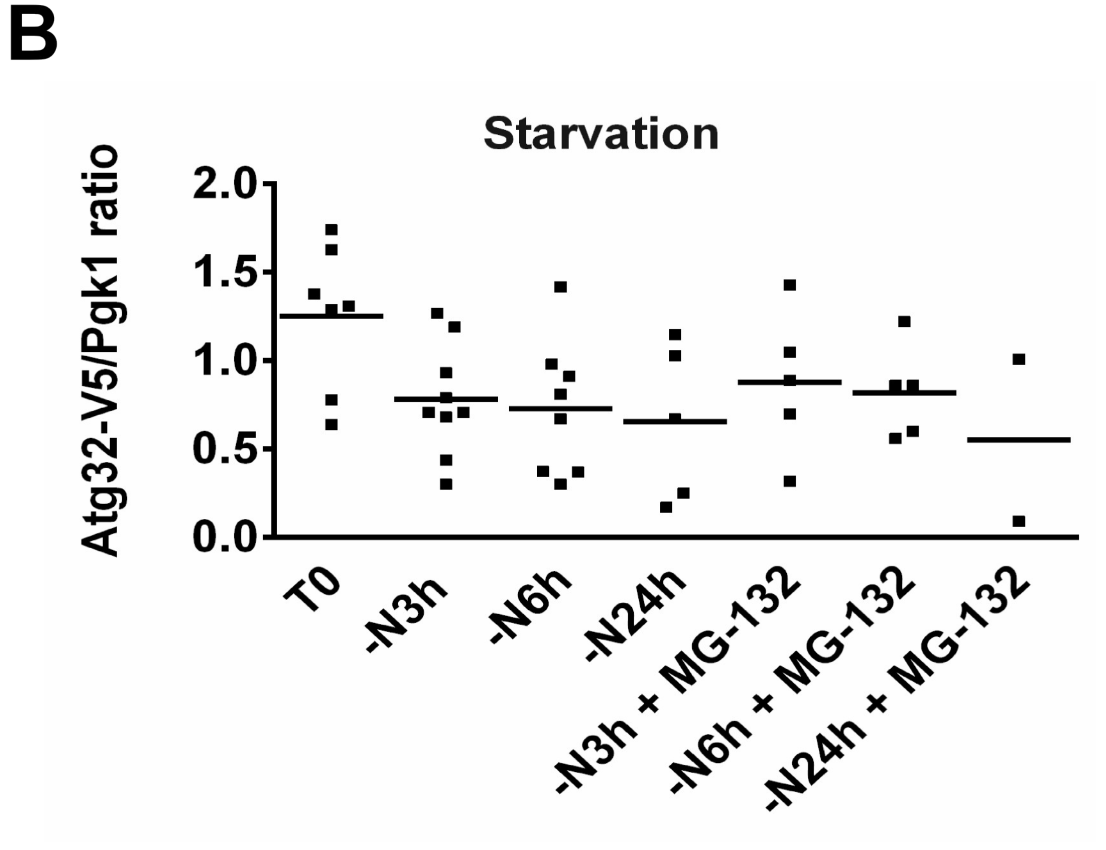
B
Starvation
Atg32-V5/Pgk1 ratio
2.0
1.5
1.0
0.5
0.0
T0
-N3h
-N6h
-N24h
-N3h + MG-132
-N6h + MG-132
-N24h + MG-132

Figure S2*:* The Atg32 protein is degraded upon nitrogen starvation. (A) *atg32Δ* mutant cells grown in a CMS-L medium and expressing Atg32-V5 protein were harvested at indicated times. To inhibit the proteasome, 75 μM MG-132 was added to the cell culture at the beginning of starvation. Total protein extracts were prepared afterwards, and samples were analyzed by western blots. Anti-V5 antibody was used to visualize Atg32-V5 protein; Pgk1 was used as a loading control. Anti-ubiquitin (Ub) was used to detect the level of ubiquitinated proteins. **(B)** The Atg32-V5/Pgk1 ratio was quantified for all tested conditions.
