# Supplementary material for: The yeast mitophagy receptor Atg32 is ubiquitinated and degraded by the proteasome
Source: PLoS One. 2020 Dec 23;15(12):e0241576. doi: 10.1371/journal.pone.0241576 (PMC7757876; doi:10.1371/journal.pone.0241576)
Supplement: S3 Fig — (A) atg5Δ, atg8Δ, and atg11Δ mutant cells transformed with a plasmid expressing Atg32-V5 were grown in a CMS-L medium. Cells were harvested at indicated times. (B) The Atg32-V5/Pgk1 ratios were quantified at T0 and 48 h time points for all tested strains; ** p<0.01. (C) atg5Δ mutant cells expressing Atg32-V5 were treated with MG-132 at time point 8 h. Cells were harvested at indicated time points and total protein extracts were prepared and analyzed by immunodetection. Anti-V5 antibody was used to visualize Atg32-V5 protein. (PDF) [file pone.0241576.s003.pdf]

Figure S3

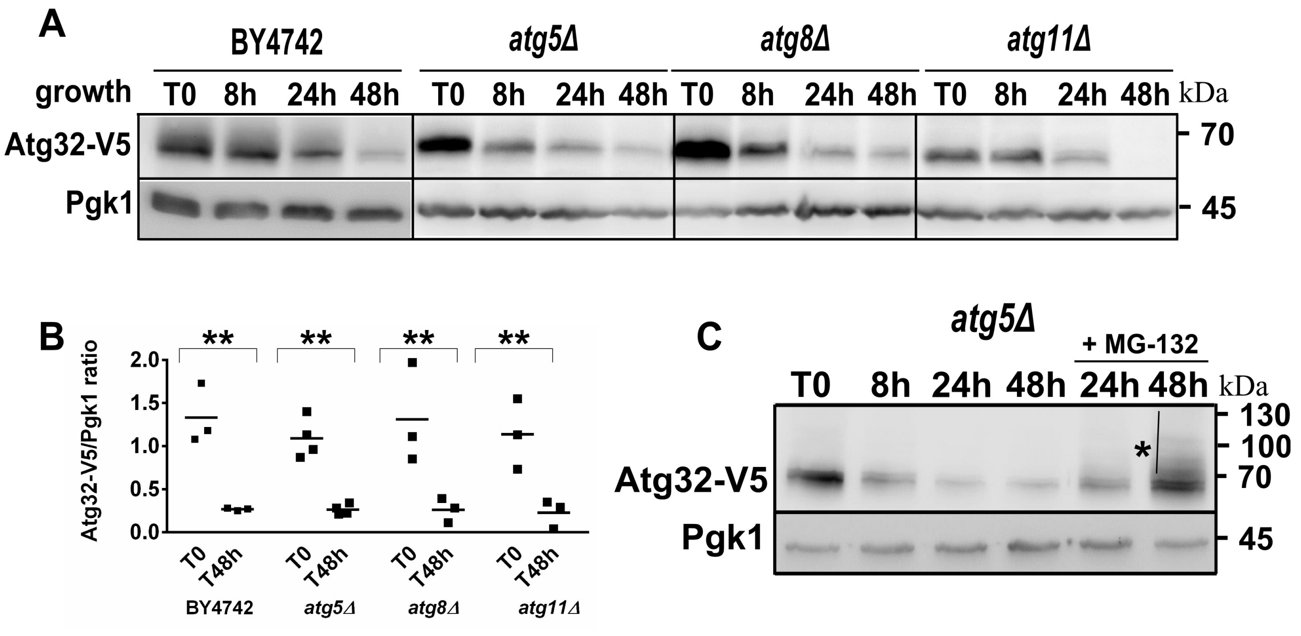

Figure S3: Degradation of Atg32 protein is not impaired in autophagy-deficient mutants under normal growth condition. (A) *atg5Δ*, *atg8Δ,* and *atg11Δ* mutant cells transformed with a plasmid expressing Atg32-V5 were grown in a CMS-L medium. Cells were harvested at indicated times. **(B)** The Atg32-V5/Pgk1 ratios were quantified at T0 and 48 h time points for all tested strains; ** $p<0.01$. **(C)** *atg5Δ* mutant cells expressing Atg32-V5 were treated with MG-132 at time point 8 h. Total protein extracts were prepared and analyzed by immunodetection. Anti-V5 antibody was used to visualize Atg32-V5 protein.
