# Supplementary material for: The yeast mitophagy receptor Atg32 is ubiquitinated and degraded by the proteasome
Source: PLoS One. 2020 Dec 23;15(12):e0241576. doi: 10.1371/journal.pone.0241576 (PMC7757876; doi:10.1371/journal.pone.0241576)
Supplement: S4 Fig — (A) The Atg32 protein is degraded upon rapamycin treatment and stabilized by the proteasome inhibition. atg32Δ mutant cells grown in a CMS-L medium and expressing Atg32-V5 protein were harvested at T0 and treated with 0.2 μg/ml rapamycin in presence or absence of 75 μM MG-132 for 3 h, 6 h, and 24 h. Total protein extracts were prepared afterwards, and samples were analyzed by immunodetection. Anti-V5 antibody was used to visualize Atg32-V5 protein. (B) The Atg32 protein is degraded in BY4742 strain. BY4742 cells transformed with a plasmid expressing Atg32-V5 grown in a CMS-L medium were harvested at indicated times. To inhibit proteasome, MG-132 was added to the cell culture at 8 h time point. (C) The Atg32-V5/Pgk1 ratios were quantify for all tested conditions—**P<0.01. (D) MG-123 stabilizes the Atg32 protein in exponentially growing cells. atg32Δ mutant cells grown in a CMS-L medium and expressing Atg32-V5 protein were harvested at T0 and treated with 75 μM MG-132. Cells were harvested at exponential (T8) and stationary (T24, T48) phase, and total protein extracts were prepared and analyzed by immunodetection. Anti-V5 antibody was used to visualize Atg32-V5 protein. (PDF) [file pone.0241576.s004.pdf]

Figure S4

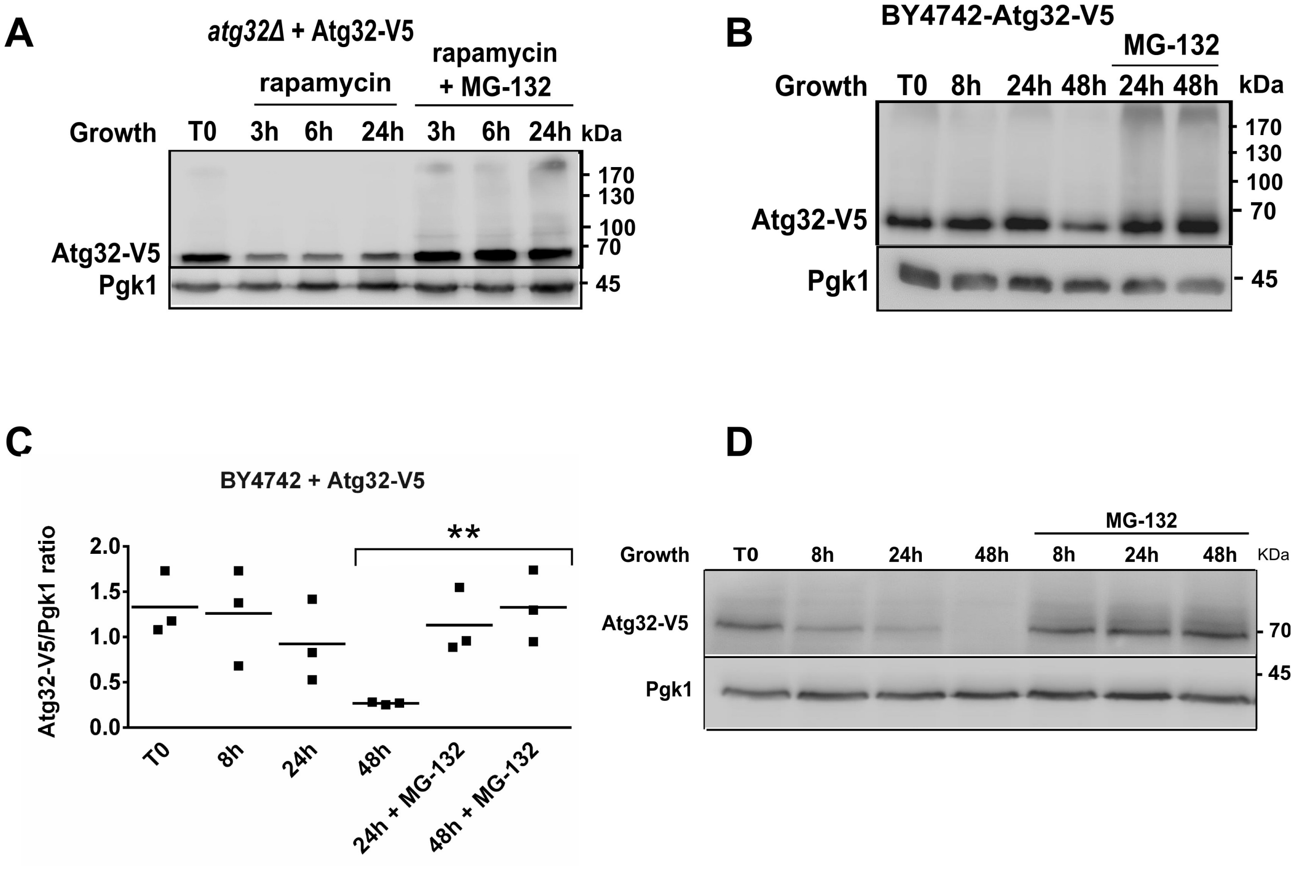

Figure S4: (A) The Atg32 protein is degraded upon rapamycin treatment and stabilized by the proteasome inhibition. *atg32Δ* mutant cells grown in a CMS-L medium and expressing Atg32-V5 protein were harvested at T0 and treated with 0.2 µg/ml rapamycin in presence or absence of 75 µM MG-132 for 3 h, 6 h, and 24 h. Total protein extracts were prepared afterwards, and samples were analyzed by immunodetection. Anti-V5 antibody was used to visualize Atg32-V5 protein. **(B) The Atg32 protein is degraded in BY4742 strain.** BY4742 cells transformed with a plasmid expressing Atg32-V5 grown in a CMS-L medium were harvested at indicated times. To inhibit proteasome, MG-132 was added to the cell culture at 8 h time point. Total protein extracts were prepared and analyzed by immunodetection. Anti-V5 antibody was used to visualize Atg32-V5 protein. **(C)** The Atg32-V5/Pgk1 ratios were quantify for all tested conditions— $^{**}P<0.01$. **(D) MG-123 stabilizes the Atg32 protein in exponentially growing cells.** *atg32Δ* mutant cells grown in a CMS-L medium and expressing Atg32-V5 protein were harvested at T0 and treated with 75 µM MG-132. Cells were harvested at exponential (T8) and stationary (T24, T48) phase, and total protein extracts were prepared and analyzed by immunodetection. Anti-V5 antibody was used to visualize Atg32-V5 protein.
