# Supplementary material for: The yeast mitophagy receptor Atg32 is ubiquitinated and degraded by the proteasome
Source: PLoS One. 2020 Dec 23;15(12):e0241576. doi: 10.1371/journal.pone.0241576 (PMC7757876; doi:10.1371/journal.pone.0241576)
Supplement: S5 Fig — (A) Addition of proteasome inhibitor MG-132 (75 μM MG-132) and inhibitor of vacuolar proteolysis PMSF (2 mM) do not affect growth and growth yield in atg32Δ mutant cells expressing Atg32-V5 plasmid and grown in a CMS-L medium. The Y-axis is represented in logarithmic scale (n = 5 for control and MG-132; n = 3 for PMSF). (B) atg32Δ cells grown in a CMS-L medium and expressing Atg32-V5 protein were harvested at indicated time points. To inhibit proteasome, MG-132 was added to the cell culture at 8 h time point. To inhibit vacuolar proteolysis, 2 mM PMSF was added to the cell culture at T8; this step was repeated twice during the course of cell growth. Total protein extracts were prepared afterwards, and samples were analyzed by western blots. Anti-V5 antibody was used to visualize Atg32-V5 protein. To detect modified Atg32-V5 forms (bands with a higher molecular weight) after MG-132 treatment, two different revelation times of blots are presented. (PDF) [file pone.0241576.s005.pdf]

Figure S5

A

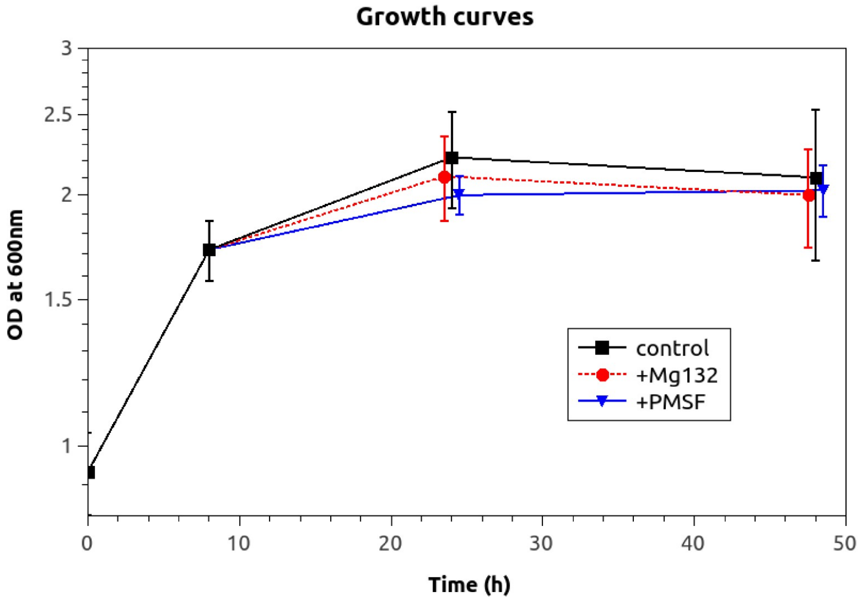

B

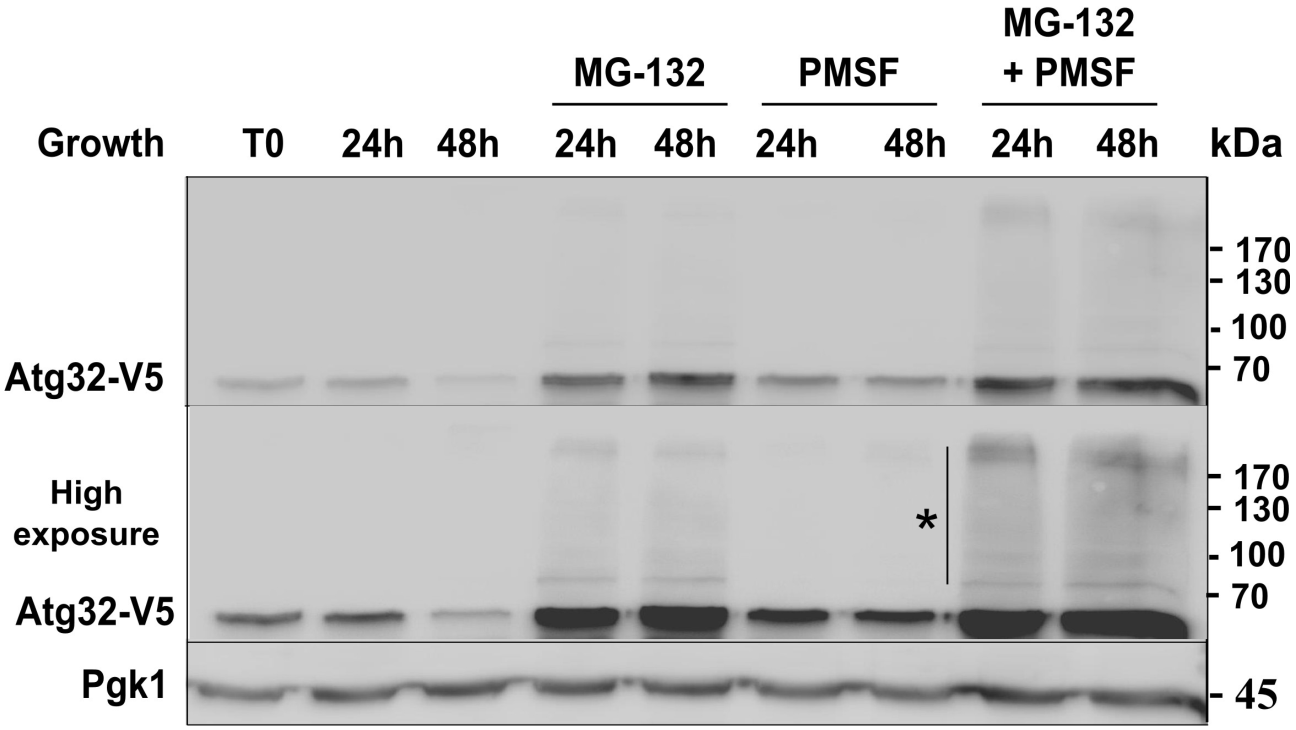

Figure S5: The effect of MG-132 and PMSF treatment on cell growth and Atg32-V5 protein degradation. (A) Addition of proteasome inhibitor MG-132 (75 µM MG-132) and inhibitor of vacuolar proteolysis PMSF (2 mM) do not affect growth and growth yield in *atg32Δ* mutant cells expressing Atg32-V5 plasmid and grown in a CMS-L medium. The Y-axis is represented in logarithmic scale (n = 5 for control and MG-132; n = 3 for PMSF). **(B)** *atg32Δ* cells grown in a CMS-L medium and expressing Atg32-V5 protein were harvested at indicated time points. To

inhibit proteasome, MG-132 was added to the cell culture at 8 h time point. To inhibit vacuolar proteolysis, 2 mM PMSF was added to the cell culture at T8; this step was repeated twice during the course of cell growth. Total protein extracts were prepared afterwards, and samples were analyzed by western blots. Anti-V5 antibody was used to visualize Atg32-V5 protein. To detect modified Atg32-V5 forms (bands with a higher molecular weight) after MG-132 treatment, two different revelation times of blots are presented.
