# Supplementary material for: The yeast mitophagy receptor Atg32 is ubiquitinated and degraded by the proteasome
Source: PLoS One. 2020 Dec 23;15(12):e0241576. doi: 10.1371/journal.pone.0241576 (PMC7757876; doi:10.1371/journal.pone.0241576)
Supplement: S6 Fig — BY4742 (A) and atg32Δ mutant (B) cells expressing GFP-Atg8 protein grown in a CMS-L medium in presence or absence of MG-132 were harvested at indicated times. Total protein extracts from 2 x 10^7^ cells were prepared and separated by 12.5% SDS-PAGE gel as described in the Material and Methods section. Proteins were detected using antibodies against GFP or Pgk1. (PDF) [file pone.0241576.s006.pdf]

Figure S6

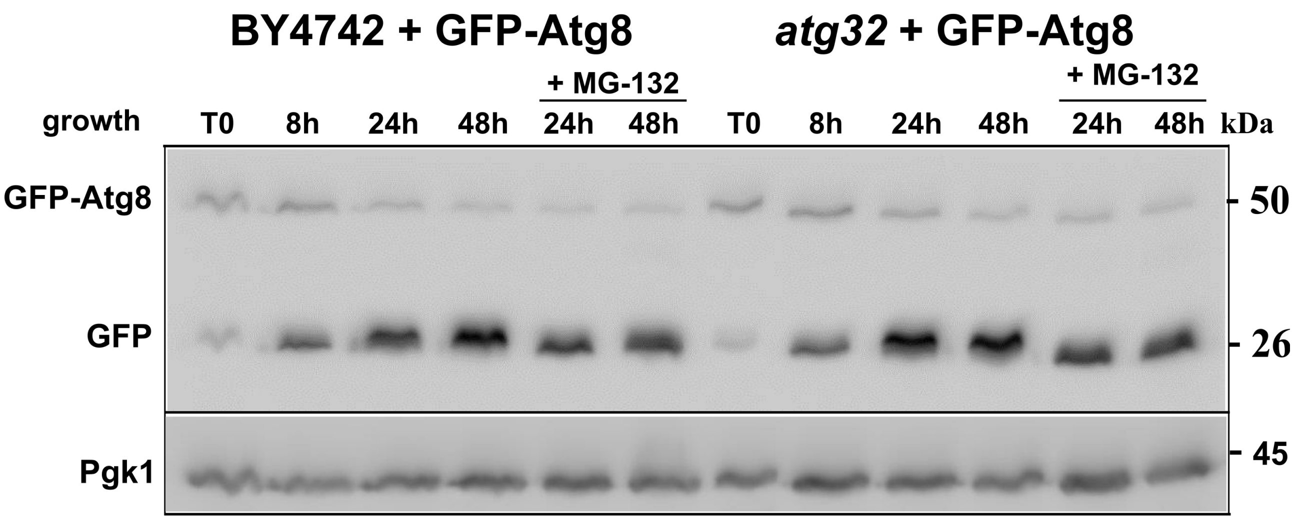

Figure S6 : Inhibition of the proteasome with MG-132 does not affect autophagy. BY4742 **(A)** and *atg32Δ* mutant **(B)** cells expressing GFP-Atg8 protein grown in a CMS-L medium in presence or absence of MG-132 were harvested at indicated times. Total protein extracts from 2 x 10^7 cells were prepared and separated by 12.5% SDS-PAGE gel as described in the Material and Methods section. Proteins were detected using antibodies against GFP or Pgk1.
