# Supplementary material for: The yeast mitophagy receptor Atg32 is ubiquitinated and degraded by the proteasome
Source: PLoS One. 2020 Dec 23;15(12):e0241576. doi: 10.1371/journal.pone.0241576 (PMC7757876; doi:10.1371/journal.pone.0241576)
Supplement: S7 Fig — (A) Lysate from atg32Δ mutant cells expressing pATG32-V5-6HIS was prepared as described in the Material and Methods section. Next, lysate was loaded on a Ni-NTA column, the non-retained fraction, as well as the two washes W1 and W2, were recovered. The bounded proteins were then eluted and 500 μl fractions were collected. (B-D) 250 μl of each fraction absorbing at 254 nm (from F12 to F22) were precipitated with TCA. Pellets were resuspended in 20 μl of the loading buffer; 10 μl were loaded on the gel to be revealed with the colloidal blue (B) and 5 μl were used for immunodetection with anti-histidine (C) or anti-ubiquitin antibodies (D), respectively. (PDF) [file pone.0241576.s007.pdf]

Figure S7

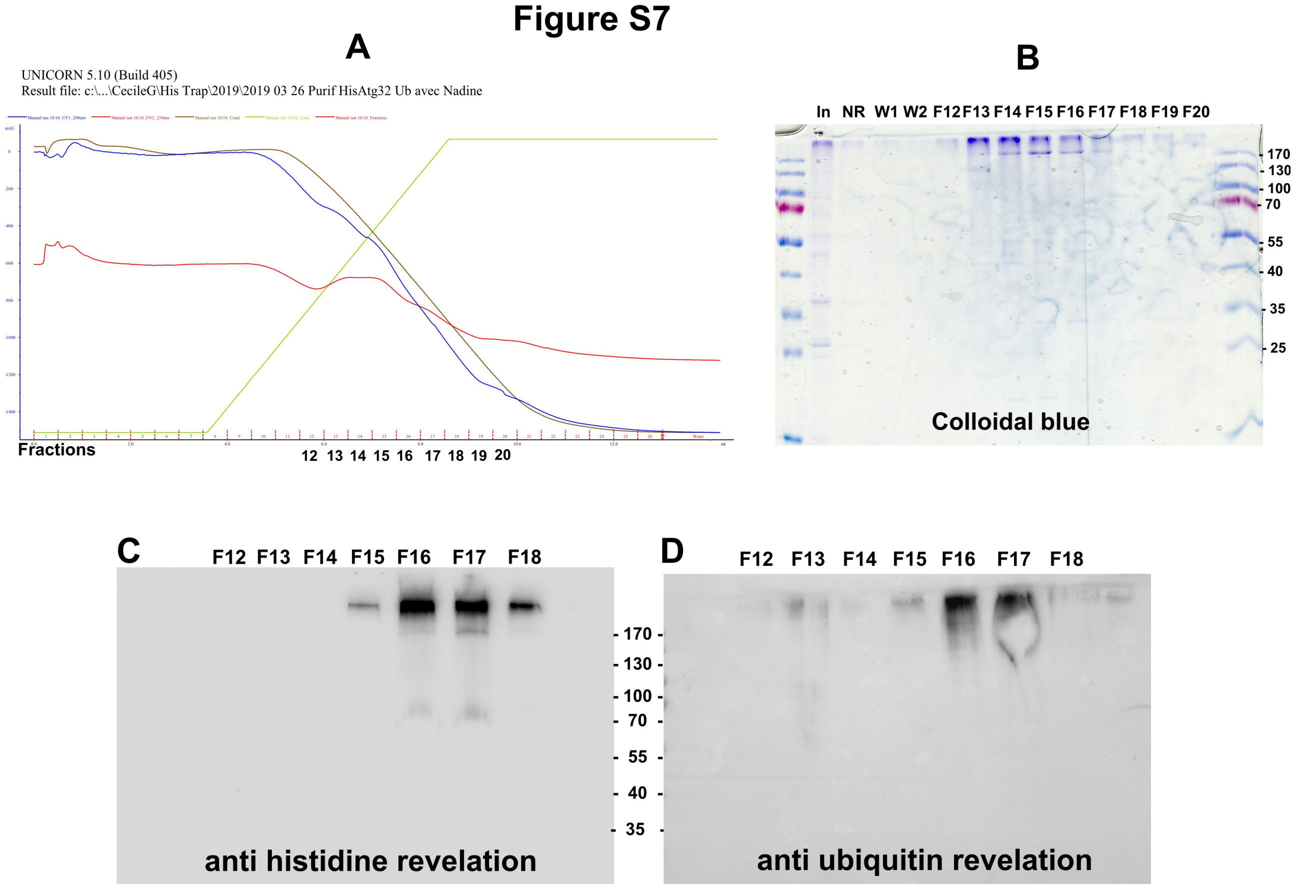

Figure S7: Purification of Atg32-V5-6HIS. (A) Lysate from *atg32Δ* mutant cells expressing *pATG32-V5-6HIS* was prepared as described in the Material and Methods section. Next, lysate was loaded on a Ni-NTA column, the non-retained fraction, as well as the two washes W1 and W2, were recovered. The bounded proteins were then eluted and 500 µl fractions were collected. **(B-D)** 250 µl of each fraction absorbing at 254 nm (from F12 to F22) were precipitated with TCA. Pellets were resuspended in 20 µl of the loading buffer; 10 µl were loaded on the gel to be revealed with the colloidal blue (B) and 5 µl were used for immunodetection with anti-histidine (C) or anti-ubiquitin antibodies (D), respectively
