# Supplementary material for: The yeast mitophagy receptor Atg32 is ubiquitinated and degraded by the proteasome
Source: PLoS One. 2020 Dec 23;15(12):e0241576. doi: 10.1371/journal.pone.0241576 (PMC7757876; doi:10.1371/journal.pone.0241576)
Supplement: S8 Fig — (A) SEQUEST spectra, (B) MASCOT spectra, and (C) Protein coverage. (PDF) [file pone.0241576.s008.pdf]

Figure S8

Peptide SEQUEST

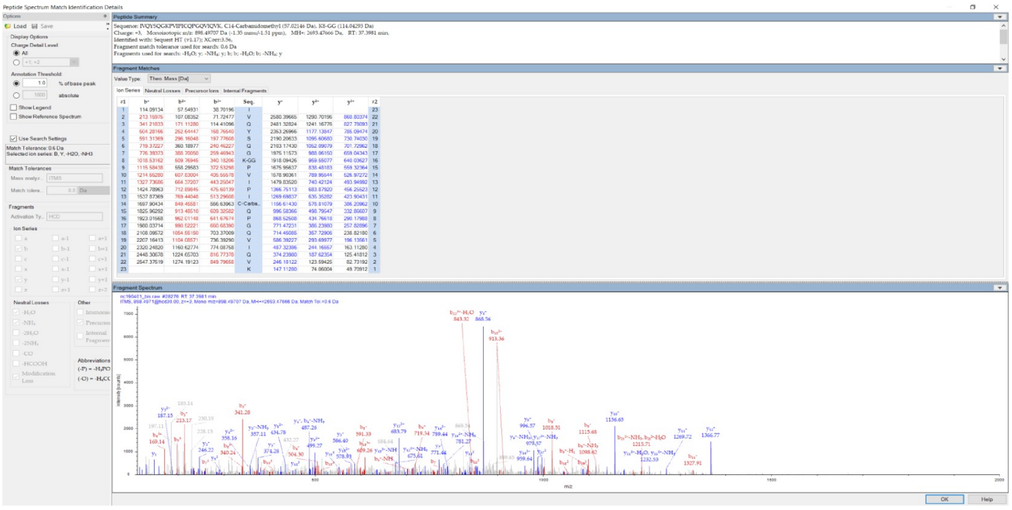

Peptide MASCOT

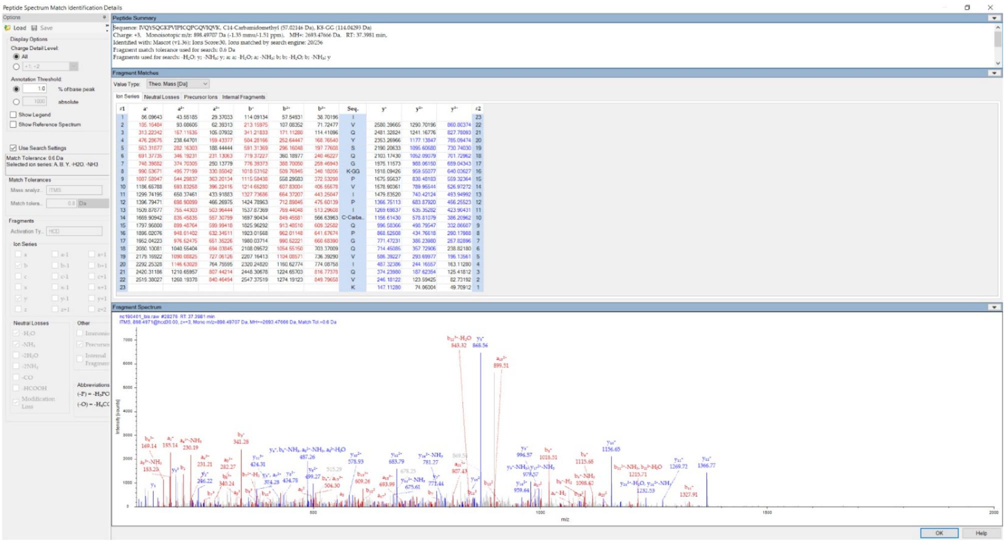

Protein coverage

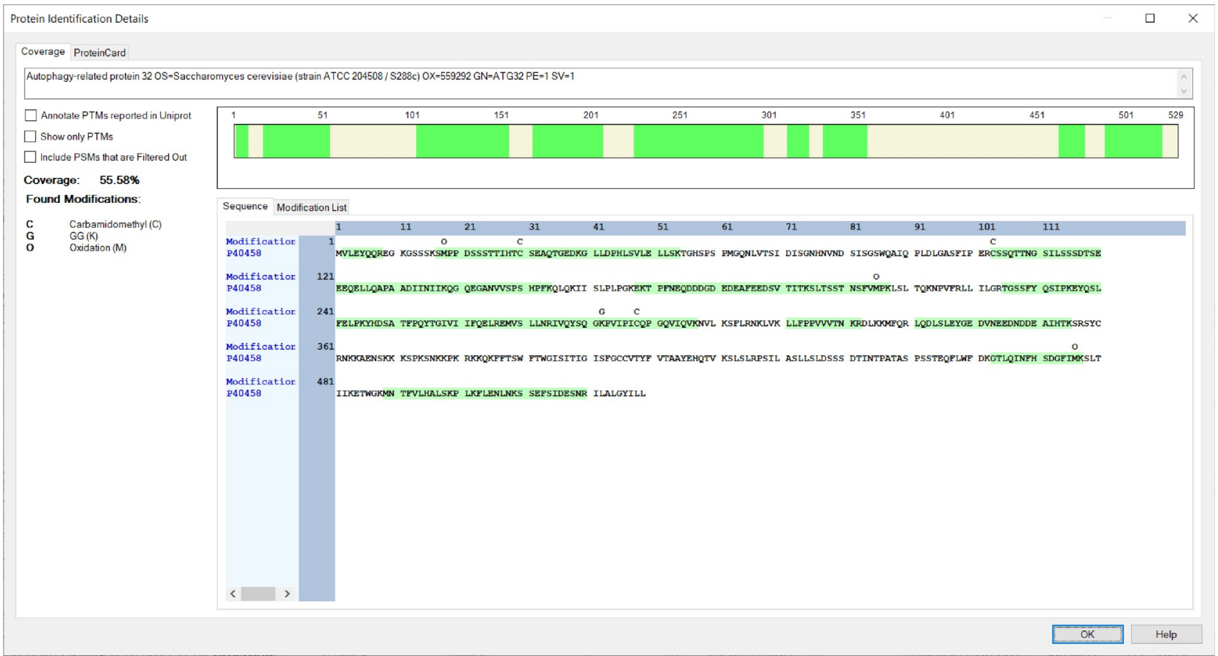

Figure S8: Mass spectrometry analysis. (A) SEQUEST spectra, (B) MASCOT spectra, and (C) Protein coverage.
