# Supplementary material for: The yeast mitophagy receptor Atg32 is ubiquitinated and degraded by the proteasome
Source: PLoS One. 2020 Dec 23;15(12):e0241576. doi: 10.1371/journal.pone.0241576 (PMC7757876; doi:10.1371/journal.pone.0241576)
Supplement: S1 Table — (PDF) [file pone.0241576.s009.pdf]

Supplementary Table I

STRAINS

**Souche**	**Genotype**
BY4742 + Idp1-GFP+ Atg32-V5	Mat α ; *his3Δ1;leu2Δ0 ; lys2Δ0 ; ura3Δ0 ; pIDP1-GFP, pYES-Atg32-V5*
BY4742 + pPROM-*ATG32-β-galactosidase,*	Mat α ; *his3Δ1;leu2Δ0 ; lys2Δ0 ; ura3Δ0 ;* YEp357-prom*ATG32-lacZ*
Δ*atg32* + Idp1-GFP+ Atg32-V5	BY4742 *atg32::kanMX4* p*IDP1-GFP, pYES-Atg32-V5*
Δ*atg32* + HA- Atg32-V5	BY4742 *atg32::kanMX4* p*HA-Atg32*
Δ*atg32* + Idp1-GFP	BY4742 *atg32::kanMX4* p*IDP1-GFP*
Δ*atg5* + Atg32-V5	BY4742 *atg5::kanMX4 pYES-Atg32-V5*
Δ*atg8* + Atg32-V5	BY4742 *atg8::kanMX4, ppYES-Atg32-V5*
Δ*atg11*+ Atg32-V5	BY4742 *atg11::kanMX4, pYES-Atg32-V5*
Δ*atg32* + Idp1-GFP	BY4742 *atg32::kanMX4, pIDP1-GFP*
pre2-2 + Atg32-V5	BY4742 *his3D1; leu2D0; met15D0; ura3D0 pre2-2::KanMX4 pYES-Atg32-V5*
Δ*atg32* + Atg32-V5 K282A	BY4742 *atg32::kanMX4* p*IDP1-GFP, pAtg32-V5 lysine 282 replaced by alanine*
Δ*atg32* + Atg32-V5 mutated RSP5 motif	BY4742 *atg32::kanMX4* p*IDP1-GFP, pAtg32-V5 motif L263P264K265Y266 replaced by A263A264A265A266*
Δ*atg32*Δ*pho8* + mtPho8 +Atg32-V5	BY4742 *atg32::kanMX4 pho8::HIS3* pFL39-*COXIV-PHO8Δ60; pYES-Atg32-V5*
