# Supplementary material for: The yeast mitophagy receptor Atg32 is ubiquitinated and degraded by the proteasome
Source: PLoS One. 2020 Dec 23;15(12):e0241576. doi: 10.1371/journal.pone.0241576 (PMC7757876; doi:10.1371/journal.pone.0241576)
Supplement: S1 File — (PDF) [file pone.0241576.s010.pdf]

Data for the graphs

Figure 2B
***atg32Δ* + Atg32-V5 strain: growth**

T0	1.14	0.927	1.37	0.91	0.78	0.69	1.32	1.15
8h	1.05	0.28	0.75	1.1	1.33			
24h	0.32	0.069	1.27	1.1	0.73	0.335	0.39	0.33
48h	0.196	0.11	0.23	0.28	0.175	0.112	0.24	0.1

Figure 2D
HA-Atg32 level during growth

T0	1.25	1.42	1.87	1.28	1.29
8h	0.71	1.37	1.36	1.09	0.88
24h	0.54	0.8	1.14	0.78	0.55
48h	0.37	0.58	0.44	0.25	0.24

Figure 3B
***atg32Δ* + Atg32-V5 strain:**

T0	1.14	0.927	1.37	0.91	0.78	0.69	1.32	1.15
8h	1.05	0.28	0.75	1.1	1.33			
24h	0.32	0.069	1.27	1.1	0.73	0.335	0.39	0.33
48h	0.196	0.11	0.23	0.28	0.175	0.112	0.24	0.1
24h + MG132	0.938	1.55	1.62	0.94	0.94			
48h + MG132	1.22	1.28	1.6	1.01	1.2			
24h + PMSF	0.31	0.43	0.297					
48h + PMSF	0.09	0.33	0.418					

Figure 3C

BY4742+ Atg32-V5 and *pep4Δ* + Atg32-V5 strains

	**BY4742 + Atg32-V5**				***pep4Δ* + Atg32-V5**			
T0	0.66	1.19	0.84	0.73	1.07	2.11	0.91	0.99
48h	0.38	0.1	0.1	0.37	0.31	0.57	0.4	0.36

Figure 4A

ATG32 Promoter activity

T0	100%							
24h	200%	150%	200%	110%	80%	145%		
48h	380%	580%	440%	298%	610%	330%	346%	235%
48h + MG132	103%	80%	300%	410%	75%	60%	40%	

Figure 4C
Cycloheximide treatment

T0	0,98	0,9	1,01	1,6	1,4
20 min	0,66	0,74	0,36		
20 min + MG-132	1,34	0,64	0,8	0,76	
40 min	0,507	0,26	0,32		
40 min + MG-132	0,58	0,68	0,7	0,83	
1h	0,26	0,24	0,37	0,05	
1h + MG-132	1,1	0,99	0,92	0,98	

Figure 5B

***atg32Δ* + Atg32-V5 and pre2-2 strains**

	***atg32Δ* + Atg32-V5**				***pre2-2* + Atg32-V5**			
T0	0.99	1.98	0.61	2	0.72	1.8	1.39	2.1
48h	0.31	0.3	0.17	0.14	1.22	0.94	1.04	1.47

Figure 6C

	***atg32Δ* + Atg32-V5**				**BY4742 + Atg32-V5**			
24h	0,251	0,176	0,43	0,05	0,124	0,033	0,397	
24h + MG-132	0,5	0,255	0,256	0,418	0,223	0,391		
48h	0,435	0,426	0,062	0,216	0,179	0,127	0,452	
48h + MG6132	0,292	0,718	0,296	0,421	0,244	0,465		

Figure 6D

BY4742: Mt PHO8 activity

T0	0.458	0.445	0.376	0.368	0.376	0.364
48h	0.99	1.08	0.75	0.73		
48h + MG132	1.89	2.14	1.68	1.72		

Figure 8C

Atg32-V5 levels in *atg32* mutants

Atg32 + atg32-V5	T0	2.22	1.24	0.99	1.98	1,14	0,37
	48h	0	0.02	0.17	0.14	0,175	0,11
Atg32 + atg32-V5 K282A	T0	1,25	1,71	1,58	1,71	1,39	1,02
	48h	0,27	0,48	0,21	0,28	0,38	0,39
Atg32 + atg32-V5 AAAA	T0	1.03	1.13	0.95	1.13		2.08
	48h	0.18	0.19	0.25	0.21		0.16

Figure 9B

Mitophagy in *atg32* mutants

Atg32 + atg32-V5	0.159	0.137	0.117	0.3	0.157
Atg32 + atg32-V5 K282A	0.144	0.124	0.184	0.23	
Atg32 + atg32-V5 AAAA	0.186	0.203	0.173	0.27	

Figure S2B

***atg32Δ* + Atg32-V5 strain: starvation**

T0	100%					
-N3h	53%	70%	53%	98%	70.7%	95%
-N6h	68%	94%	56%	45%	80%	44%
-N24h	35%	12.8%	32%	53%	21.4%	57.6%
-N3h + MG132	141%	68%	129%			
-N6h + MG132	132%	64.3%	109%	117%		
-N24h + MG132	62%	10.8%	67.6%			

Figure S3B

Atg32-V5 levels in autophagy mutants

BY4742	T0	1.08	1.18	1.73	
	48h	0.25	0.27	0.28	
atg5Δ	T0	1.13	0.96	1.4	0.87
	48h	0.21	0.34	0.22	0.28
atg8Δ	T0	1.1	1.97	0.85	
	48H	0.28	0.29	0.11	
atg11Δ	T0	1.13	0.73	1.55	
	48H	0.35	0.04	0.29	

Figure S4C

Atg32-V5 levels in BY4742

T0	1.08	1.18	1.73
8h	1.73	1.38	0.68
24h	1.42	0.83	0.53
48h	0.25	0.27	0.28
24h + MG132	0.89	1.55	0.96
48h + MG132	1.3	1.74	0.95
