# Supplementary material for: The yeast mitophagy receptor Atg32 is ubiquitinated and degraded by the proteasome
Source: PLoS One. 2020 Dec 23;15(12):e0241576. doi: 10.1371/journal.pone.0241576 (PMC7757876; doi:10.1371/journal.pone.0241576)
Supplement: S2 File — (PDF) [file pone.0241576.s011.pdf]

Figure 1A

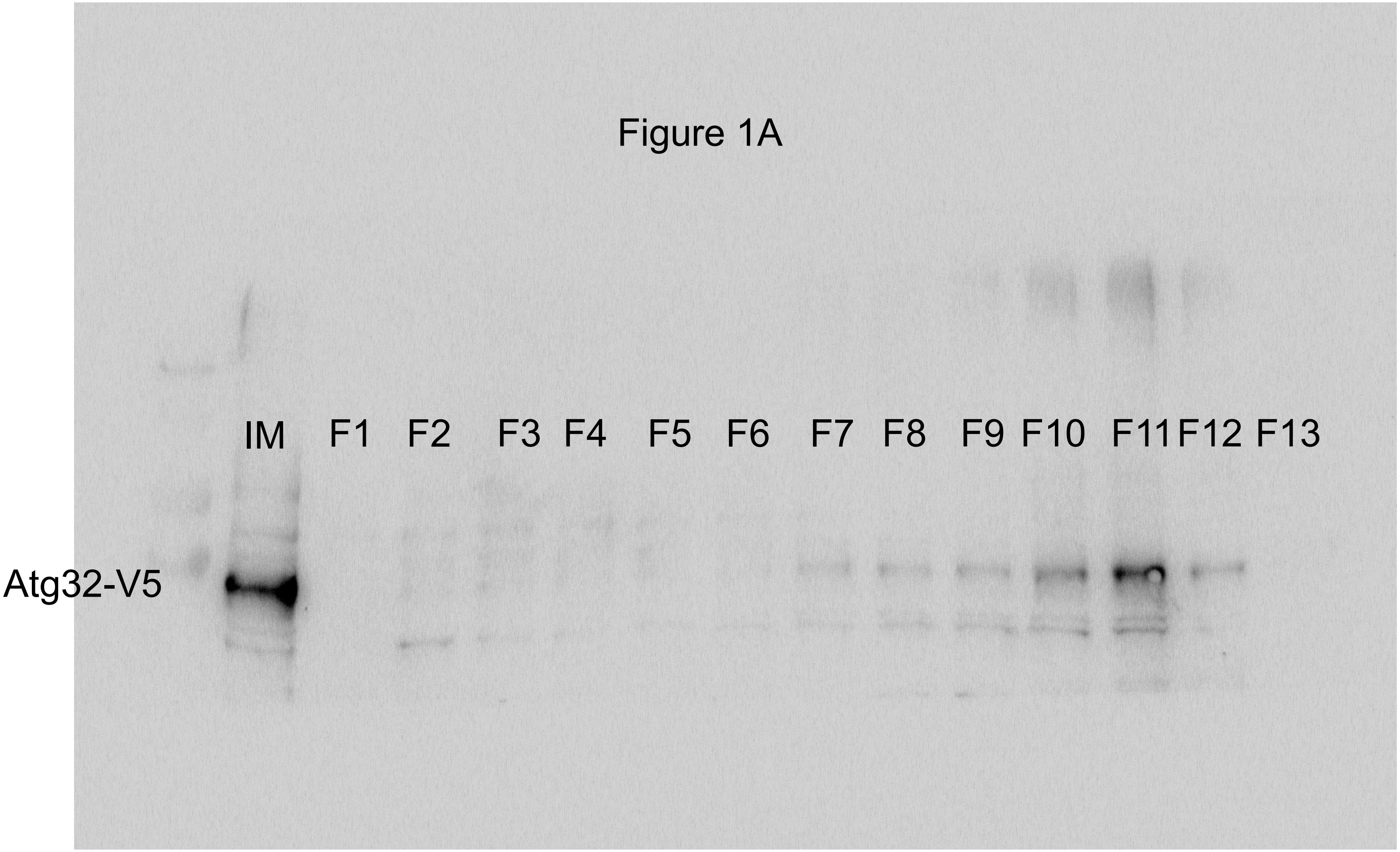

Figure 1A

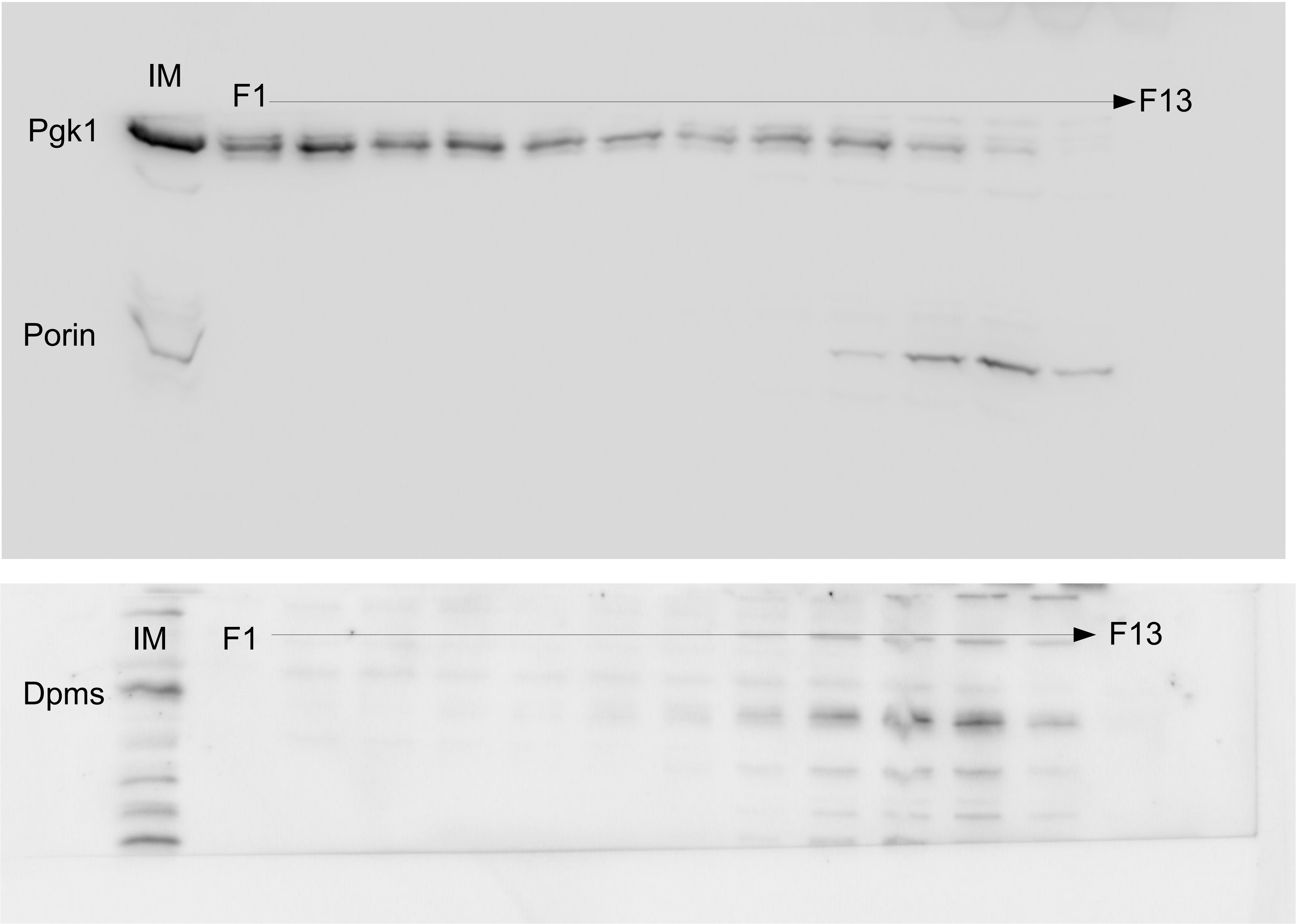

Figure 1B : GFP blotting

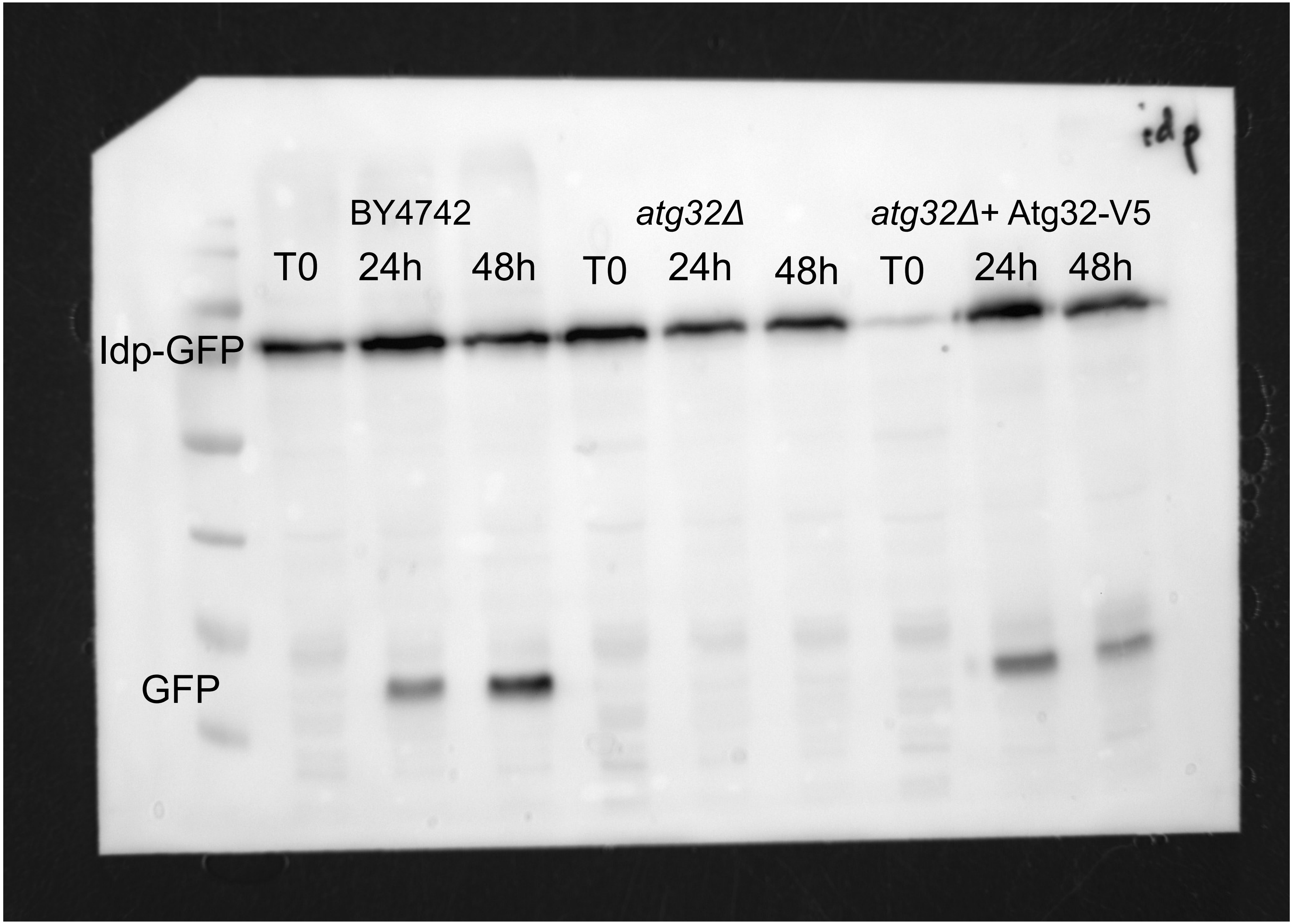

Figure 1B : Pgk1 blotting

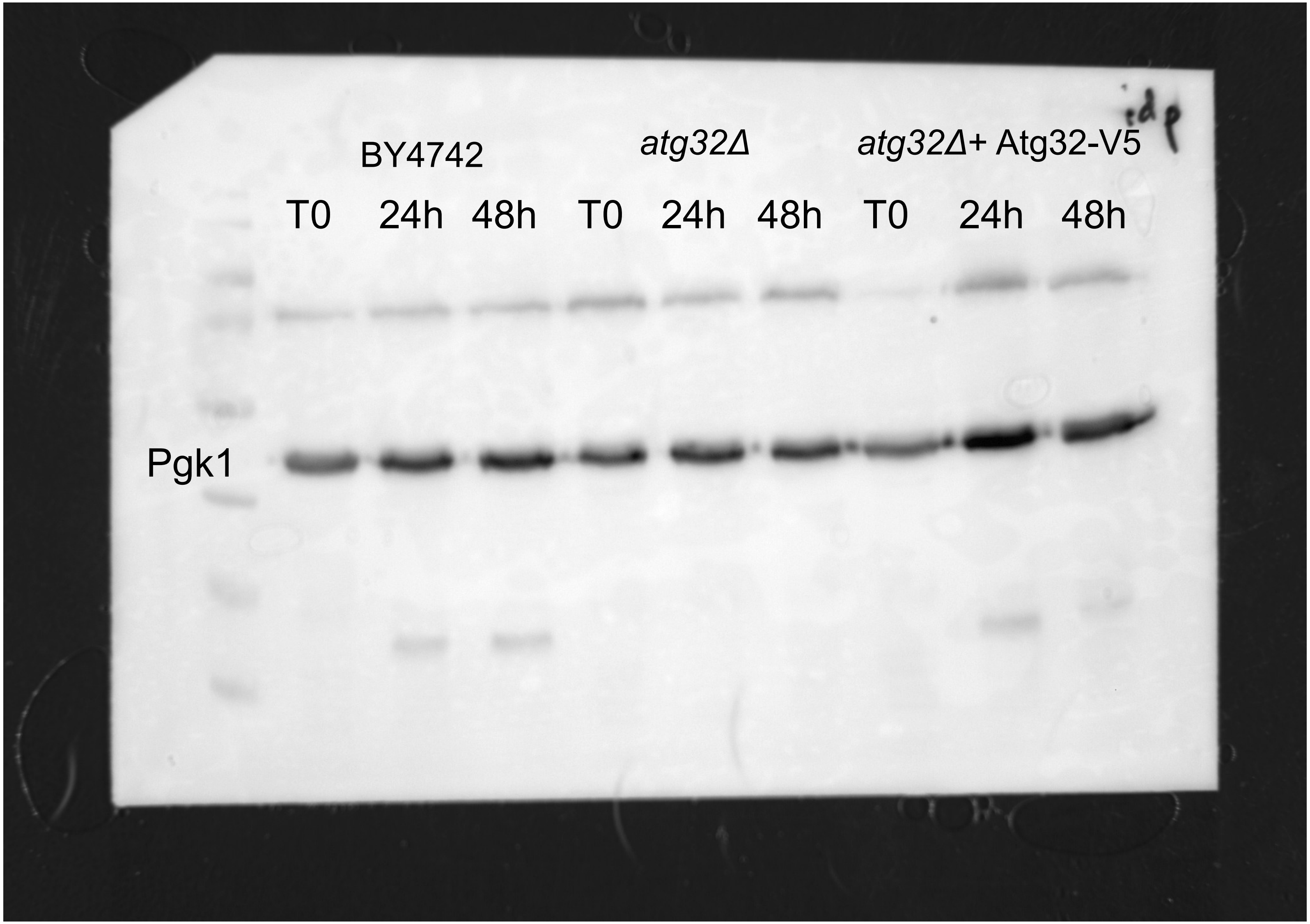

Figure 2A : V5 blotting

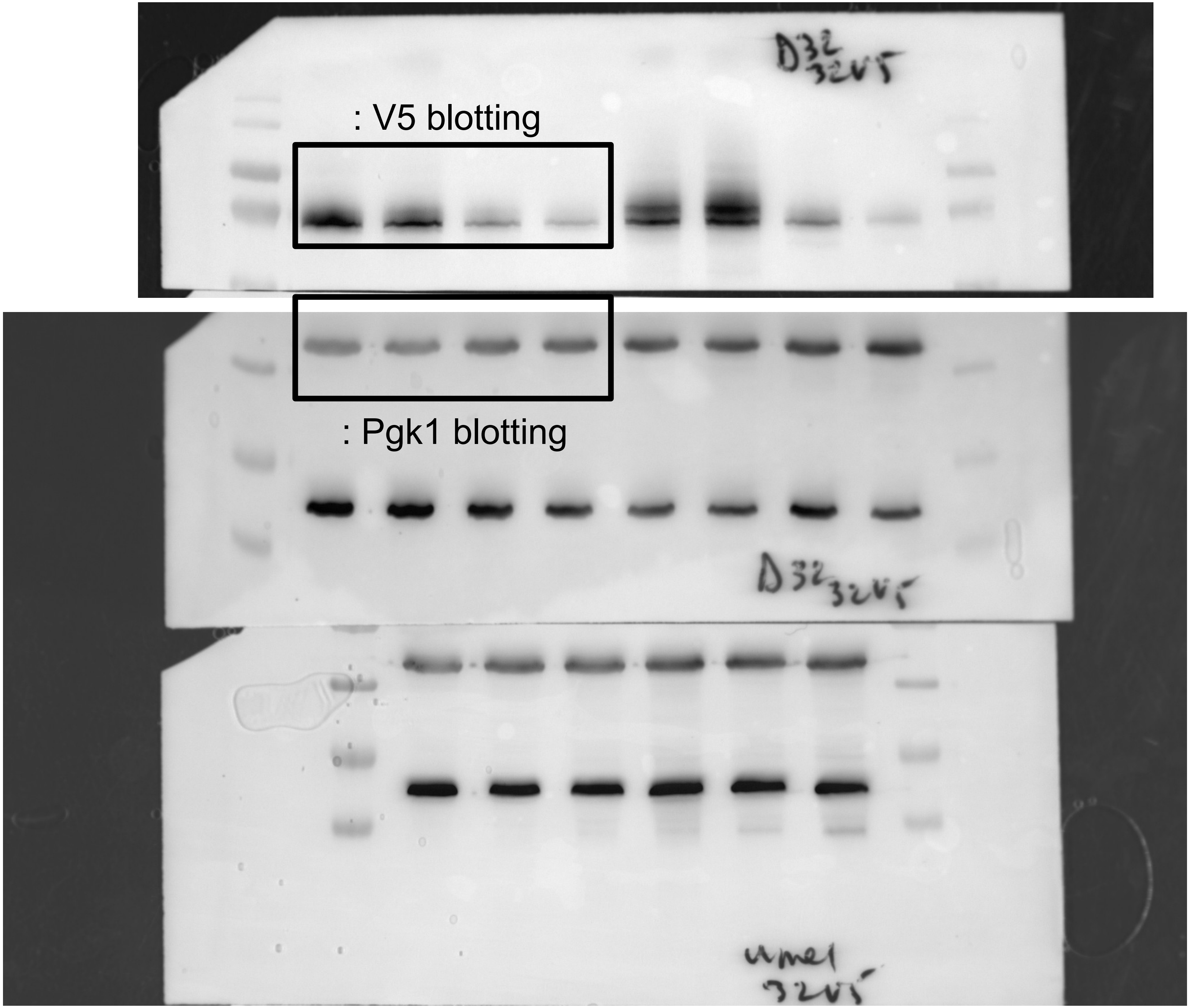

Figure 2C : HA blotting

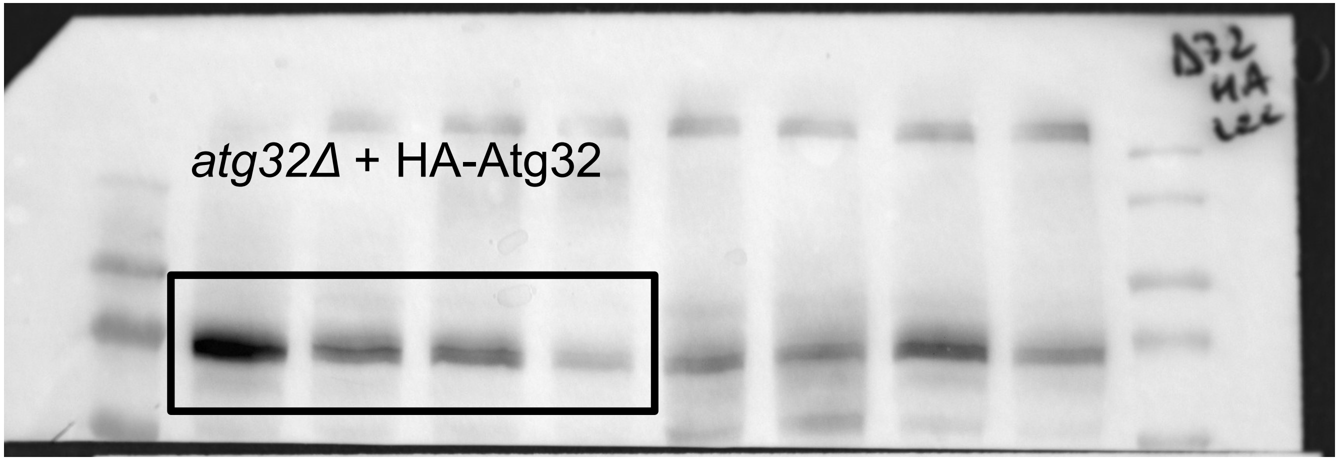

Figure 2C : Pgk1blotting

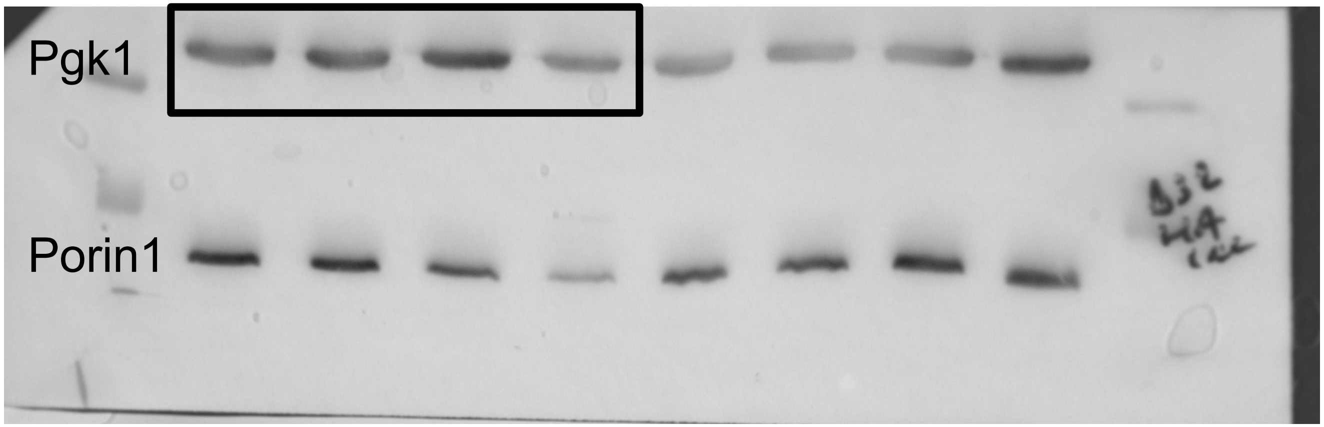

Figure 3A : V5 blotting

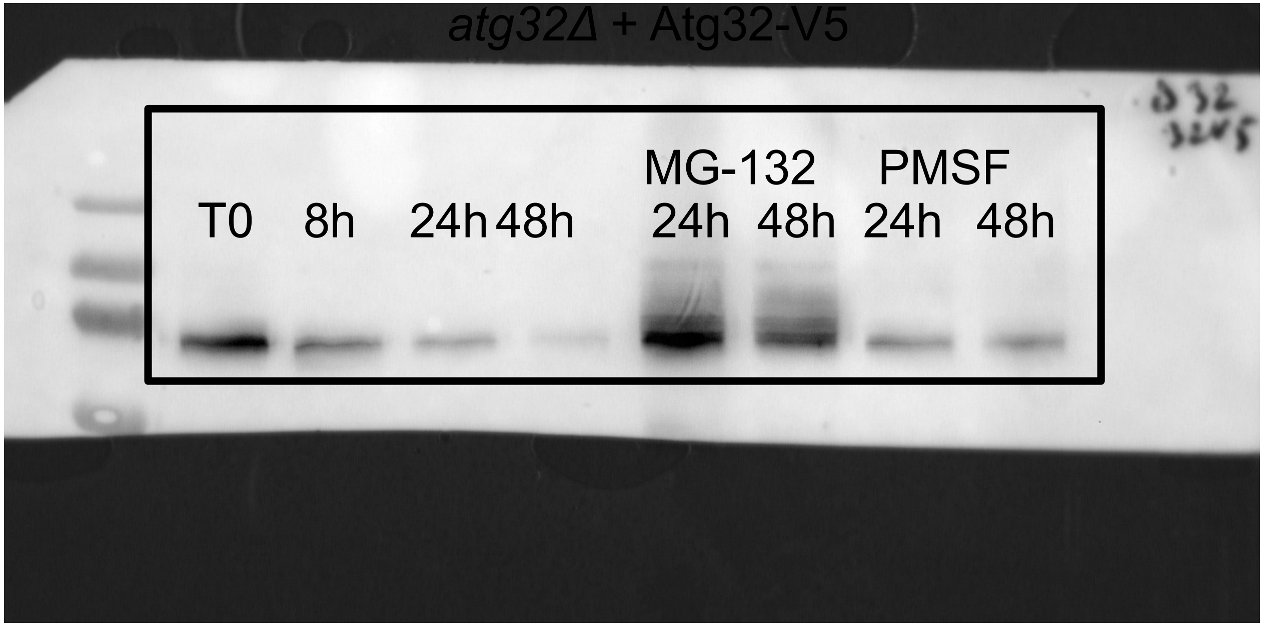

Figure 3A : Ubiquitin blotting

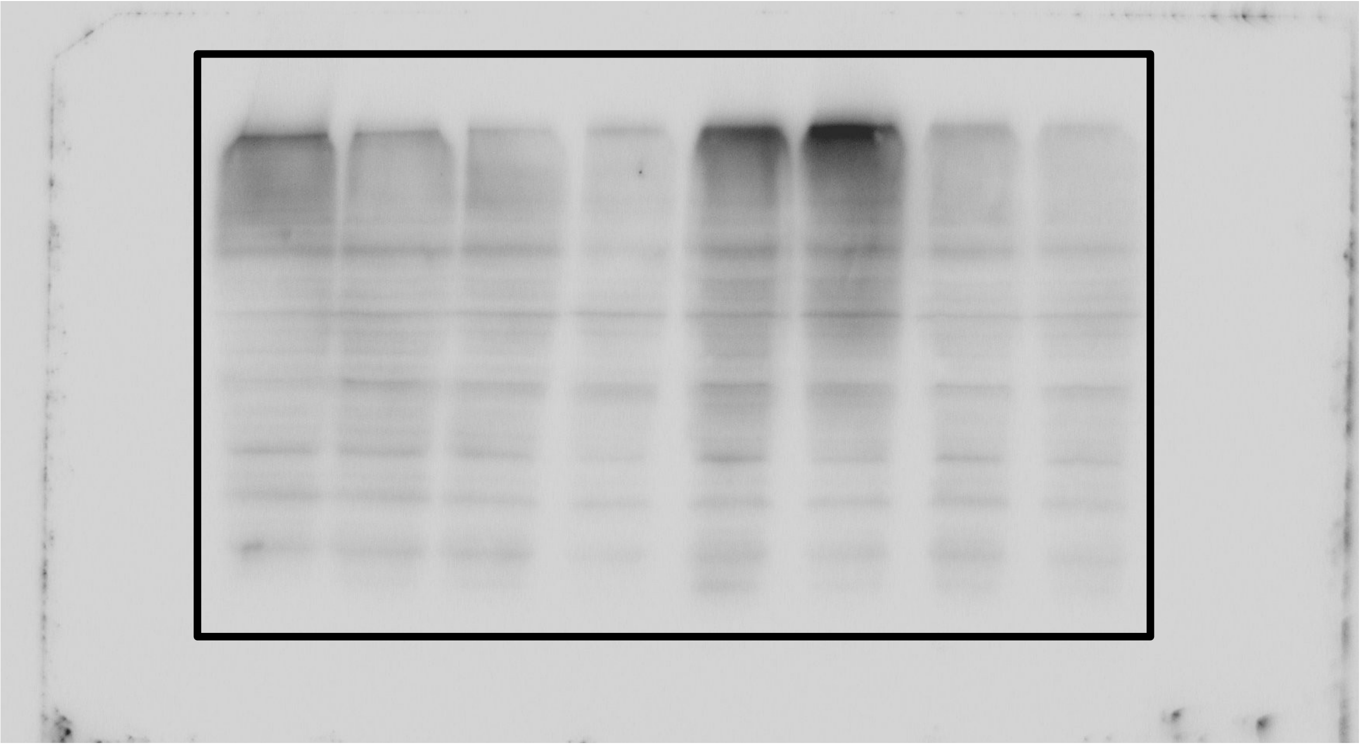

Figure 3A : Pgk1 blotting

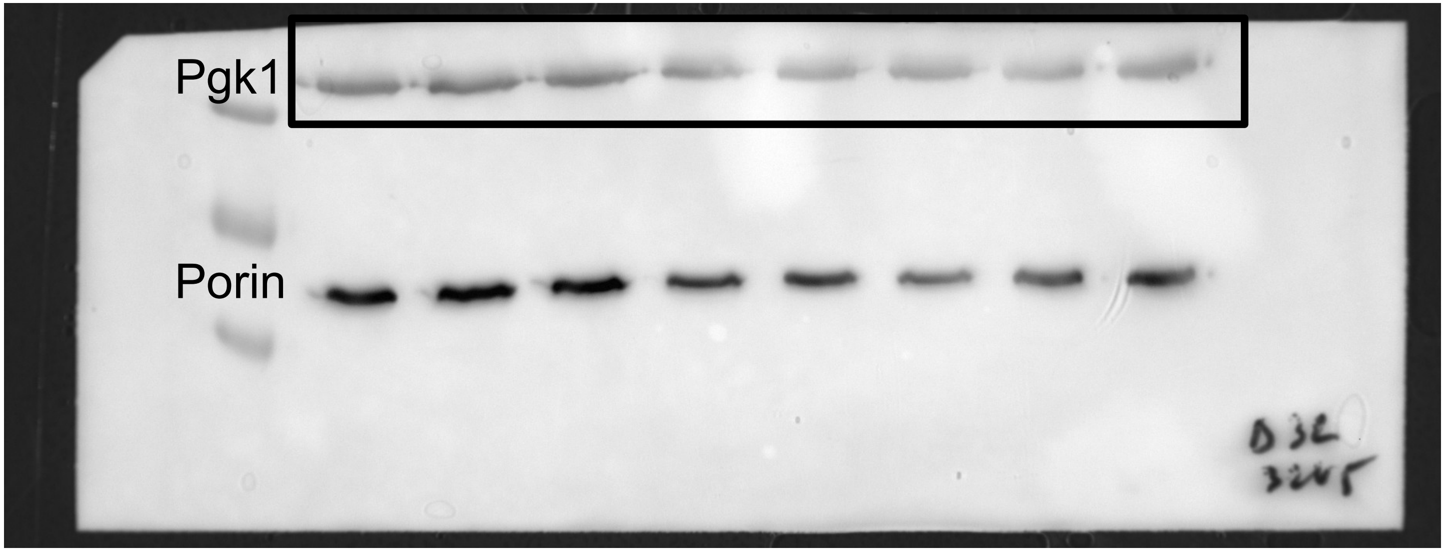

Figure 3C : V5 blotting

Figure 3C : Pgk1 blotting

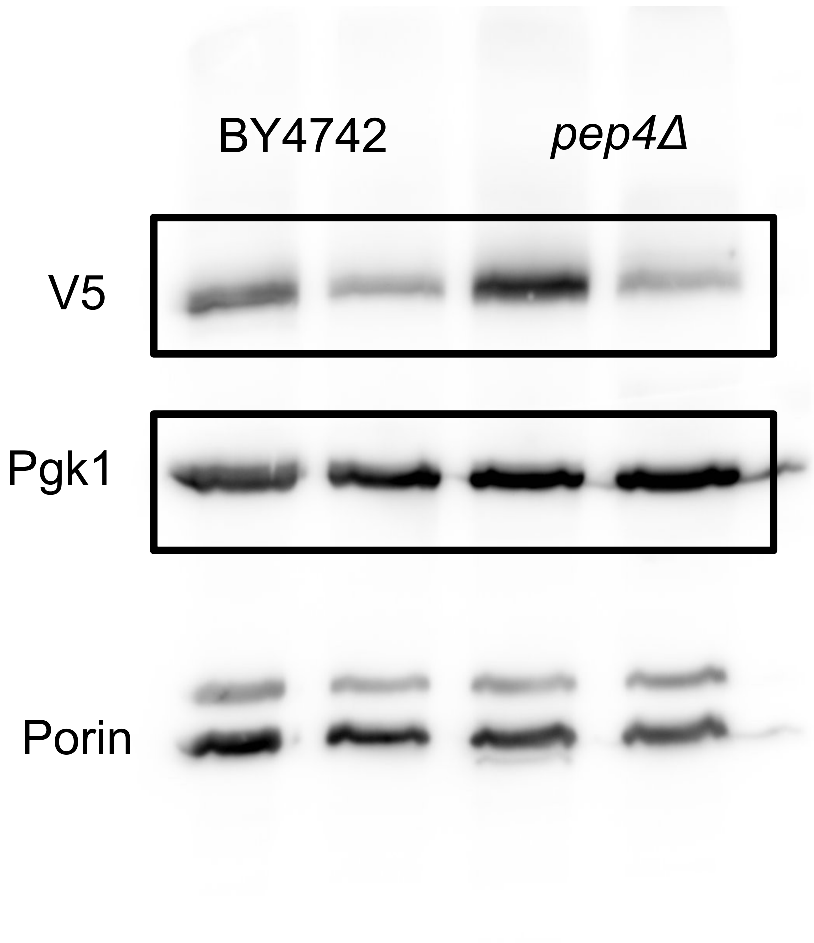

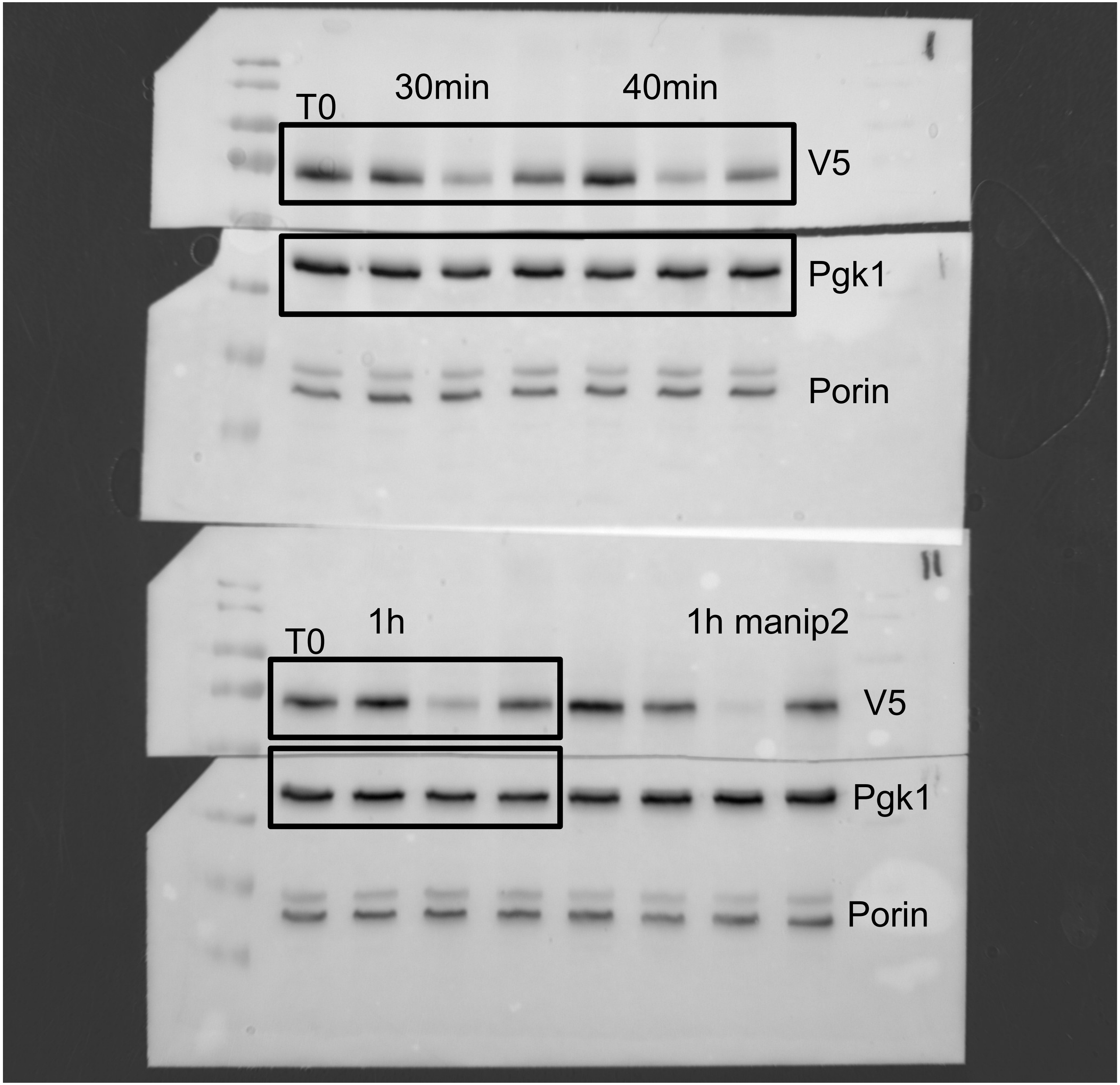

Figure 4B

Figure 5A : V5 blotting

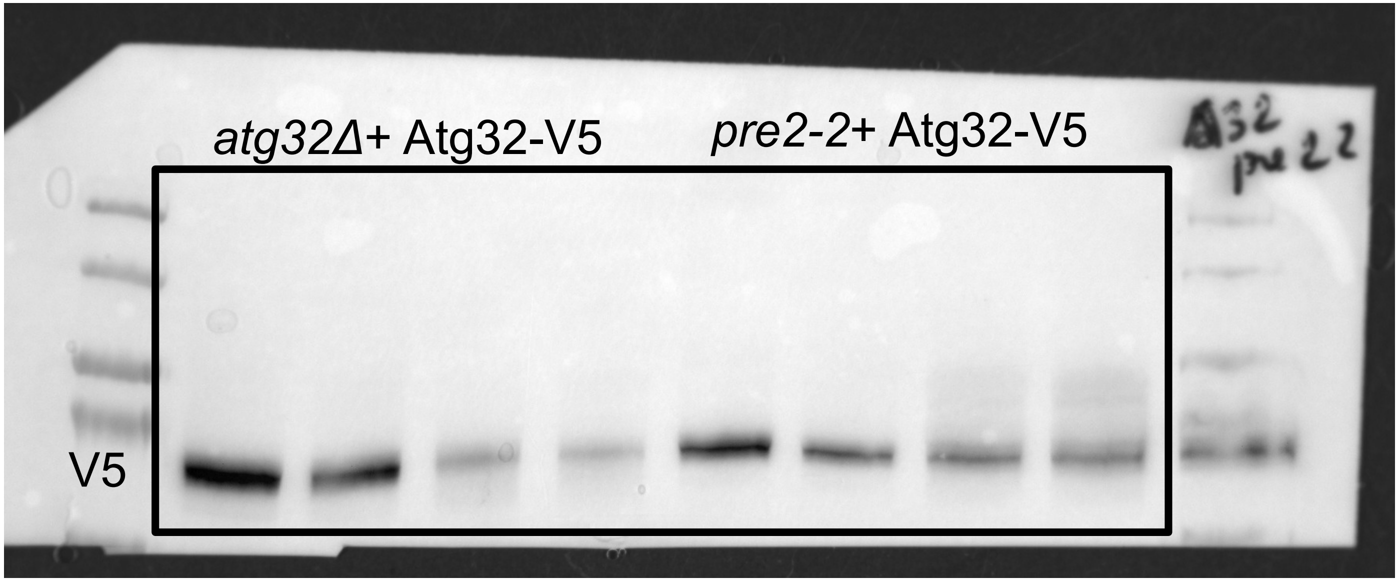

Figure 5A : Pgk1 blotting

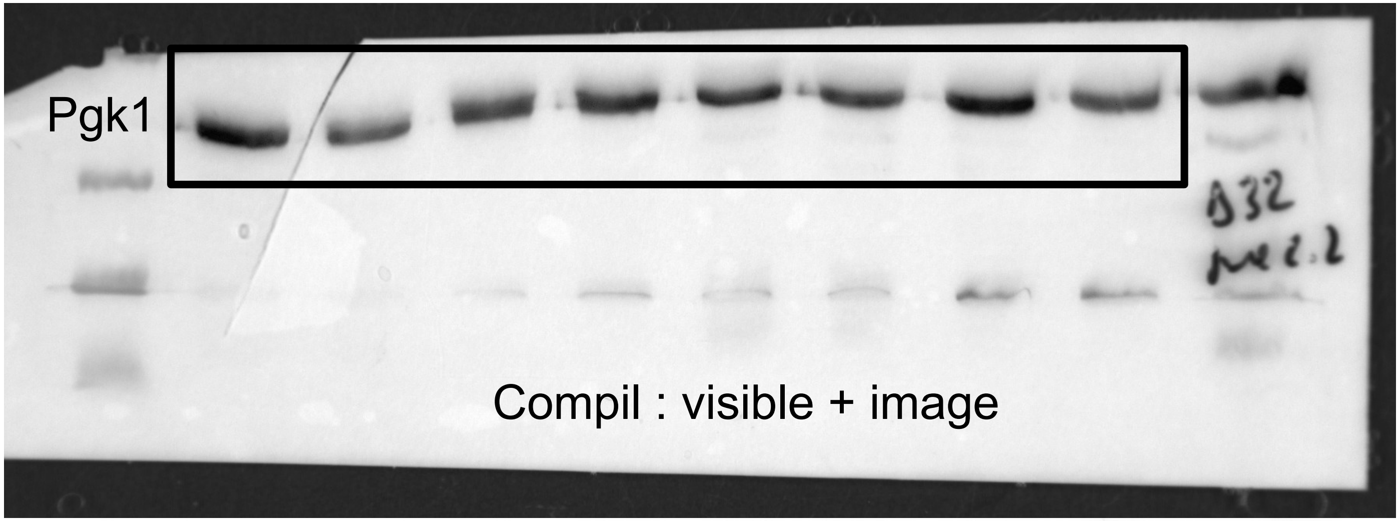

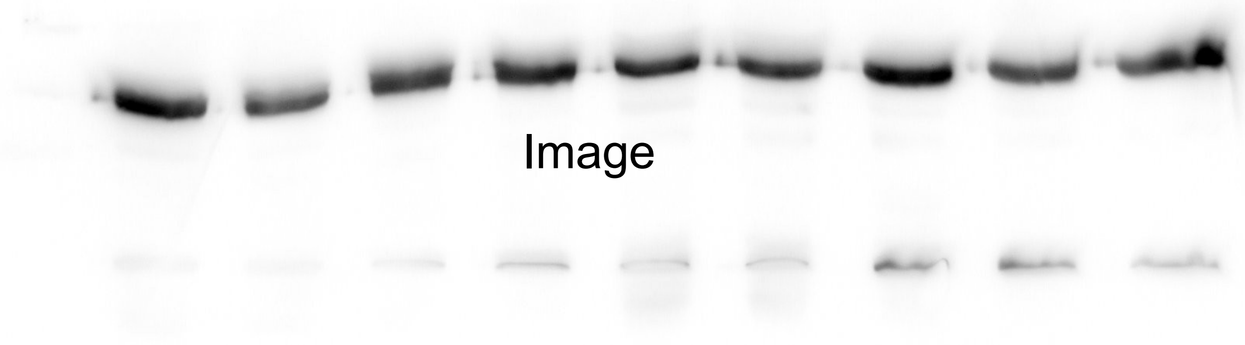

Figure 6A :
Blotting GFP

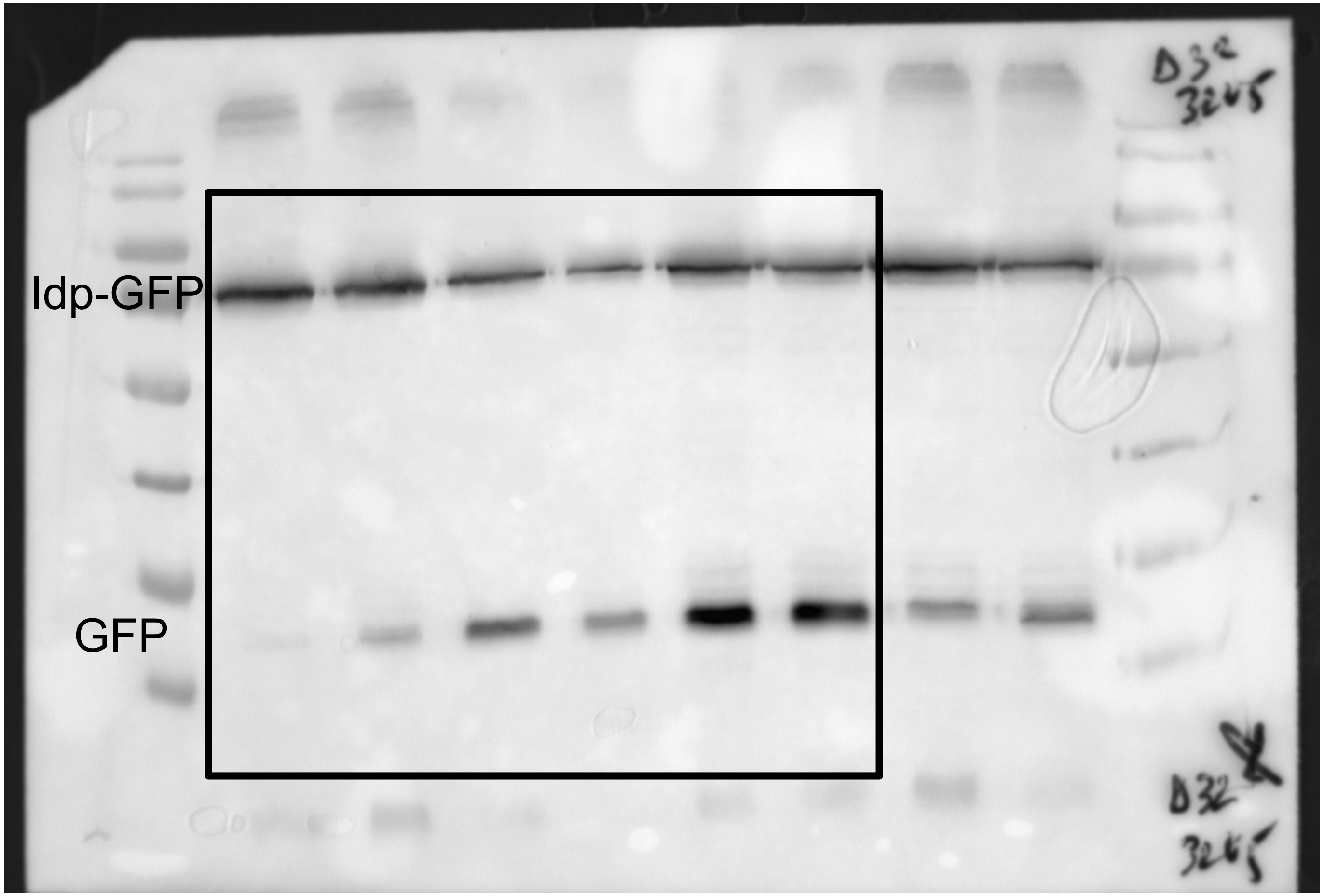

Figure 6A :
Blotting Pgk1

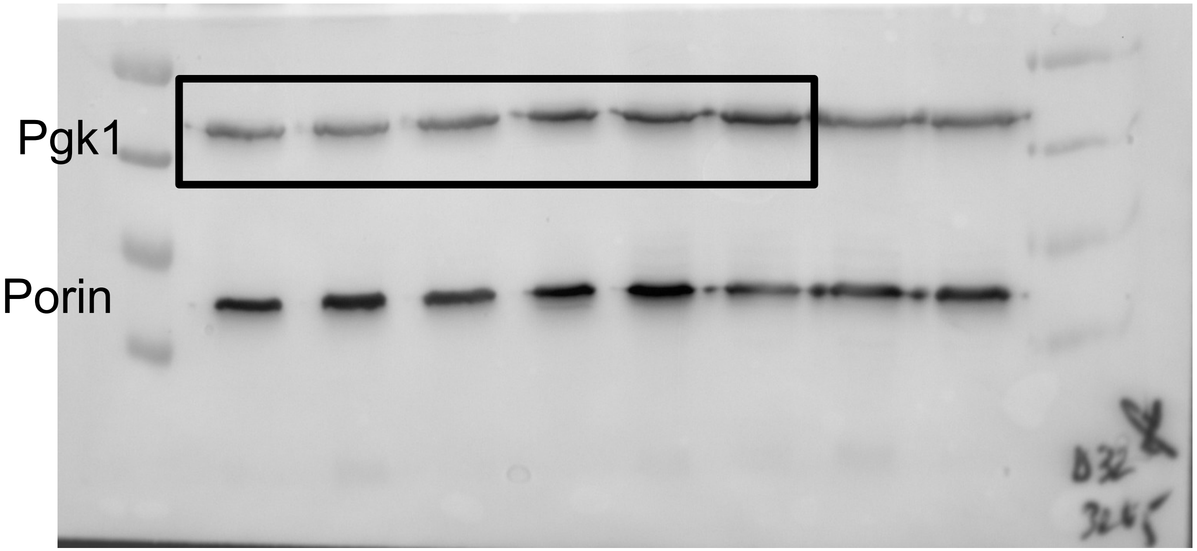

Figure 6B :
Blotting GFP

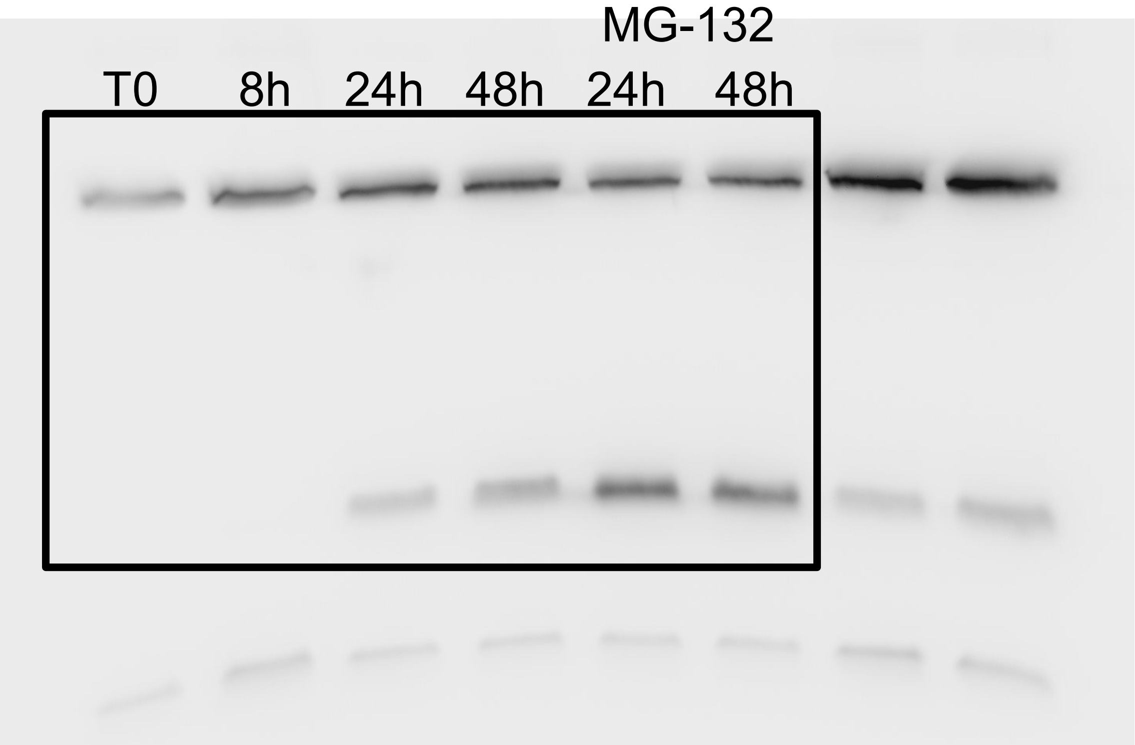

Figure 6B :
Blotting Pgk1

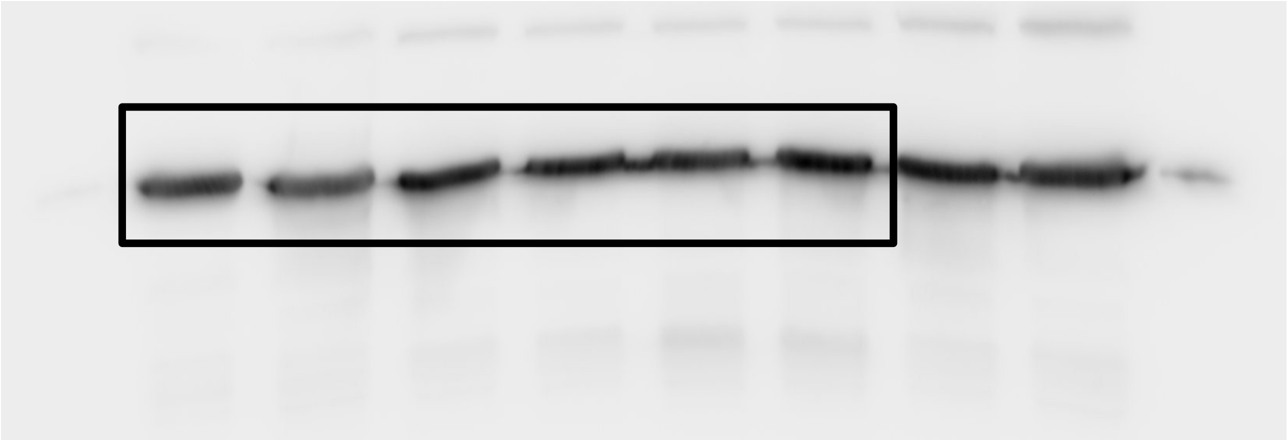

Figure 7A

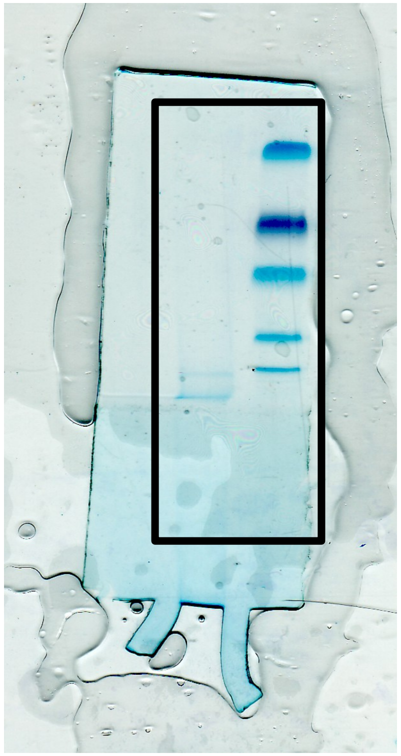

Figure 7B

histidine blotting

ubiquitin blotting

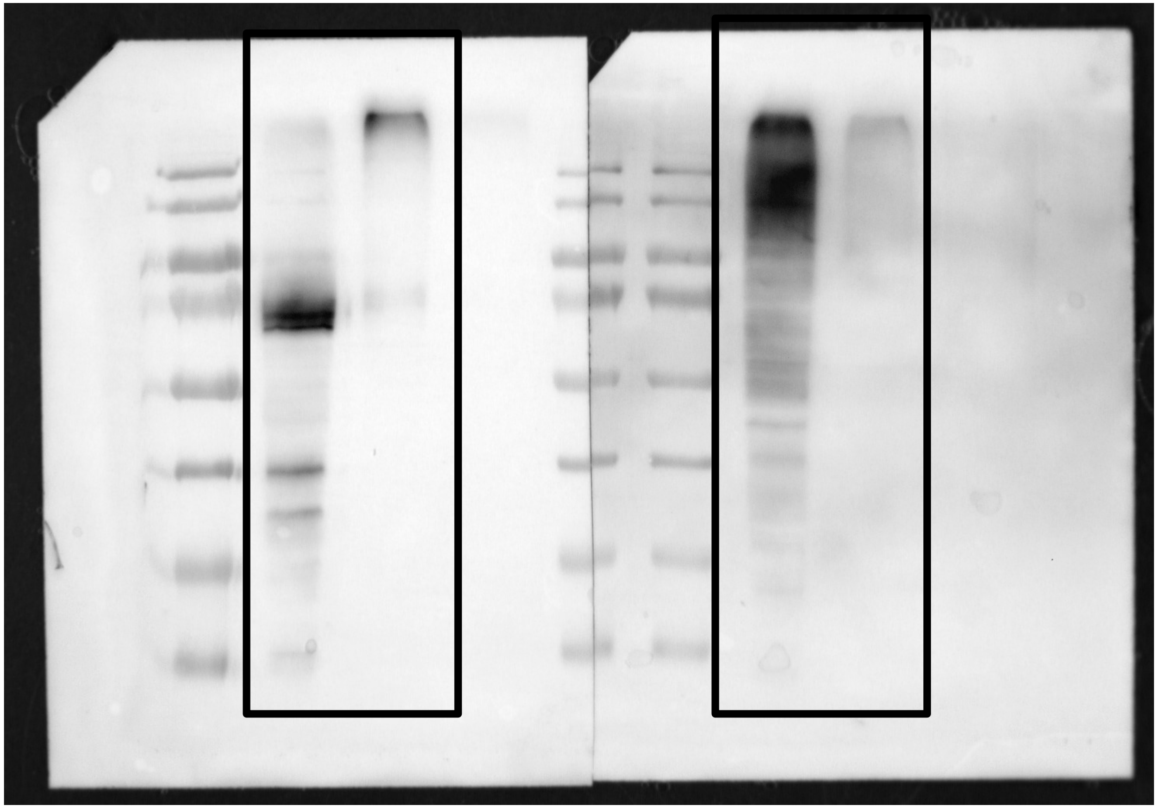

atg32Δ + Atg32-V5

Figure 8A : Blotting V5

Figure 8A : Blotting Pgk1

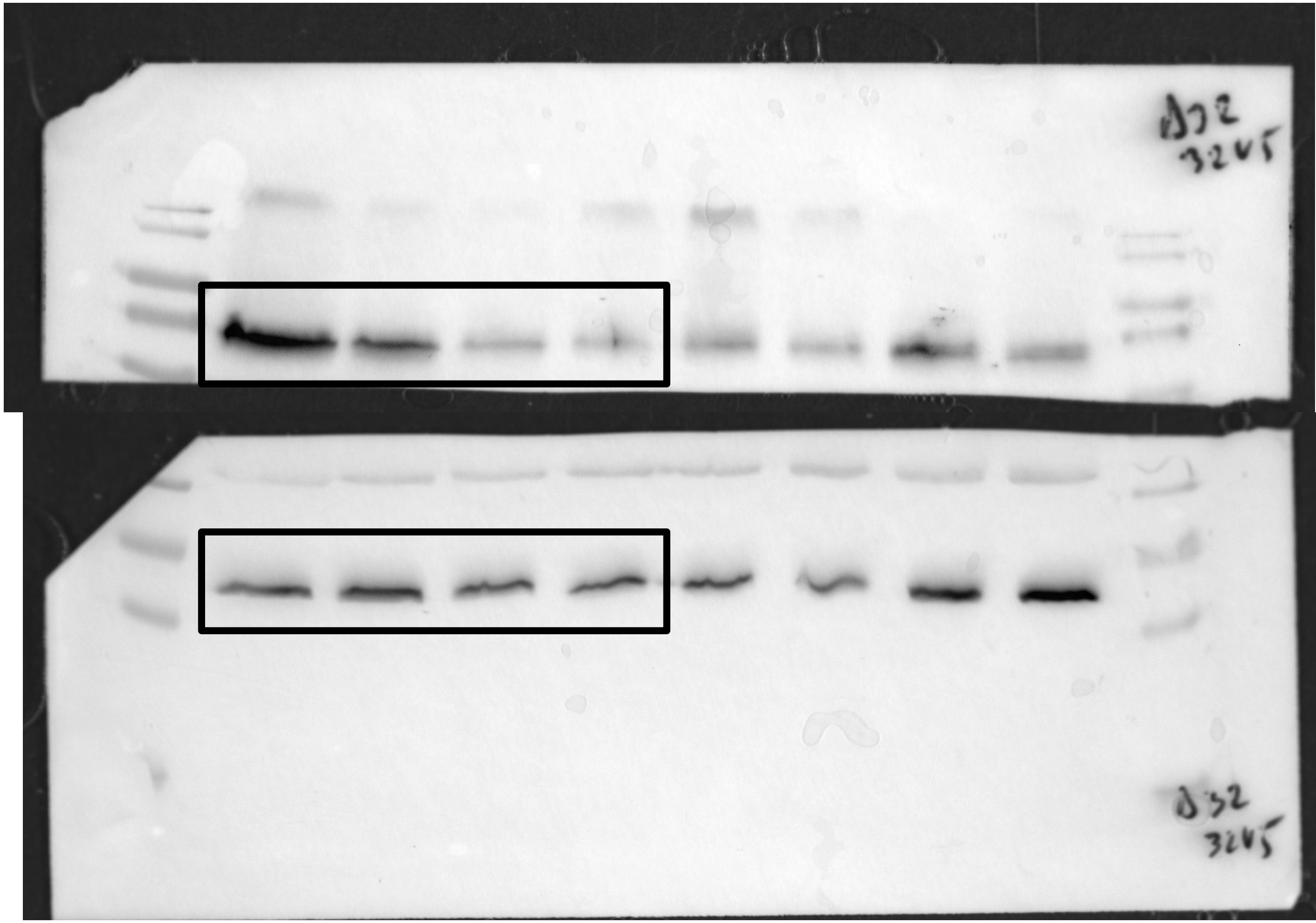

Lysine mutant

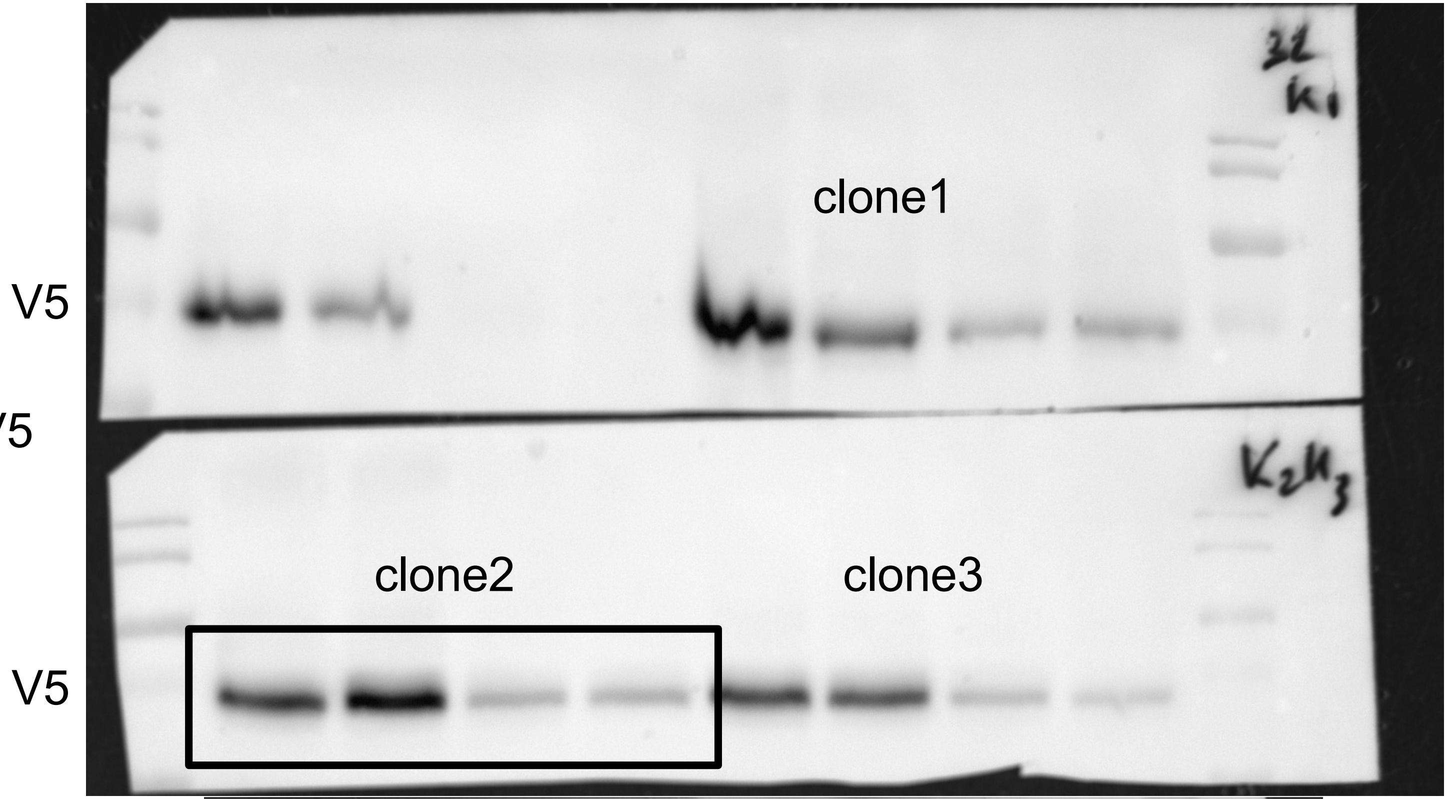

Figure 8A : Blotting V5

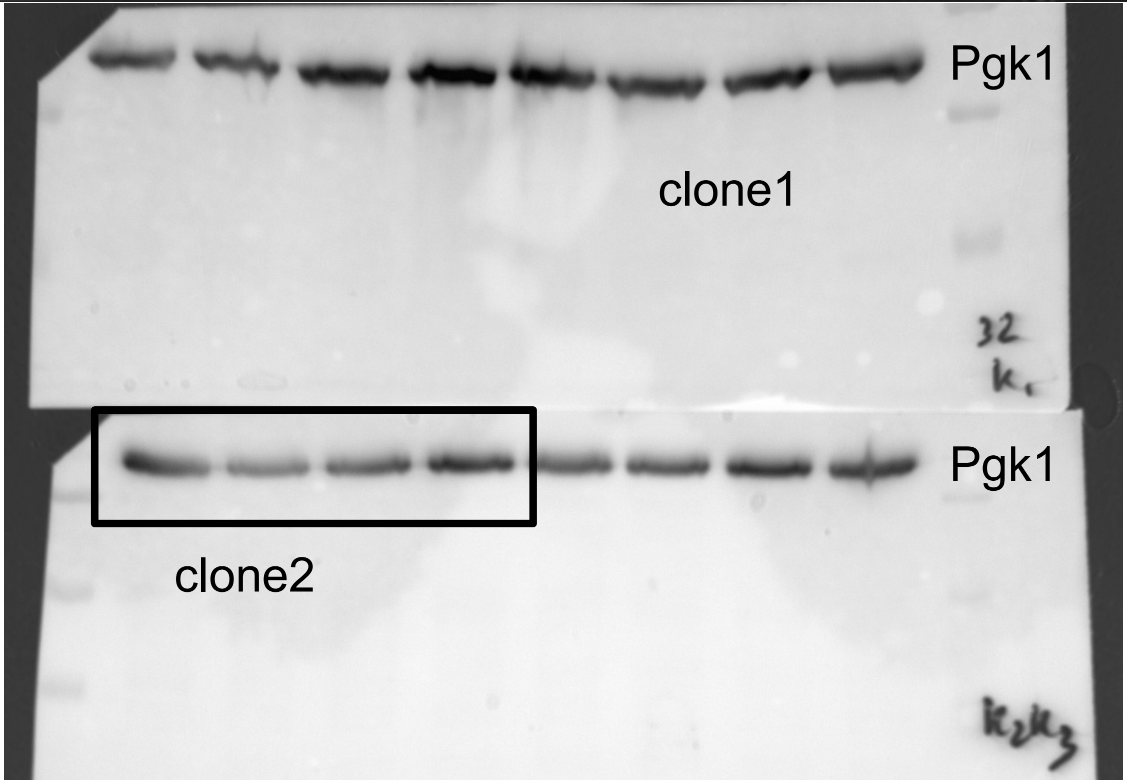

Figure 8A : Blotting Pgk1

atg32Δ + Atg32-V5

Figure 8B : Blotting V5

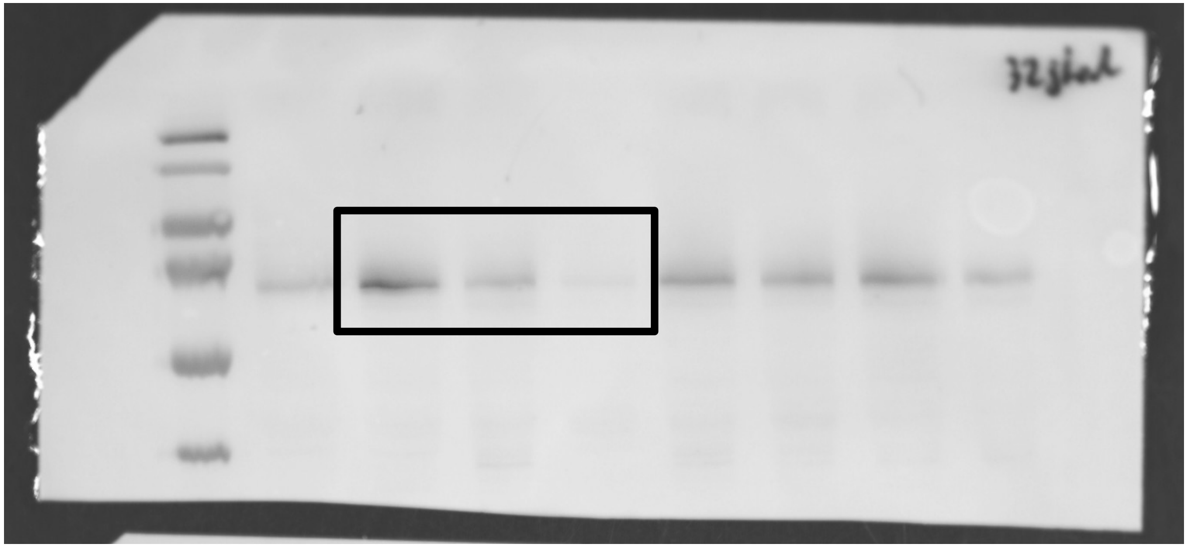

Figure 8B : Blotting Pgk1

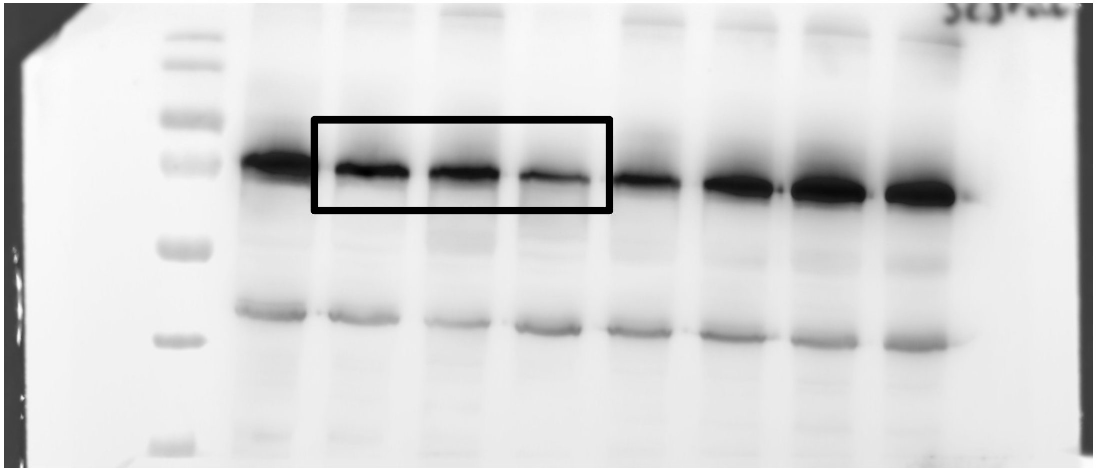

Atg32-AAAA

Figure 8B : Blotting V5

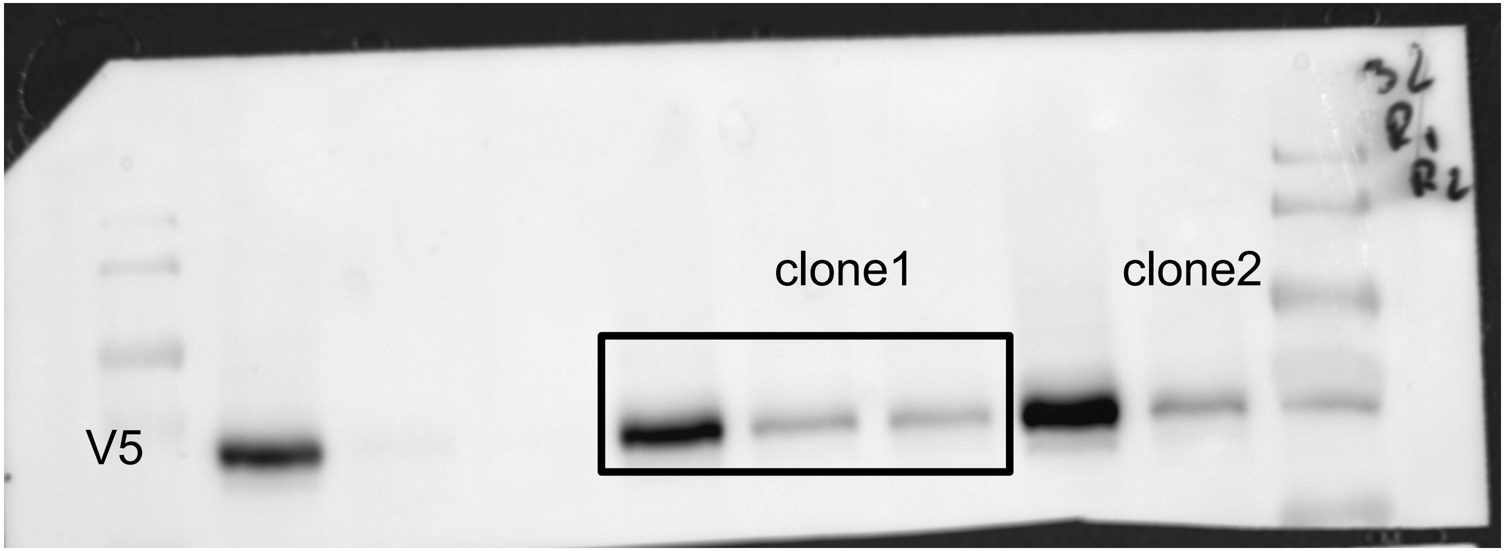

Figure 8B : Blotting Pgk1

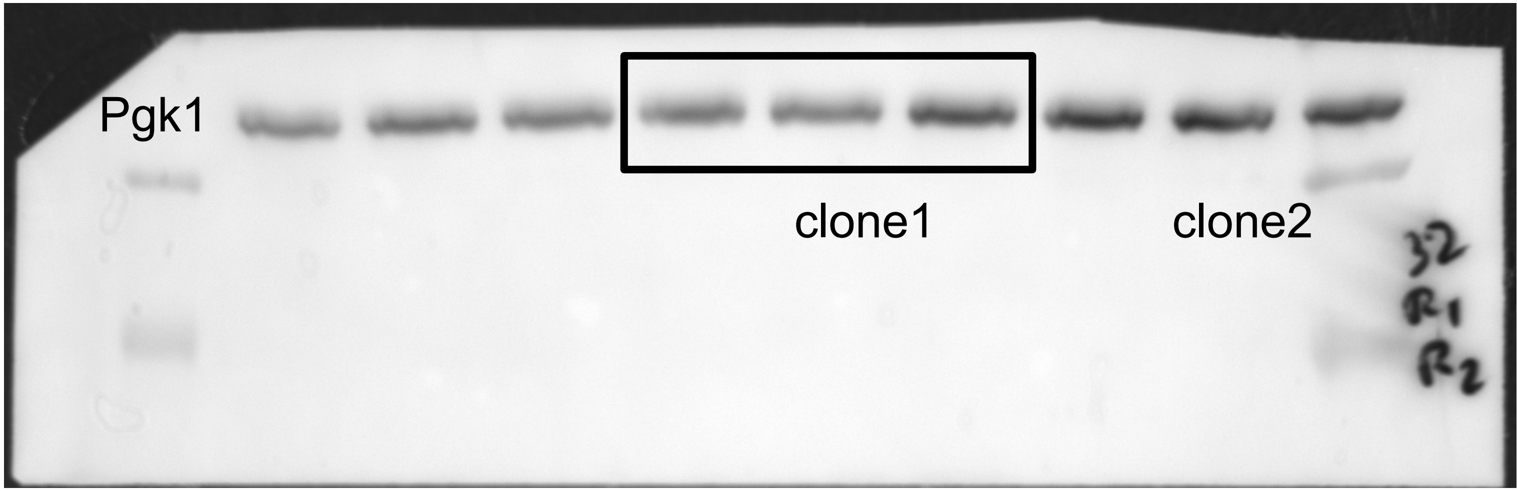

Figure 9A :
Blotting GFP

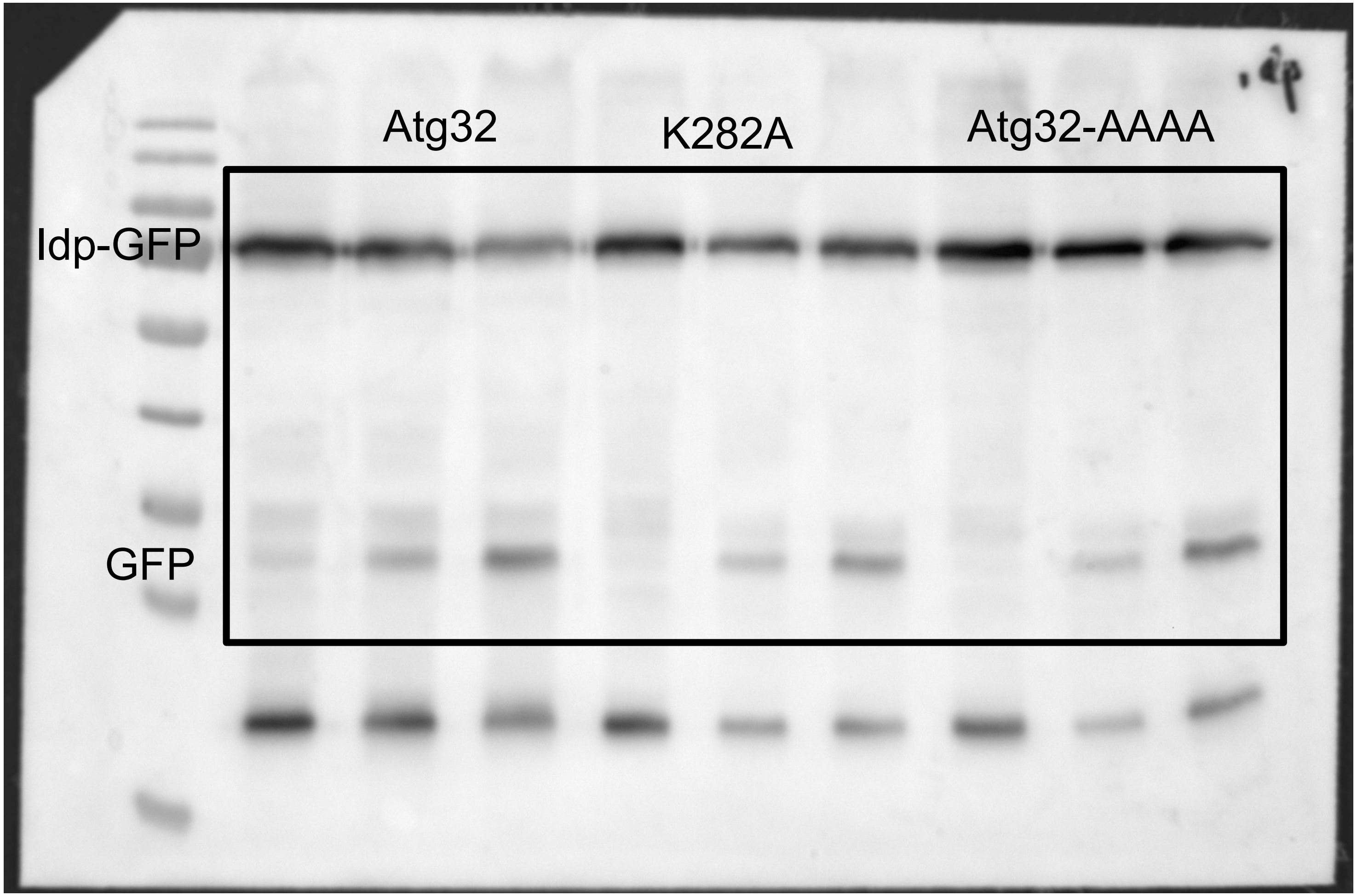

Figure 9A :
Blotting Pgk1

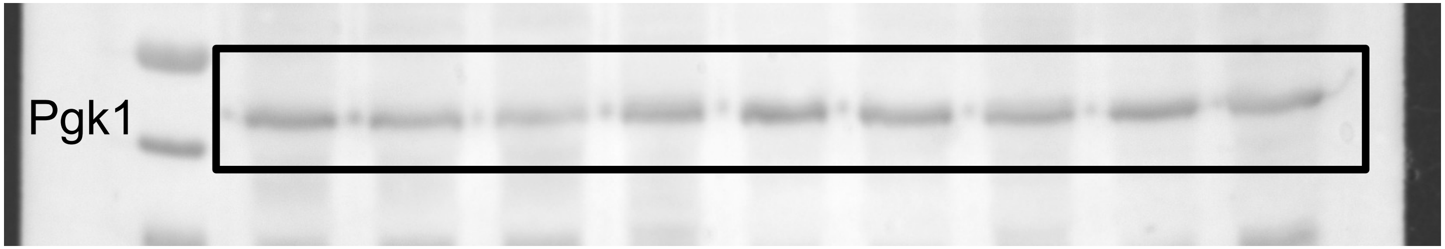

Figure S1A

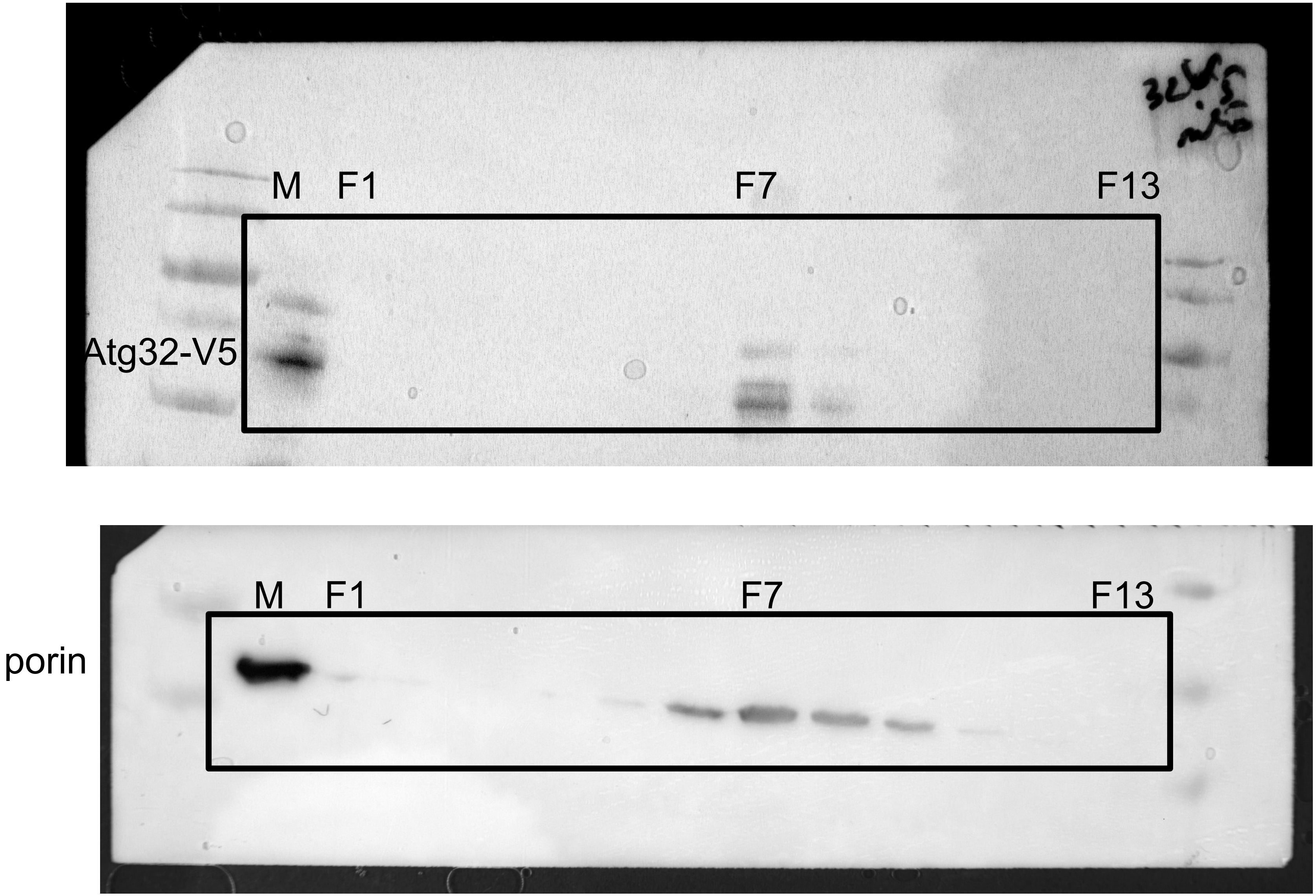

Figure S1B : GFP blotting

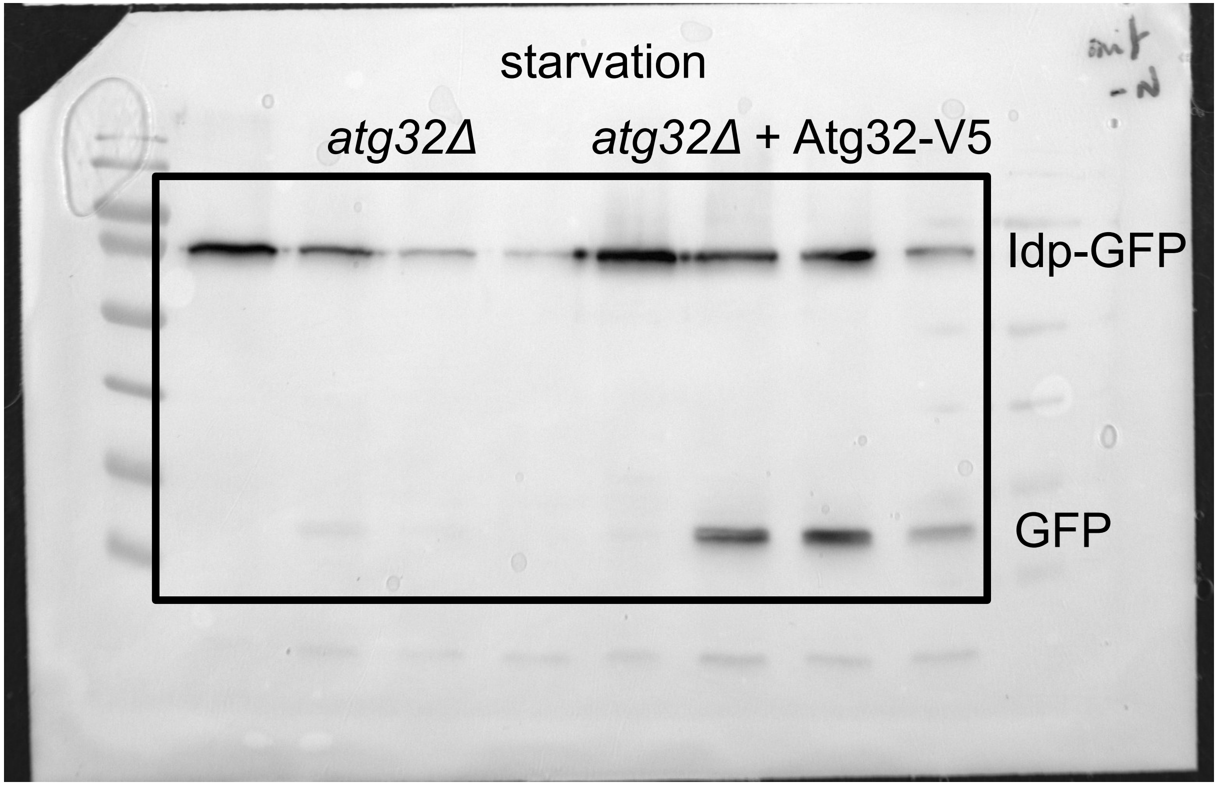

Figure S1B : Pgk1blotting

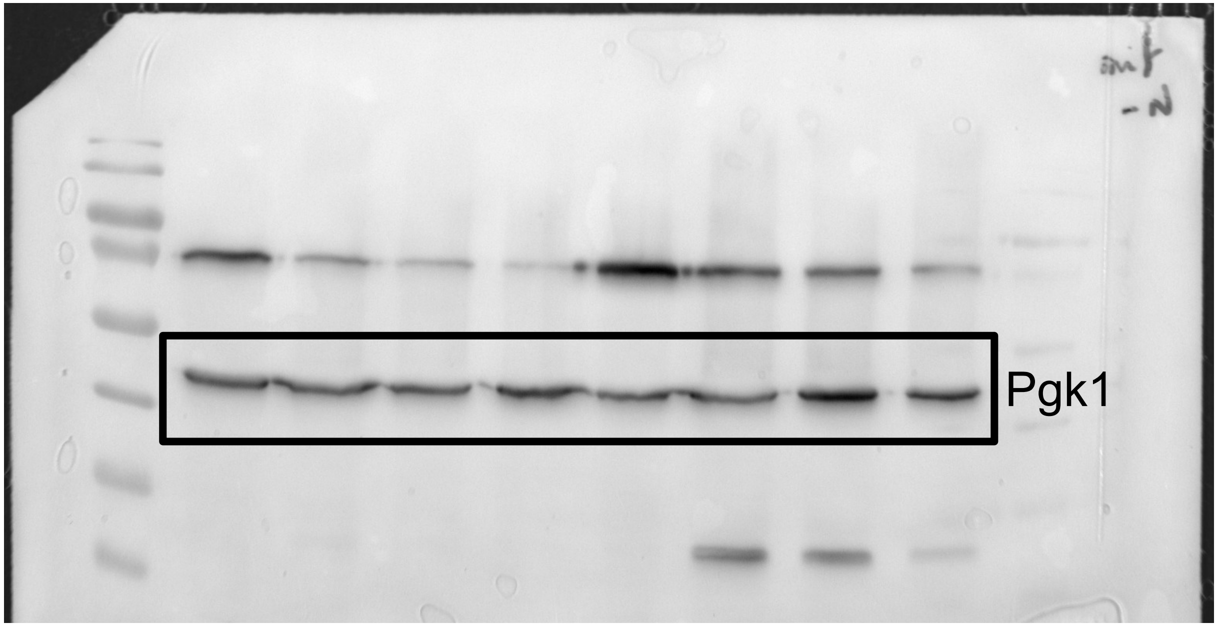

atg32Δ +Atg32-V5

Figure S2A : V5 blotting

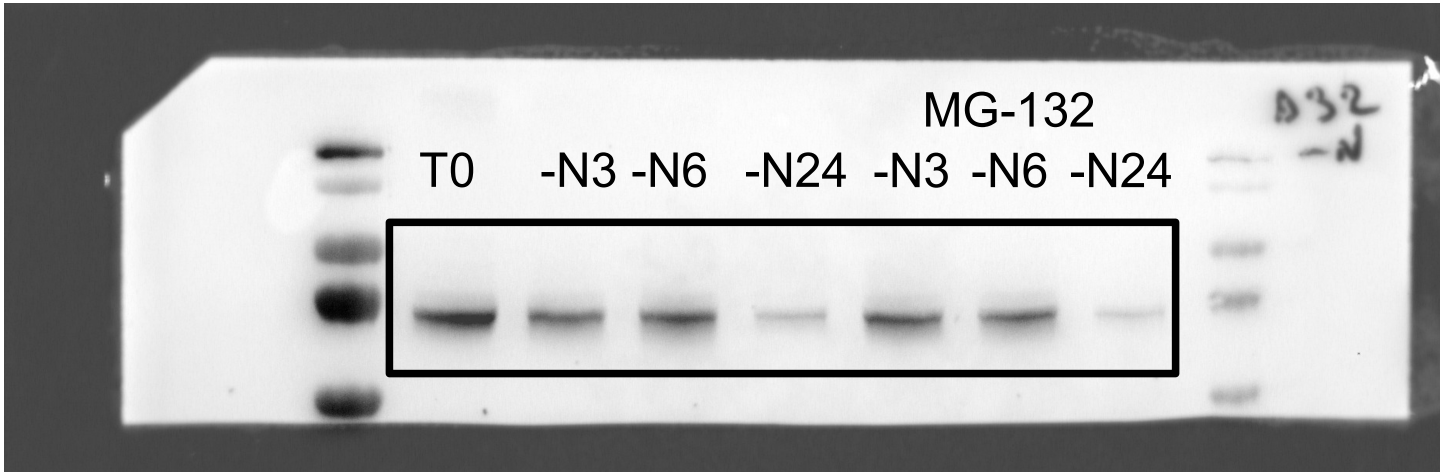

Figure S2A : Ubiquitin blotting

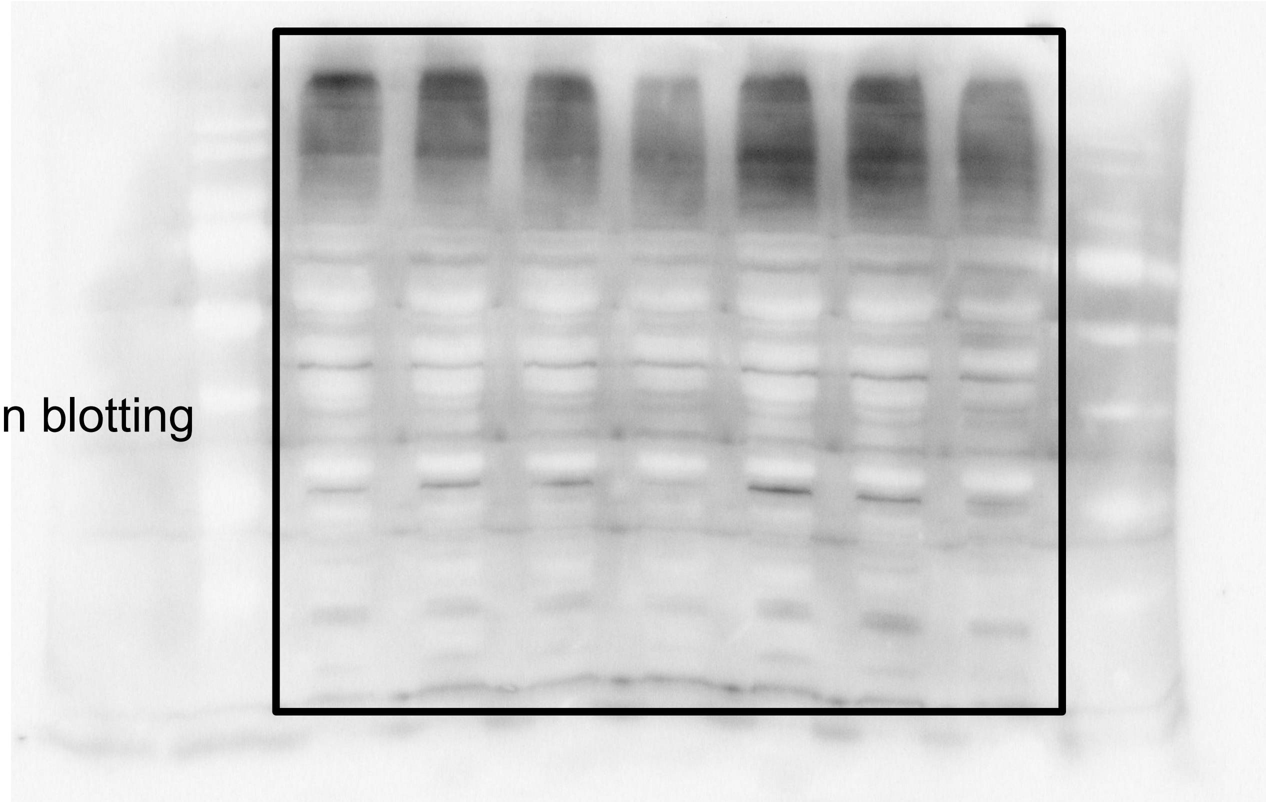

Figure S2A : Pgk1 blotting

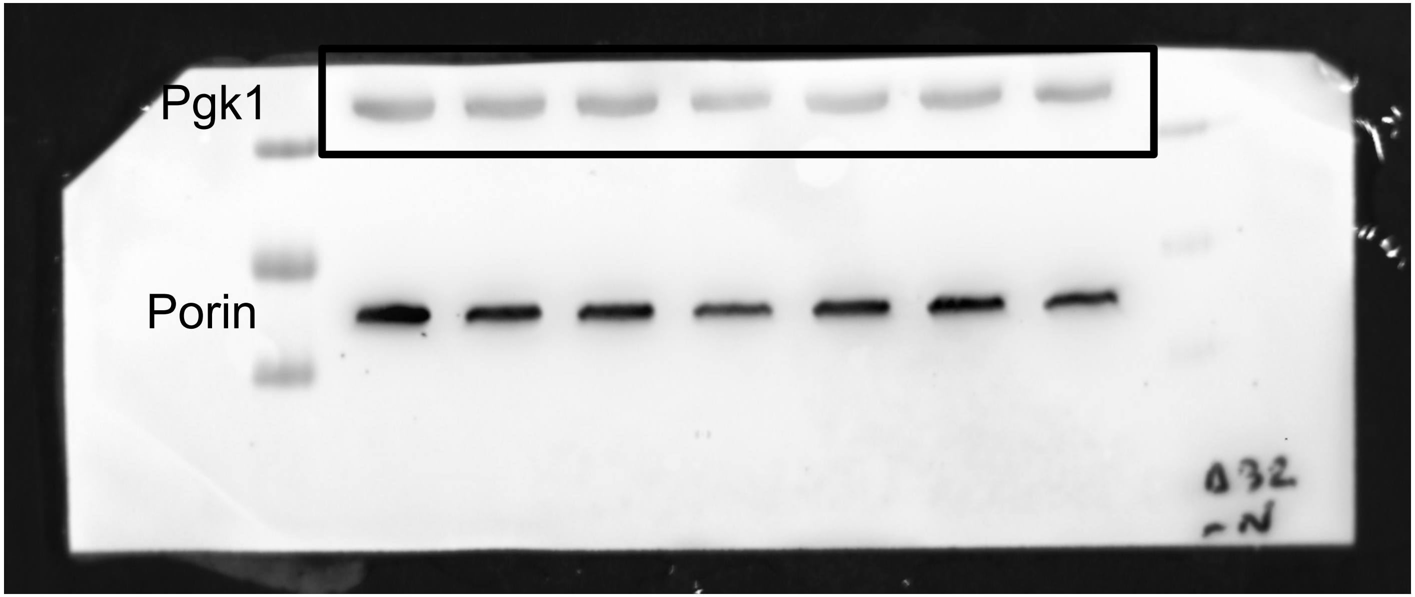

BY4742 +Atg32-V5

Figure S3 A : V5 blotting

Figure S3 A : Pgk1 blotting

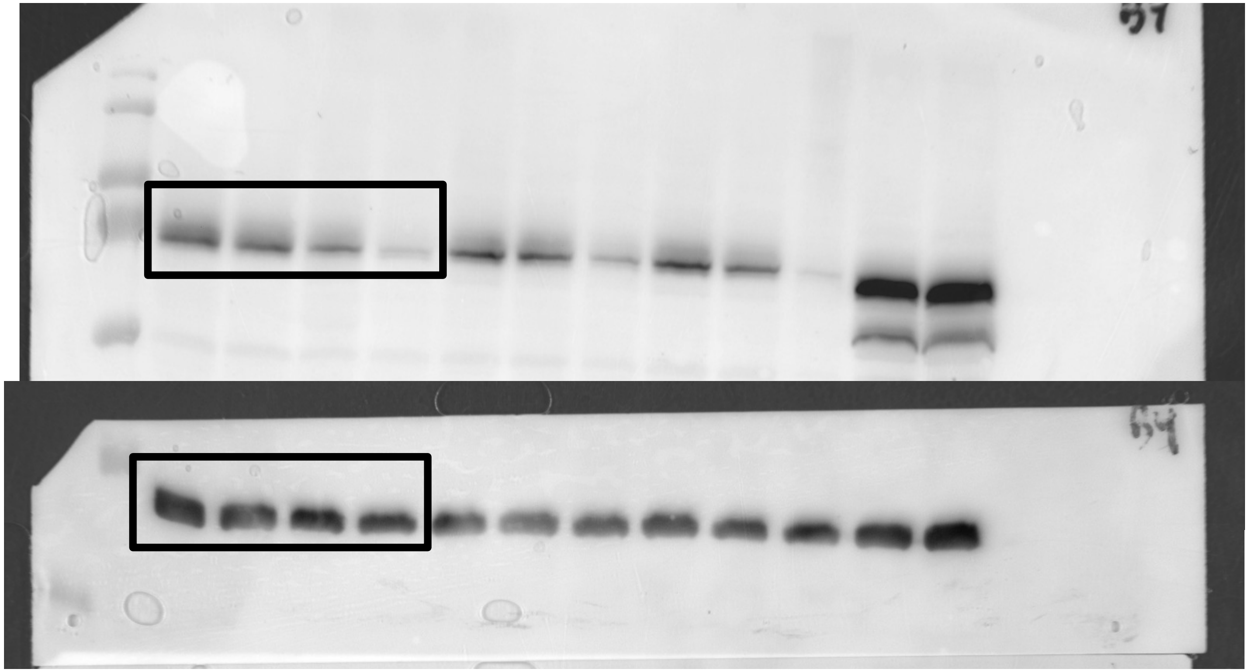

Figure S3 A : V5 blotting

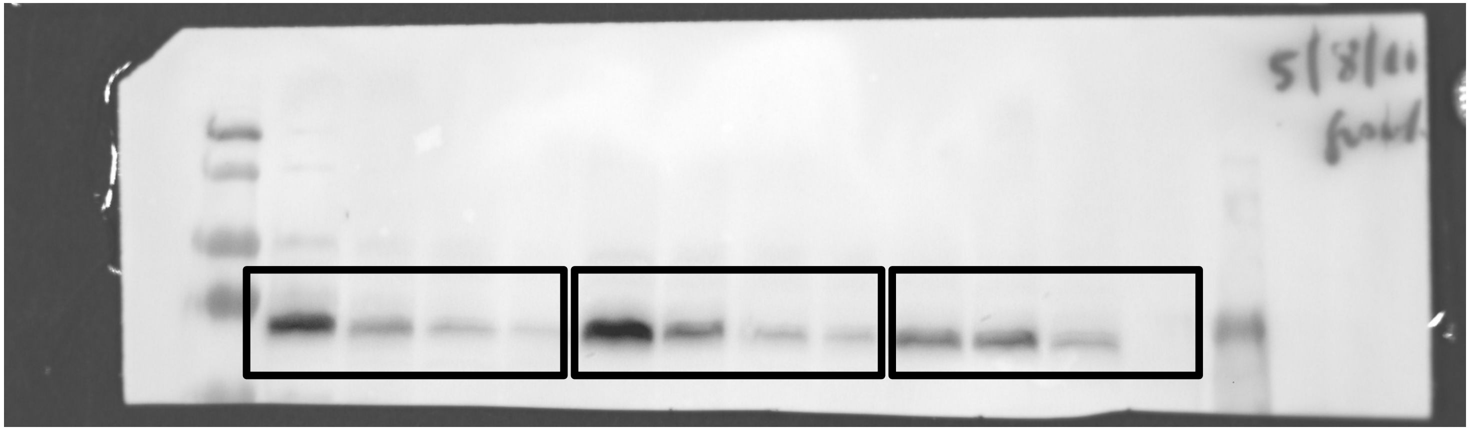

atg5Δ *atg8Δ* *atg11Δ*

Figure S3 A : Pgk1 blotting

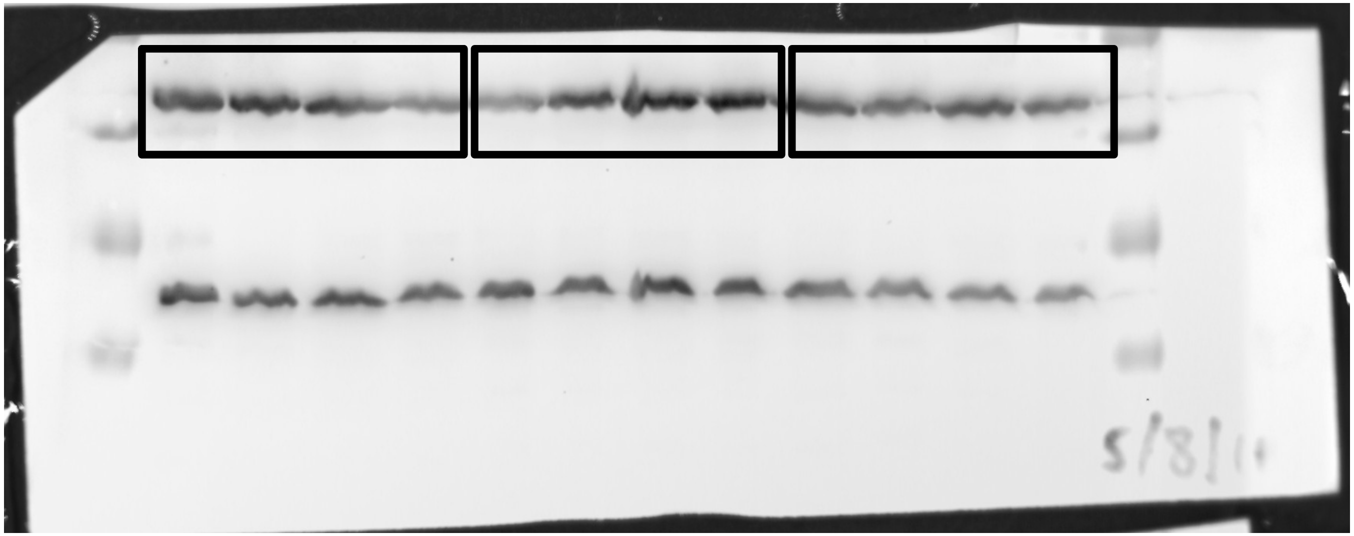

Figure S3 C : V5 blotting

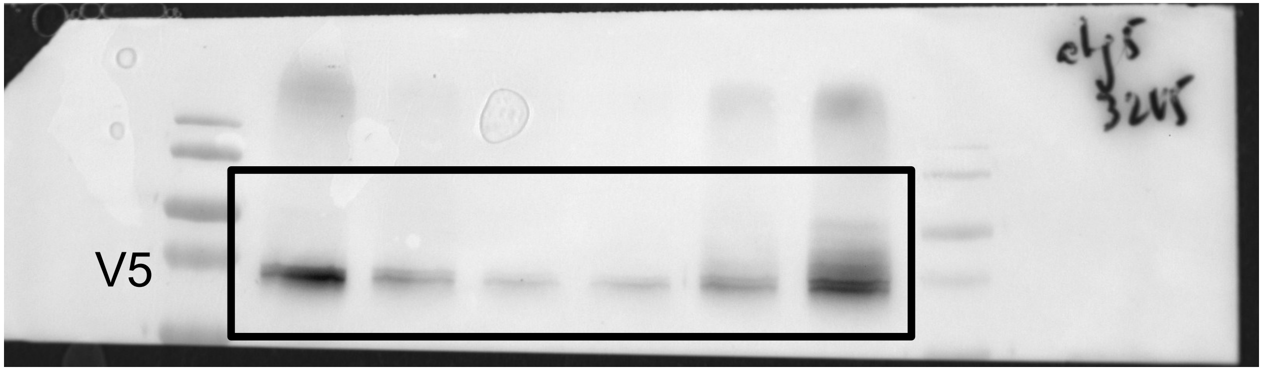

Figure S3 C : Pgk1 blotting

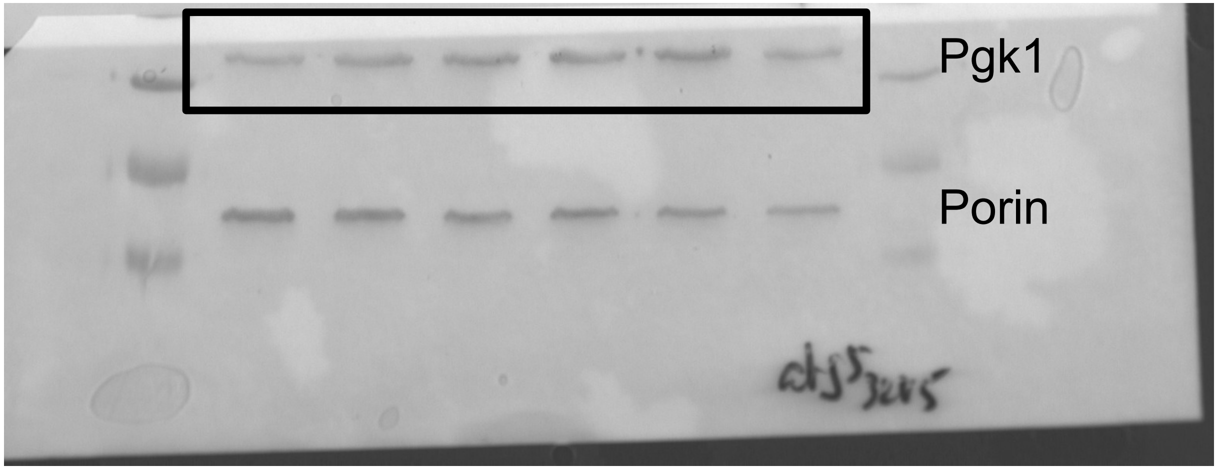

Figure S4A : V5 blotting

Figure S4A : Pgk1 blotting

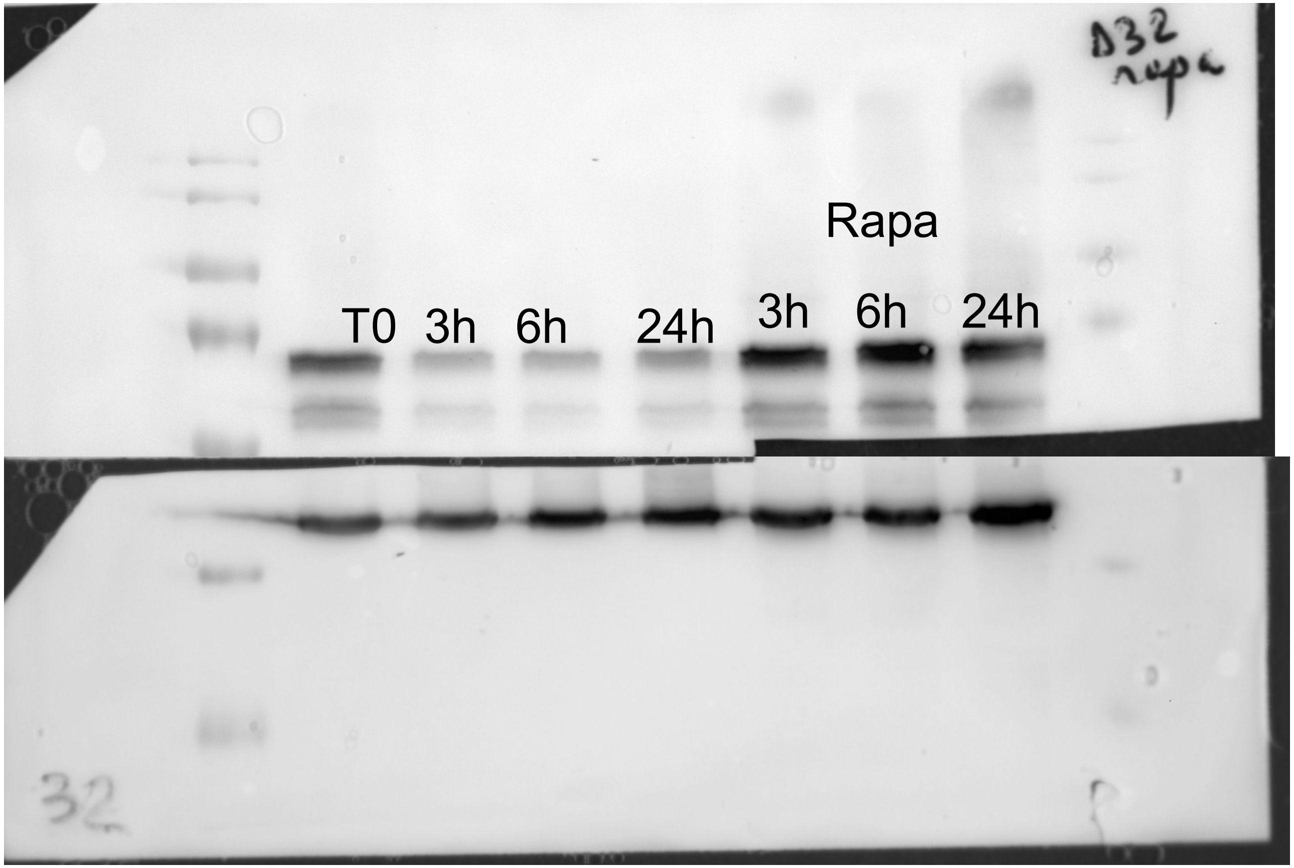

Figure S4B : V5 blotting

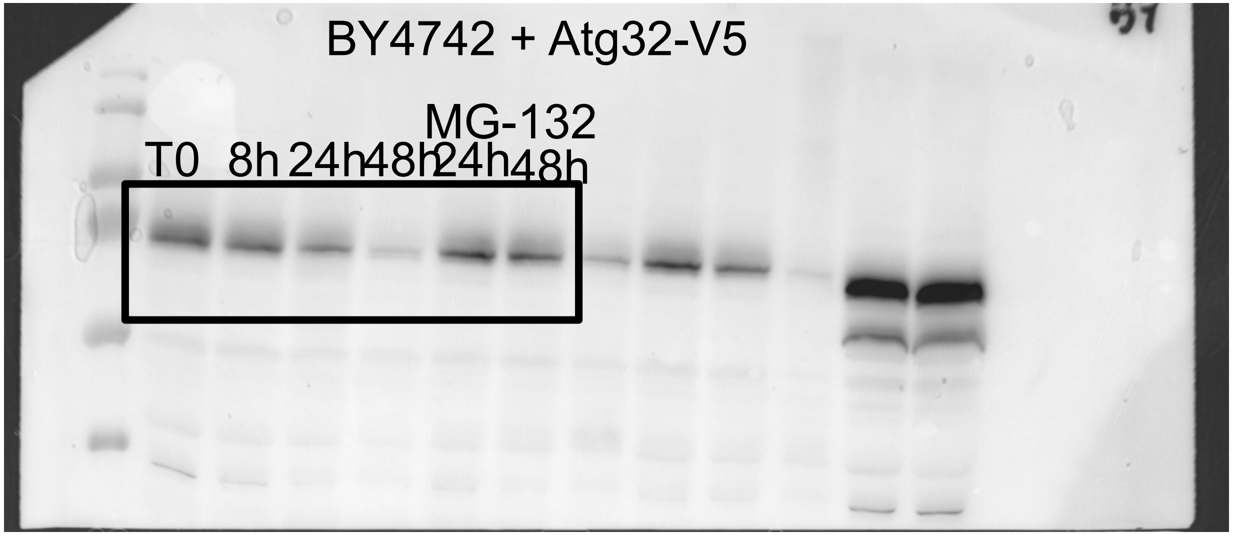

Figure S4B : Pgk1 blotting

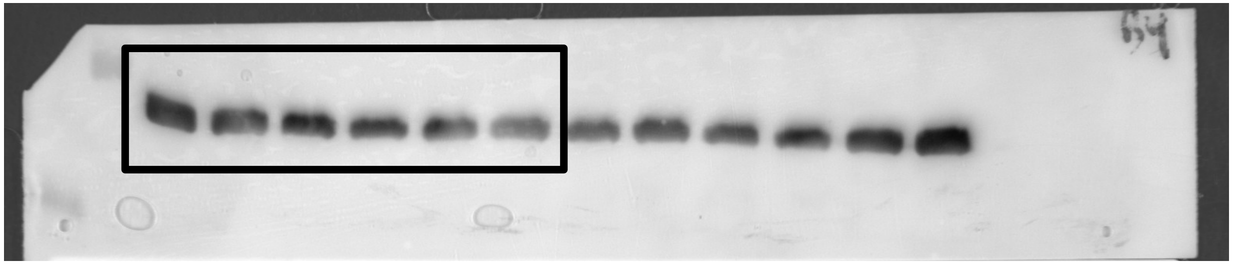

Figure S4D

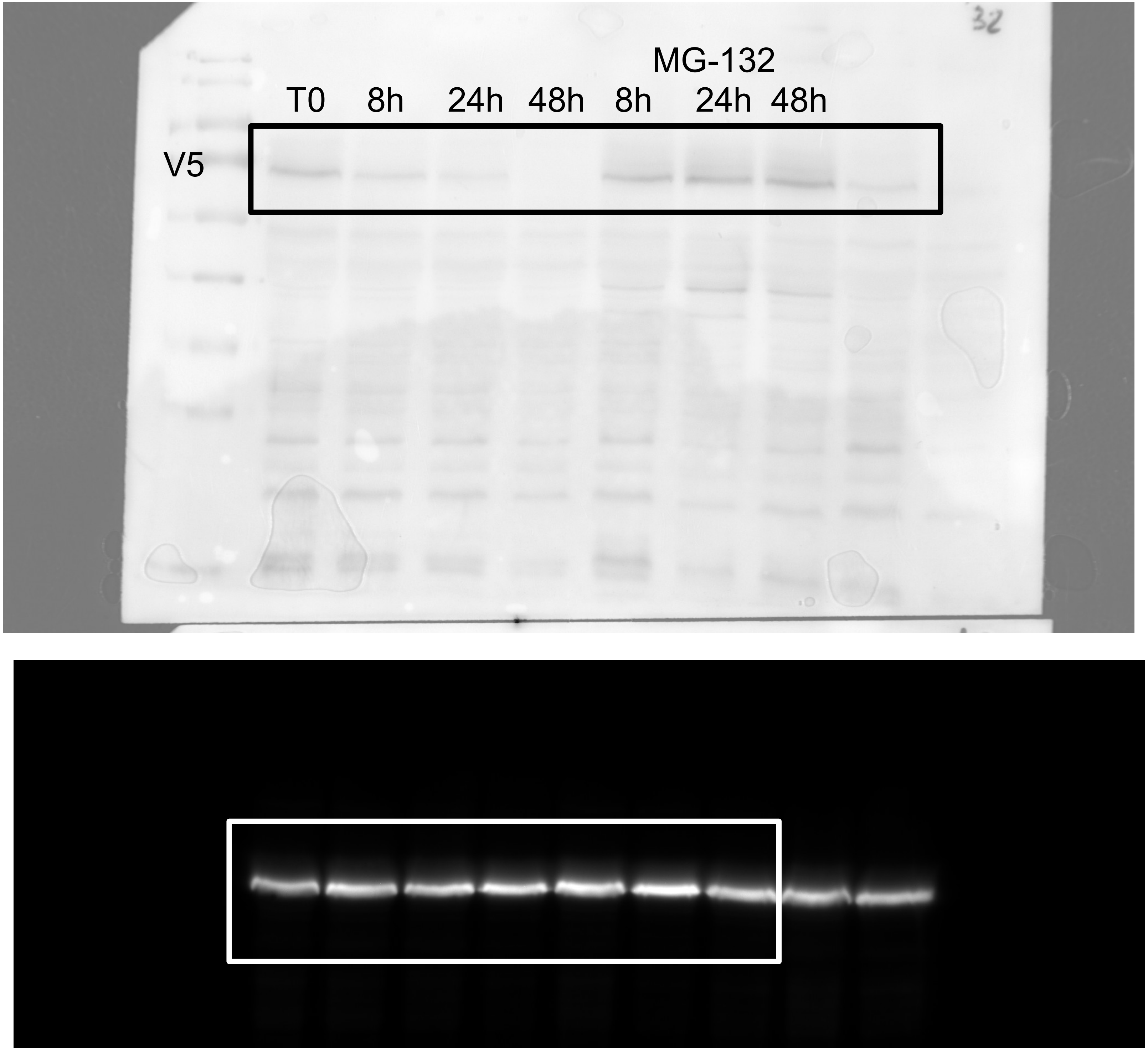

Pgk1

Figure S5B : V5 blotting

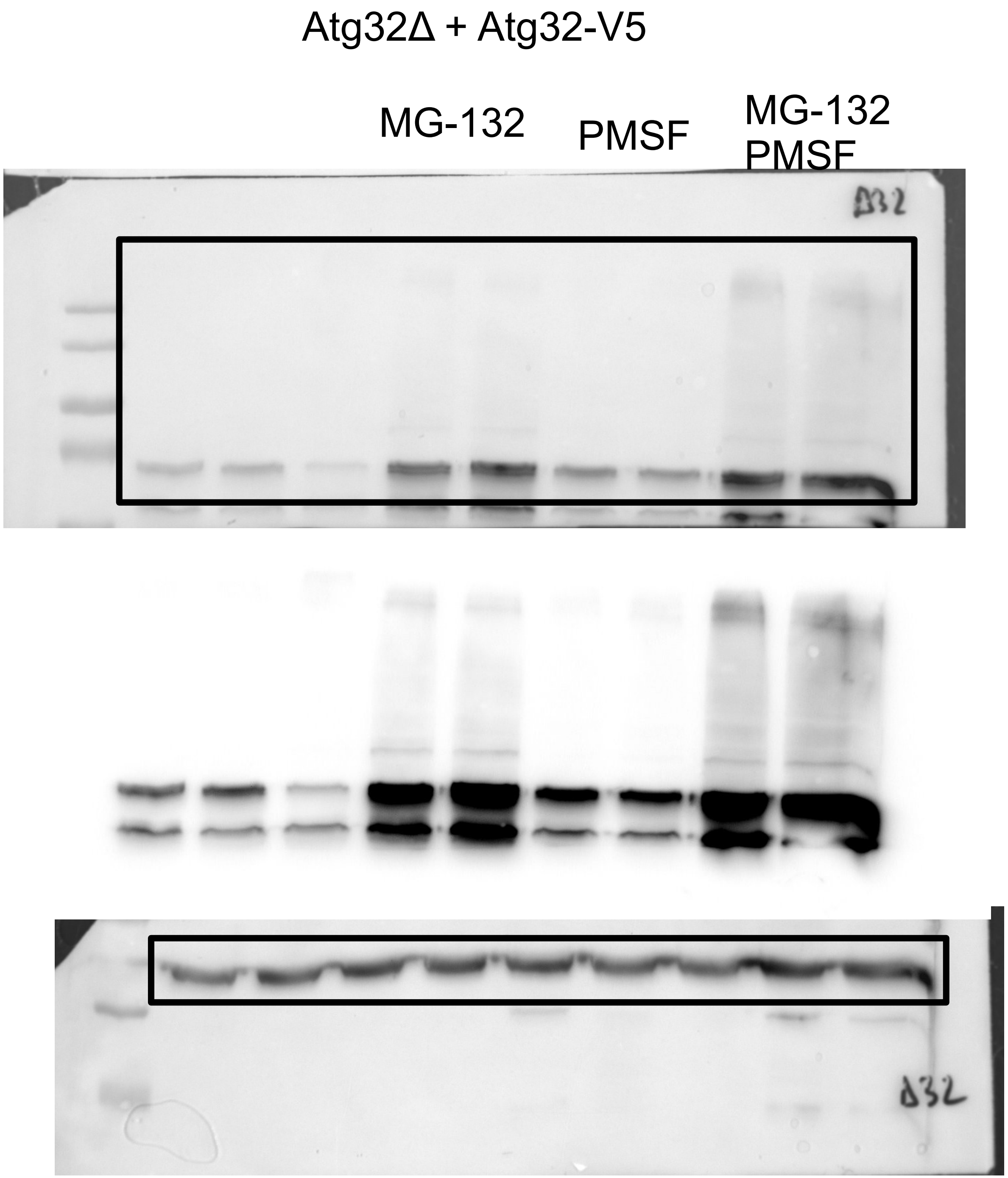

Figure S5B : Pgk1 blotting

Figure S6 : GFP blotting

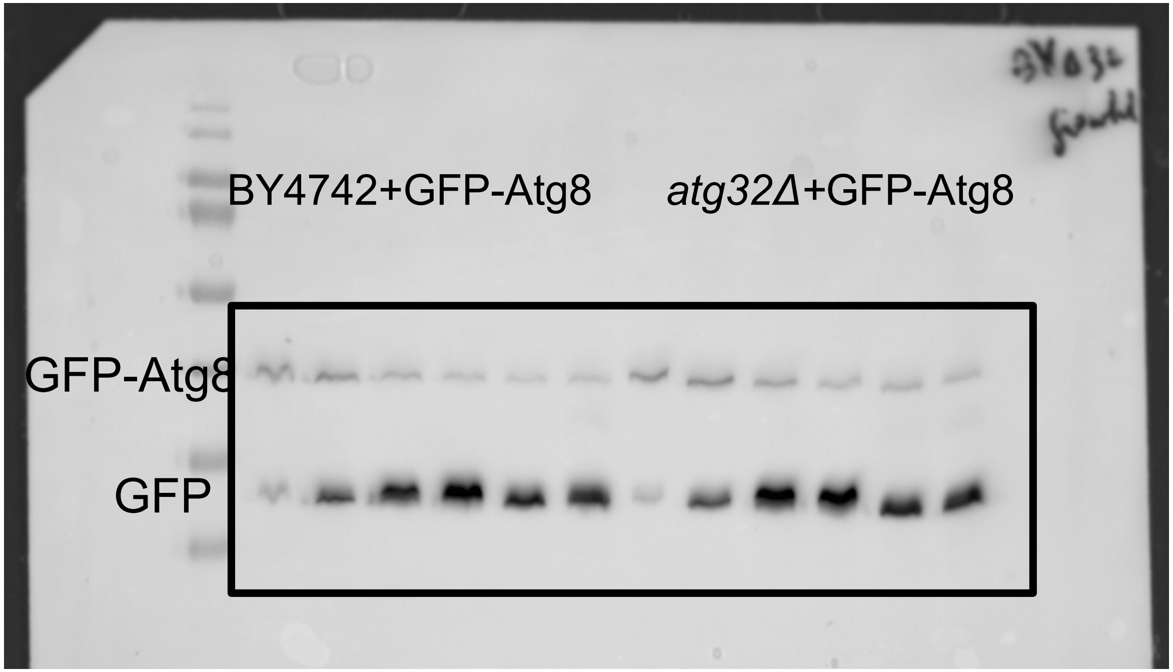

Figure S6 : Pgk1 blotting

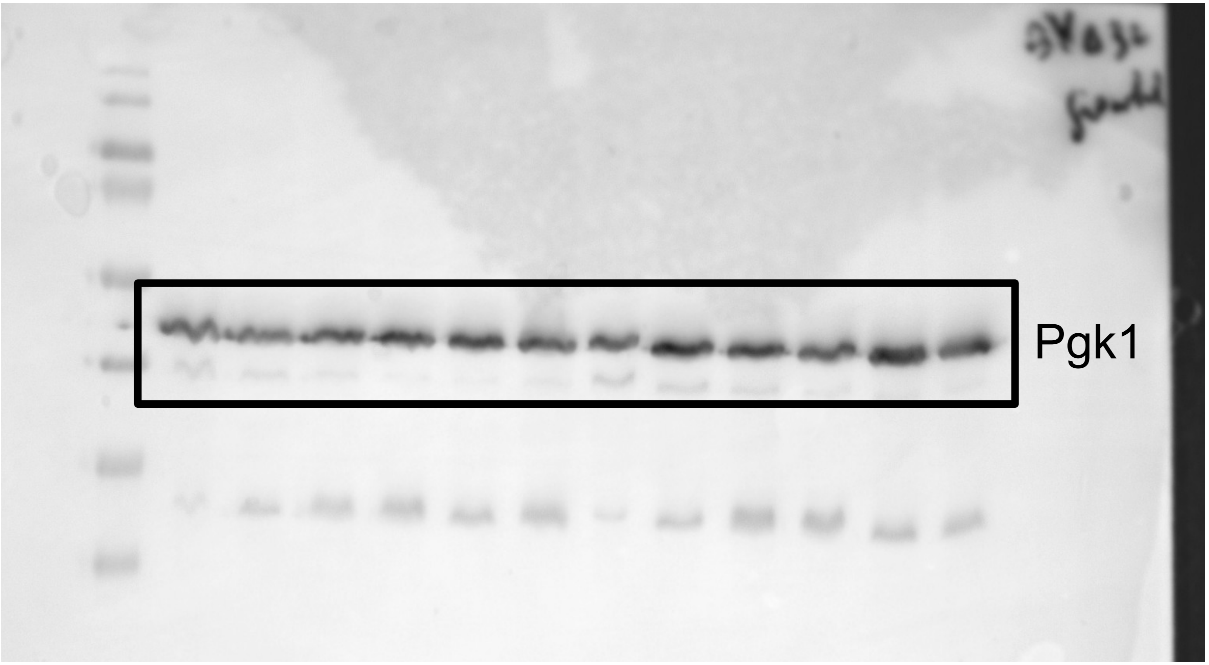

Figure S7

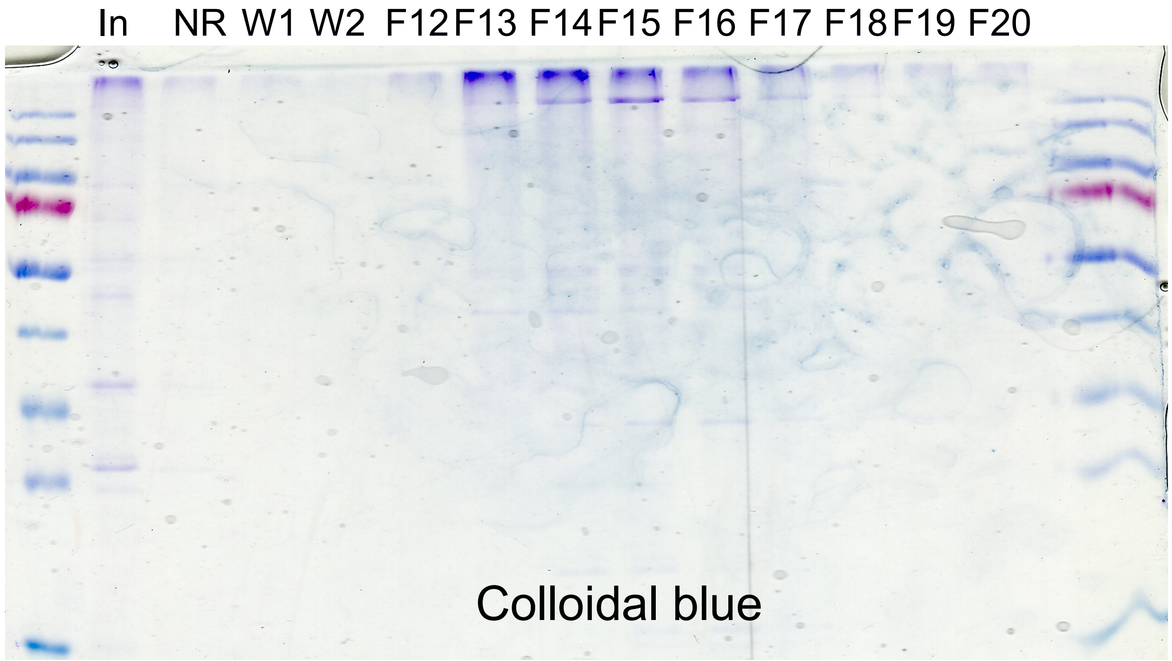
In NR W1 W2 F12 F13 F14 F15 F16 F17 F18 F19 F20
Colloidal blue

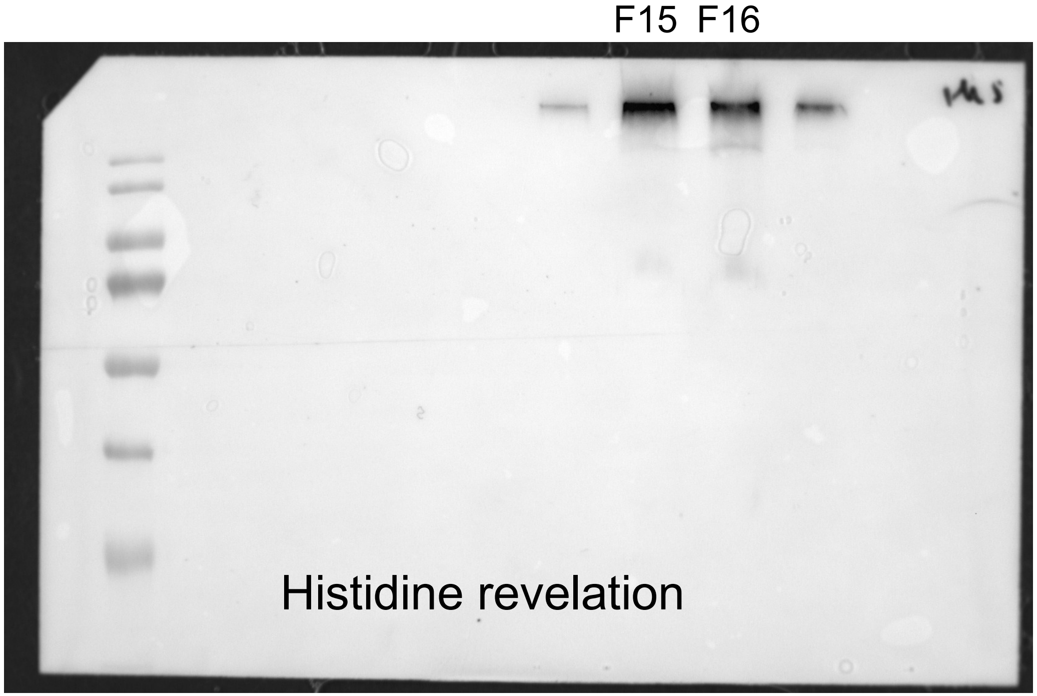
F15 F16
Histidine revelation

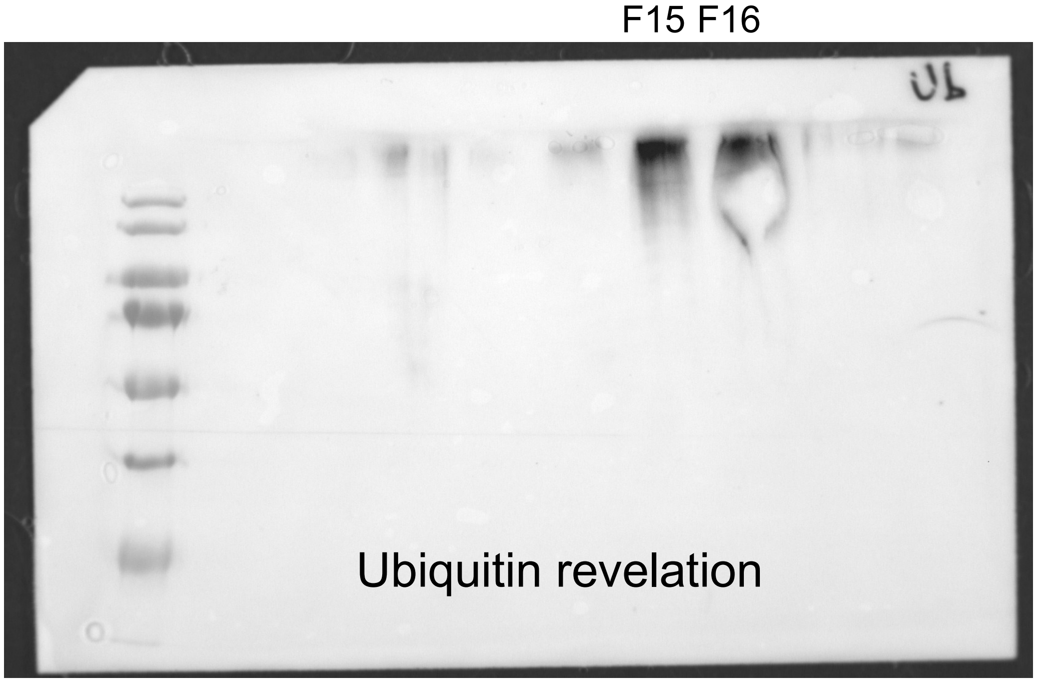
F15 F16
Ubiquitin revelation
