# Supplementary material for: The yeast mitophagy receptor Atg32 is ubiquitinated and degraded by the proteasome
Source: PLoS One. 2020 Dec 23;15(12):e0241576. doi: 10.1371/journal.pone.0241576 (PMC7757876; doi:10.1371/journal.pone.0241576)
Supplement: Attachment — Submitted filename: Responses to reviewers PONE-D-20-11526.pdf [file pone.0241576.s013.pdf]

PONE-D-20-11526
The yeast mitophagy receptor Atg32 is ubiquitinated and degraded by the proteasome

Answer to the Editor

We thank the reviewers and the editor for the time and effort they put into reviewing our manuscript and for their constructive comments. Nevertheless, we believe the expertise has been a bit severe and sometimes goes beyond the publishing policy of PLOS One. Following the first round of reviewing, we tried to give explanations and make modifications to address the reviewer's comments.

Please see below for the list of all figures included in the new manuscript with indicated changes compared to the original version.

Figure 1: We made no modifications.
Figure 2: We removed Figure 2C and 2D concerning starvation and replaced the HA-Atg32 blot during growth.
Figure 3: We removed data on starvation and included data on *pep4D* mutant (new Figure 3C and 3D).
Figure 4: We included graph (4C) concerning quantification of cycloheximide experiment.
Figure 5: We remove data on *pep4D* mutant (moved in Figure 3).
Figure 6: In Figure 6D, we removed data on starvation.
Figure 7: We arranged the figure and added immunodetection with anti-histidine antibodies.
Figure 8: We presented one representative blot for the mutants.
Figure 9: We made no modifications.
Figure 10 was deleted

Figure S1: Figure S1A is now S1B; we added data on purification of mitochondria and localization of Atg32-V5 (Fig. S1A).
Figure S2: We presented data on Atg32-V5 levels during starvation.
Figure S3: This figure corresponds to Figure S2 of the previous version.
Figure S4: This figure corresponds to Figure S3 of the previous version.
Figure S5: This figure corresponds to Figure S4 of the previous version. We added a high-exposure blot.
Figure S6: This figure corresponds to Figure S5 of the previous version.
Figure S6 of the previous version was removed.
Figure S7: We added this figure to better show the purification of Atg32-V5-6HIS (elution of the column, analysis of the different fractions by colloidal blue, and anti-HIS and anti-Ub before pooling adequate fractions where the results are shown in Figure 7).
Figure S8: Data on mass spectrometry corresponding to original Figure S7 of the previous version.

For better understanding, in several figures, we added more details/supplementary information. In the graphs, we added results of statistical analysis.

Please find below a point-to-point answer to the reviewer's comments.

We hope that this revised version of the manuscript, including modifications, will be acceptable for publication in PLOS One. We strongly believe that the data we present in this work are important for the scientific community interested in mitophagy and in the interactions between this mechanism involved in mitochondrial quality control and the ubiquitin proteasome system.

Dr. Nadine Camougrand

Reviewer's Responses to Questions

Comments to the Author

1. Is the manuscript technically sound, and does the data support the conclusions?

The manuscript must describe a technically sound piece of scientific research with data that supports the conclusions. Experiments must have been conducted rigorously, with appropriate controls, replication, and sample sizes. The conclusions must be drawn appropriately based on the data presented.

Reviewer #1: No

Reviewer #2: No

To our knowledge, no specific antibodies against the Atg32 protein are available so far. We agree that labeling the protein at the locus would have been the ideal construct for every study aiming to examine stability and expression of the Atg32 protein. However, we believe that the construct we used in our study to express Atg32 protein with a V5-6HIS label at its C-terminus is a good compromise. We assert that the *ATG32* gene in our construct has been put under the control of its own promoter. This type of strategy has been used in many published works. Moreover, we did not limit ourselves to testing a single construct; we also used the plasmid provided by Dr. Klionsky (Michigan University, USA) which allows the expression of the N-terminal labeled Atg32 protein with the HA tag. We obtained the same results using both plasmids.

Also, we reversed the phenotype of *atg32Δ* mutant expressing Atg32-V5 plasmid; the protein is localized into mitochondria (Fig 1A), and we also included figure S1A to demonstrate mitochondrial localization of Atg32-V5 using purified mitochondria isolated from *atg32Δ* mutant cells expressing Atg32-V5 harvested in the mid-exponential phase. Additionally, we demonstrated that Atg32-V5 restored mitophagy in *atg32Δ* mutant cells to the level similar in wild-type cells in stationary phase (Fig 1B) as well upon starvation (Fig S1B).

Further, to confirm the significance of our findings, we used a proteasome mutant *pre2-2* viable in our experimental setup as well as a vacuolar protease mutant *pep4Δ*. We believe our results provided clear evidence that the described changes in stability/turnover of Atg32 protein are not due to unspecified/long-term stress as suggested by the reviewer. In our study, we focused mainly on the two most physiological conditions for inducing mitophagy: stationary growth phase and nitrogen

deficiency, rather than the use of rapamycin, which is easier to use but causes more stress to the cells.

We believe that we used proper control in each experiment. All experiments were performed independently several times, as indicated for each experiment. The sample sizes in the same type of experiments are identical as described in the Material and Methods section/Figure legends in detail.

2. Has the statistical analysis been performed appropriately and rigorously?

Reviewer #1: Yes
Reviewer #2: No

All experiments were, as indicated for each experiment in Material and Methods section, performed several times, between 3 and 8 depending on the individual experiment. Not all experiments were repeated the same number of times; however, each tested/examined condition was repeated independently at least 3 times.

To present our results, we chose a form that allows us to show the values obtained in each experiment instead of the more common way of displaying a bar graph with standard deviation. In our chosen form, it is then possible in some cases to see a different number of points that reflect the different number of independent experiments performed for that experiment.

P-values were calculated using unpaired Students' t-tests; * $P < 0.05$ or ** $P < 0.01$ were considered statistically significant. In general, we believe that we performed the statistical analysis appropriately. However, we apologize for not being completely rigorous and not including statistical analysis for each result in the previous manuscript. We tried to address this problem in our current revised version.

3. Have the authors made all data underlying the findings in their manuscript fully available?

The PLOS Data policy requires authors to make all data underlying the findings described in their manuscript fully available without restriction, with rare exception (please refer to the Data Availability Statement in the manuscript PDF file). The data should be provided as part of the manuscript or its supporting information, or deposited to a public repository. For example, in addition to summary statistics, the data points behind means, medians and variance measures should be available. If there are restrictions on publicly sharing data—e.g. participant privacy or use of data from a third party—those must be specified.

Reviewer #1: Yes
Reviewer #2: No

With our original submission, we also provided a PDF file entitled "Compilation of original blots" with all original blots presented in the manuscript. It also is available with this revised version. With the current version, we also provide a document with the data used for the graphs.

4. Is the manuscript presented in an intelligible fashion and written in standard English?

PLOS ONE does not copy edit accepted manuscripts, so the language in submitted articles must be clear, correct, and unambiguous. Any typographical or grammatical errors should be corrected at revision, so please note any specific errors here.

Reviewer #1: Yes

Reviewer #2: Yes

5. Review Comments to the Author

Reviewer #1: *In this manuscript, Camougrand et al examine the regulation of the yeast selective autophagy receptor, Atg32. The authors observe that while Atg32 is specifically expressed during growth in non-fermentable carbon sources, the protein gets degraded in stationary phase, during nitrogen starvation, or rapamycin treatment. The latter two are according to previous observations (Levchenko et al, Plos one, 2016). Surprisingly, and in contrast to this published work, the authors determine that protein destabilization is mediated by the proteasome and not by vacuolar degradation. Using a purification/mass spec approach, they identify a potential ubiquitination site on Atg32. Finally, the authors mutagenize the site, finding a subtle stabilization of the protein and, correlatively, a subtle (but statistically insignificant) enhancement in mitophagy. This study is of potential interest because it suggests a balance between Atg32 function and its degradation by the proteasome may fine-regulate mitophagy, which indeed warrants further exploration. However, the results are in direct conflict with published observations, which is not discussed and should be addressed.*

The majority of the data we obtained do not conflict with the data in the literature. For example, the decrease in the level of Atg32 protein in the stationary phase was observed, among others, by the teams of Dr. Ohsumi and Dr. Levchenko. We are familiar with Dr. Levchenko *et al.*'s (2016) results published in PlosOne (2016).

We have been working on the study of mitophagy for many years and were the first to show the selectivity of the process in 2004 (Kissova *et al.*, JBC) and 2007 (Kissova *et al.*, Autophagy). More importantly, we described the condition in which mitophagy can be observed, and this finding has since served as an essential tool for all research in this area. Namely, to be able to observe mitophagy, yeast cells have to be grown in strict respiratory conditions that allow cells to develop fully differentiated mitochondria and the metabolisms that depend on mitochondria. There is a huge difference in mitochondrial metabolism between the cells grown in media supplemented with a respiratory carbon source (e.g., lactate) or with a fermentative carbon source (e.g., glucose).

In his work, Levchenko *et al.* (2016) used lactate, a respiratory carbon source, which is identical we use. But there is one essential difference between their and our experimental setup: while we shift cells first cultivated in a medium with lactate to nitrogen starvation medium with lactate, for the mitophagy induction, Levchenko et al. used a nitrogen starvation medium supplemented with glucose. In our setup, cells starved for nitrogen in the presence of a respiratory carbon source, while in Levchenko et al.'s setup, the cells' metabolisms are shifted to a different condition. With a shift from

lactate to glucose, not only are mitochondria not needed to the extent they are in respiratory condition, but the quantity of mitochondria is also regulated by the process of mitochondrial biogenesis. In accordance with some published results (e.g., Kanki *at al*, 2009), our unpublished results also showed that in normal growth conditions (in absence of starvation) a simple shift of cells from respiratory condition (lactate) to fermentative condition (glucose) does not induce mitophagy, although the quantity of mitochondria is drastically reduced following such shift in a very short time.

Based on our 40 years of experience with yeast mitochondrial metabolism, it is very difficult to compare the results obtained in two such diverse experimental setups (Levchenko *et al.*'s versus ours). It is easily imaginable that cells have or developed various mechanisms for regulation stability and quantity of Atg32 in these different conditions. One can be more dependent on vacuolar degradation while another can undergo proteasomal degradation control.

Moreover, we did not find data on whether Levchenko's nitrogen starvation medium was buffered to pH5.5, which we do, and it is important.

Additionally, the effects with proteasomal inhibition are only convincingly observed after extended periods of time, raising the concern that the stabilization of Atg32 seen is indirectly due to a stress response rather than acute inactivation of Atg32 degradation.

In our opinion, our results demonstrated a convincing and unambiguous effect of proteasomal inhibition on the stabilization of Atg32 protein in normal growth conditions. Let us explain our statement/results in more detail.

In our study, we used the proteasomal inhibitor, MG-132, in two different types of experiments:

First, we examined levels of Atg32 protein in course of the cell growth. We noticed the apparent decrease in Atg32 levels when cells are entering into a stationary phase of growth—at the time when mitophagy is induced (Fig. 2). In our experiments, we use control cells (T0) harvested in the mid-exponential growth phase that represents the optical density around 1.5–2 OD600. In order to further elucidate the role of Atg32 in mitophagy and regulation of *ATG32* expression, we treated the cells with the proteasomal inhibitor. MG-132 was added into cell culture at T 8 h (the late-exponential phase). Cells grew further in inhibitor presence for additional 16 h (early stationary phase, 24 h) or 40 h (late stationary phase; 48 h). Further, we checked the inhibitor's effect on the viability of cells (Fig. S5A) to ensure cells are not affected in our experimental conditions, because longer incubation time could cause additional stress for cells as the reviewer pointed out. Also, we used time periods of 24 h and 48 h because those are commonly used to examine mitophagy during normal cell growth/stationary phase. Although mitophagy can be induced slightly before cells enter into the stationary phase of growth, its levels are very low before the 24-hour point and cannot be reproducibly detected by immunoblotting using a standard mitophagy test following the Idp–GFP processing tool that is based on releasing a free GFP form. In our work, during normal growth conditions, we did not present results from shorter than 24 hours in the presence of inhibitors, so we are not sure what led the reviewer to raise a concern that stabilization of Atg32 occurs only after long incubation times and may be an indirect effect. The graph in Figure 3B includes T8 for control conditions, but it does not show point T8 plus MG-132 because the inhibitor was only added into the medium at that moment. At the 24 h (as well 48 h) time point, the effect of proteasome inhibition on Atg32 stability is unequivocal, as you can see in Figure 3AB.

Second, after we observed the effect of proteasome inhibition on the Atg32 levels in cells in the

stationary phase, we assessed the alteration of Atg32 protein levels in wild-type yeast cells that were grown in control media and later treated with cycloheximide to turn off protein expression. Cycloheximide was added in both the presence and the absence of MG-132, and the positive effects on stability of Atg32 protein were already observed after a short time of MG-132 treatment—after 20, 40, or 60 min - Fig. 4B,C.

We think that these results reliably dispel the reviewer's concern that the stabilization of Atg32 seen is indirectly due to a stress response rather than acute inactivation of Atg32 degradation.

On the other hand, it is important to note that we used two conditions to induce mitophagy: stationary phase and nitrogen deficiency. We no longer want to use rapamycin because side effects on various cell functions are significant, and certainly rapamycin treatment can represent stress for cells.

Further, the data suggesting that Atg32 is ubiquitinated in a regulated manner at the K282 site are based on an unconvincing purification approach.

Could the reviewer kindly specify his reservation regarding his opinion "an unconvincing purification approach? We believe we follow a standard protocol and purification as described in detail in the Material and Methods section. At the end of purification, all fractions absorbing at 254 nm were pooled and analyzed by electrophoresis on 11% SDS-PAGE and submitted to blue colloidal staining (Fig. 7A: marked as Eluted fraction) and immunoanalysis using antibodies against HIS tag (to detect Atg32 protein) and antibodies against ubiquitin (Fig. 7B). We would like to note we modified Figure 7B to make this clearer, more details were also added into figure legend. Into our current revised version, we also included supplemental Fig. S7 that describes purification and detection of Atg32-V5 more in detail.

We agree that the shift in mobility of Atg32 protein after purification is surprising but not impossible to understand. Atg32 is a protein inserted into the outer membrane of the mitochondria. It has already been observed and published that some mitochondrial membrane proteins, such as e.g. the ATP synthase subunit 9 is mainly found in the form of oligomers in SDS-PAGE gel, despite the presence of detergents in the gel. (FYI: (ref 1) Organization of the yeast ATP synthase F0: a study based on cysteine mutants, thiol modification and cross-linking reagents Jean Velours, Patrick Paumard Vincent Soubannier Christelle Spannagel Jacques Vaillier Geneviève Arselin Pierre-Vincent Graves Biochimica and Biophysica Acta 1458 (2000) 443-456 or (ref 2) ATP Synthase of Yeast Mitochondria Isolation of the subunit h and disruption of the ATP14 gene * Geneviève Arselin, Jacques Vaillier, Pierre-Vincent Graves and Jean Velours ‡ JBC vol. 271, No. 34, Issue of August 23, pp. 20284– 20290 1996). The second reference would provide better insight into the problem.

Regarding Levchenko *et al* article, we understand that it underwent the reviewing process and is now accepted as a "base" for new findings, but the results presented in their article raises several questions for us, such as these:

(i) In Figure 2, a band appears around 100 kDa in the delta pep4 strain after treatment with rapamycin. In Figure 3B, in the delta pep4 delta atgx double mutants treated with rapamycin (time is not indicated), the band is at 100 kDa plus another band which appears above whose size is not indicated.

(ii) In Figure 3C, a band is indicated by an arrow, but the size is not mentioned.

(iii) In Figure 4A and 4C, after treatment with rapamycin, there is a band at 100 kDa + 1 band between 75 and 100 kDa. It should also be noted that the band revealing Atg32 is wide and diffuse. Could these additional bands not correspond to protein aggregates containing Atg32 as revealed by western blots and which would form more in the delta pep4 strain because they are deficient in vacuolar proteases? Why would it not be due to the stress of "rapamycin + absence of vacuolar proteases?"

To conclude, the data from Levchenko *et al.* are disturbing: depending on the gels, different bands are revealed: a band between 75 and 100 kDa, one band at 100 kDa and, in some cases, a band of high molecular weight greater than 100 kDa

In addition, it has recently been shown that other Atg proteins are also ubiquitinated; this is the case for Atg9 and Atg14 (Hu et al, 2020, BBRC). The Atg32 protein would be added to this list.

Specific points:

1. Stabilization of Atg32 upon proteasome inhibition is only observed after 24h of treatment in stationary phase growth. The data shown in Fig. 4B after shorter periods of treatment are not quantified or convincing. This raises the concern that the authors are not observing a prevention of the typical Atg32 degradation, but rather a potential cell stress response or adaptation. Under nitrogen starvation conditions, the stabilization the authors claim exists at 3h is unconvincing, and there is no effect during longer term treatment.

The experiments presented in Figure 3A and Figure 4B do not give the same information.

Figure 4B gives us information on the half-life of the Atg32 protein in the exponential phase. The addition of cycloheximide makes it possible to block the synthesis and to see how long the protein is stable. The results allow us to say that its half-life is shorter than that of a cytosolic (Pgk1) and mitochondrial (porin) protein. Degradation by the proteasome is involved in Atg32 turnover.

In Figure 3A, MG-132 is added after the 8 h time point (late exponential phase) when cell growth slows down to enter the stationary phase. The addition of MG-132 at the early- or mid-exponential (T0) phase of growth is not possible because there would be too much stress for the cells to sustain inhibition of the proteasome along with the growth.

We re-arranged the order of figures in revised manuscript to make this point clearer.

The authors also examine Atg32 stability during MG132/rapamycin treatment (Fig. S3), and this time find a compelling effect. However, this is several hours after treatment and also in conflict with data from Levchenko et al (who treated with MG132 for 2h and saw no stabilization of Atg32). This needs to be discussed/addressed by the authors.

The only explanation we can offer is that a difference in strains and growing conditions leads to the involvement of different pathways regulating stability/expression of Atg32. Based on our experiences, that would not be so surprising. In addition, we used longer rapamycin treatment times.

However, in the study by Levchenko *et al*., the authors do not mention that they observe the same level of Atg32 protein in the wild-type and *pep4Δ* strains (see Fig. 3 and Fig. 4 in their study). We have obtained the same result in our work. Moreover, in the figure legends of their paper, they do not mention in which medium the cells grew.

2. The data identifying a ubiquitinated form of Atg32 are lacking key controls. The authors purify Atg32, and perform a western blot with ubiquitin. However, there is no western blot indicating that the Coomassie stained band is indeed the purified protein. No input is shown from the purification. This is all particularly concerning given that the protein is supposedly SDS resistant after purification, and no protein is observed at the native size.

We have modified Figure 7 to show that the bands revealed by the colloidal blue staining were also detected by anti-HIS and anti-Ub antibodies. Both bands, B1 and B2, were cut and analyzed by mass spectrometry. The Atg32 protein was found in band B1; band B2 corresponds to a histidine-rich protein, which was also found ubiquitinated.

For more details, please, see also our answer for a similar question of the first reviewer above.

The authors do identify one single peptide of ubiquitinated protein, which they map to K282, however, it is impossible to assess if this is biologically relevant as the mutagenized protein has a negligible effect.

The Atg32 protein has 43 lysines, but the analysis covered only 17 lysines; 26 lysines remain for study. We added more details in the manuscript.

3. It is not clear whether quantification of all western blots is normalized to Pgk1, however this is required. The authors state in the legends, "Atg32-V5 expression was quantified as the percentage of Atg32-V5 level of T0".

Thank you for the comment. We modified all the graphs following the amount of Atg32-V5 protein (namely figures 2B, 2D, 3B, 3D, 4C, 5B, 8C, S2B, S3B and S4C) - quantification of all western blots is now normalized to Pgk1.

4. The clarity of the manuscript needs to be improved. The authors should explicitly state growth conditions of their assays. It is not obviously stated that "growth" means allowing cultures to reach saturation after dilution to exponential phase.

Thank you for the comment. We have revised the Material and Methods section, added more information to improve the clarity.

Specific growth media are not always mentioned.

The growth conditions are indicated in each legend of the figures and are always the same - complete minimal synthetic medium with lactate as carbon source (CMS-L; described in the Material and Methods section in detail), except for Figures 5A and B, where galactose was used as a carbon source (CMS-G) because the mutant *pre2-2* does not grow in media supplemented with respiratory carbon source - lactate. This is also explained in the Figure legends as well as in the Results part.

Another example of confusion is the Idp1 degradation assay – while this may be standard for the field, the relationship between its cleavage as an indicator of mitophagy is not explicitly stated.

Thank you for the comment. It is a test commonly used in all studies focusing on mitophagy in yeast. We modified the text and described the method following the processing of the Idp-GFP protein as an indicator of mitophagy induction, the reference and description are also provided in the Material and Methods section.

Reviewer #2: *In this manuscript, the authors found that the mitophagy receptor Atg32 is degraded upon mitophagy induction or under nitrogen starvation conditions. They further showed that the degradation of Atg32 is mediated by the 26S proteasome but not by autophagy. Using mass spectrometry analysis, they identified Lys 282 is an ubiquitination site on Atg32. Intriguingly, the replacement of Lys 282 with Ala did not slow down the degradation rate of Atg32 in their experimental conditions. Overall, the authors concluded that the yeast mitophgy receptor Atg32 can be ubiquitinated and degraded by the proteasome.*

Based on the presented data, this reviewer think this manuscript is not technically sound and many critical controls are missing (see below for details). The quantification data are confused, as some blots contain 5 repeats whereas some blots in the same experiments contain 6 repeats (for example, 8 hrs vs. 24 hrs in Fig. 2B and Fig. 3B). Most quantification assays lack statistical analyses. The writing basically sounds good, but typos and errors avoid the reviewer to fully understand the context.

Indeed, we accumulated more data at 24 h and 48 h of growth because these were the most important conditions for our study. The 8 h point was not always taken, which explains the difference in points. We have chosen this presentation for the graphs to show all the data obtained. This question could have been avoided if we had presented the results in the form of bars.

Major problems:

1. The Atg32 degradation assay were performed by using an epitopic-tagged Atg32 construct instead of integrated tagged Atg32, which makes the full story less convincing. This reviewer suggest the authors at least re-examine the stability and ubiquitination of Atg32 using integrated form.

As we already mentioned above, to our knowledge, no specific antibodies against the Atg32 protein are available so far. We agree that labeling the protein at the locus would have been the ideal construct for every study aiming to examine stability and expression of the Atg32 protein. However, we believe that the construct we used in our study to express Atg32 protein with a V5-6HIS label at its C-terminus is a good compromise. We assert that the *ATG32* gene in our construct has been put under the control of its own promoter. This type of strategy has been used in many published works.

2. In Figure 1A, the authors should examine the localization of Atg32-V5 protein by immunoflorescent staining instead of gradient assay alone. Co-fraction of two protein does not necessarily mean that they spatially localized together. Moreover, in previous results, Dr. Koji Okamoto showed that the C-terminal tagging in Atg32 would disrupt mitochondrial targeting and out-membrane insertion.

Therefore, they generated an internally 3xHA-tagged Atg32 variant (Atg32-3HAn) to replace the endogenous protein. This is the major problem in this manuscript.

We have chosen a biochemical approach to look at the localization of the Atg32 protein, which is as relevant as fluorescence experiments. Using gradients to fractionate cell extracts has long been common. The results show that the Atg32 protein is found in the same fraction as the porin that is localized to the mitochondrial outer membrane when study was performed on total cell lysate (Fig. 1A). In new supplementary figure 1A, we also isolated mitochondria that we loaded on a sucrose gradient - Atg32-V5 protein was found in the same fractions as porin.

3. CHX assay is critical to confirm the decrease of Atg32 over the time (upon to 48 hrs) under mitophagy induction is due to protein degradation but not the synthesis of mRNA transcripts. Unfortunately, the whole Figure 4 are somehow disrupted and this reviewer can not see it.

Perhaps we did not fully clarify our rationale for the experiment with cycloheximide which was to check the half-life of the Atg32 protein. We modified the text in the manuscript to explain the purpose of this experiment better.

4. In Figure 3, the ubiquitination levels of Atg32 should be examined by immunoprecipitation of Atg32. The ubiquitination levels of total proteins does not mean Atg32 is also ubiquitinated.

We agree with this comment. However, the purpose of the ubiquitin blot was to control and show the effect of MG-132 as an effective inhibitor of proteasome in our experimental setup. We added an explanation in the manuscript.

In addition, PMSF is unstable in aqueous solutions, and its half-life is about 30 min. How the authors exclude the possibility that less protein levels of Atg32 is due to no effect of PMSF after 24 or 48 hours incubation.

We agree with the comment. We added PMSF several times during the experiment, and we added this information in the manuscript.

In addition, we used the *pep4Δ* mutant lacking vacuolar proteases and obtained the same results as we obtained with the use of a vacuolar protease inhibitor (PMSF).

5. In Figure 5A, the strain source of pre2-2 should be referred. The authors should clearly describe how they inactivate 20S proteasome using this mutant strain.

Dr. Sagot kindly contributed the *pre2-2* mutant. We added this information in the text. Pre2 protein is the β5 subunit of the 20S proteasome and is responsible for the chymotryptic activity of the proteasome. We added this information in the text.

The positive control is missing in 5A and 5C.

For us, the positive controls were the two strains, atg32Δ + Atg32-V5 and BY4742+ Atg32-V5.

6. In Figure 6D, ATG32 deletion strain should be included as a negative control.

Like processing of the Idp-GFP tool for detection of mitophay by western blot technique, the mt-PHO8 ALP reporter test is usually used as a biochemical approach to study mitophagy in yeast. To our knowledge, including the *atg32Δ* mutant strain (defective for mitophagy) as a negative control is not always required.

However, we included for the reviewer a figure to show that mitophagy (mtALP activity) is not induced/elevated over basal level *(T0 wild type - 1st column; T0 mutant- 3rd column)* during starvation in *atg32Δ* mutant *(-N6h mutant - 4th column)* as compared to wild type where mtALP increased about 3 times at the same time *(-N6h wild type - 2nd column).* We performed this experiment under the same conditions as described in Fig. 6D.

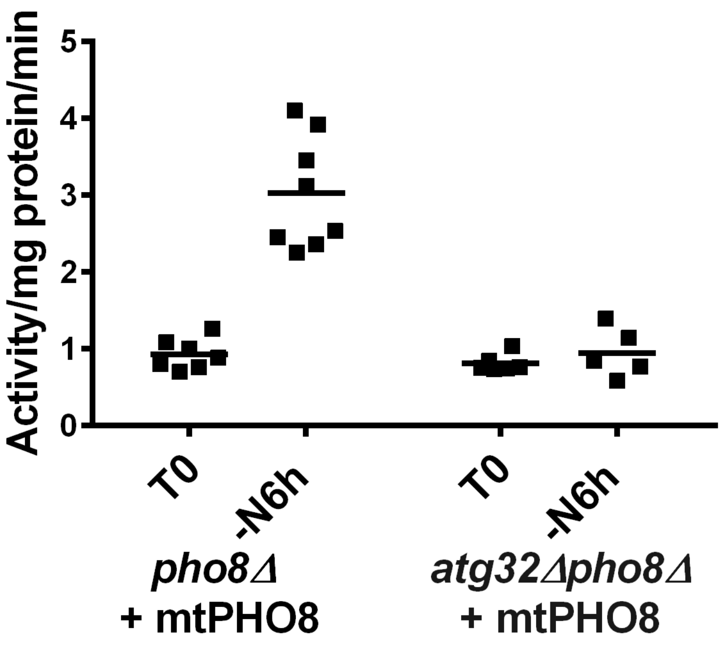

7. From Figure 7 to 10, these data may remove to the supplemental data since they are negative results and the model figure is not closely related to the main conclusion.

While we understand your concern, even if they are negative results, we would like to keep them among the main results, in accordance with PLOS policy.

8. The discussion part should be more concise.

We modified the Discussion part of the manuscript at places where it seemed to help the clarity of the manuscript. To improve the clarity of the manuscript, we modified Results, Material and Methods, figures substantially.

Minor issues: were corrected as needed

1. Page 8, line 170, the font of “107” is weird.
2. Page 11, line 221, remove “we”
3. Page 14, line 291, the blue label should be removed.
4. Page 31, line 709, where shows “against histidine”?
5. Figure 7B, “imput” should be “input”.
