# Supplementary material for: The yeast mitophagy receptor Atg32 is ubiquitinated and degraded by the proteasome
Source: PLoS One. 2020 Dec 23;15(12):e0241576. doi: 10.1371/journal.pone.0241576 (PMC7757876; doi:10.1371/journal.pone.0241576)
Supplement: Attachment — Submitted filename: Answers to reviewer final.pdf [file pone.0241576.s014.pdf]

**Answers to reviewer 1**

*Reviewer #1: In the revised manuscript by Camougrand et al, the authors made modest changes to address concerns raised in their original submission. While I have my doubts that their purification strategy is effectively working (ie, a concern is that the oligomerized band is non-specifically cross-reacting with the anti-his antibody), the authors have now provided the requested data.*

*However, it is somewhat glaring that the authors have now removed data from Figure 8 related to the ubiquitinated band they identified via proteomic analysis. They had previously generated two clonal lines of a K282A mutant, one of which showed no change in Atg32 turnover and the other which mildly stabilized Atg32 at the 8h timepoint. In the revised manuscript, the "negative" data is removed while "significantly (P<0.05) impaired" is added to the text. The authors should return "clone 1" to the manuscript for transparency purposes and graphically display the values that led to this conclusion.*

We would like to thank you for all your effort and comments. We believe they helped us to make our manuscript better.

We apologize if the noninclusion in our revised manuscript of the immunoblot result with clone 1 in Figure 4A gave the impression that we are trying to intentionally select the results that fit into our story. By no means was this our intent. We only tried to simplify the presentation of the results and eliminate unnecessary duplication. Let us provide you with a more detailed explanation.

In the original version, we presented in Figure 8A an image of blots for two different clones (1 and 2). In Figure 8C, there is only one column for the mutant K282A (it does not specify if it is clone 1 or clone 2). We apologize for not explaining this better in the text—the column (48h) contains results from 6 independent experiments from 3 different clones that we tested (clone 1 and clone 2 from Figure 8A plus a third clone that was not mentioned in the manuscript). Also, it has to be noted that in the original version, the y-axis of the graph in Figure 8C shows the amount of Atg32-V5 normalized to T0 value of Atg32-V5, which, based on the reviewer's request, was normalized in the revised version to the amount of Pgk1 at each time.

Again, Figure 8 in the revised version of our manuscript does include the results of 6 independent experiments from 3 individual clones. Because quantification normalized to Pgk1 did not show significant changes between individual clones (1-2-3), we decided to keep only one of the clones in part A. When compared to wild-type Atg32 protein, there is about 15% ($P<0.05$) more mutant protein in the late stationary phase cells (Fig. 8A, C; 48 h).

For the reviewer, we attach here the immunoblot results obtained from 3 individual clones with individual quantification for each of them for comparison.

**Figure for reviewer only**

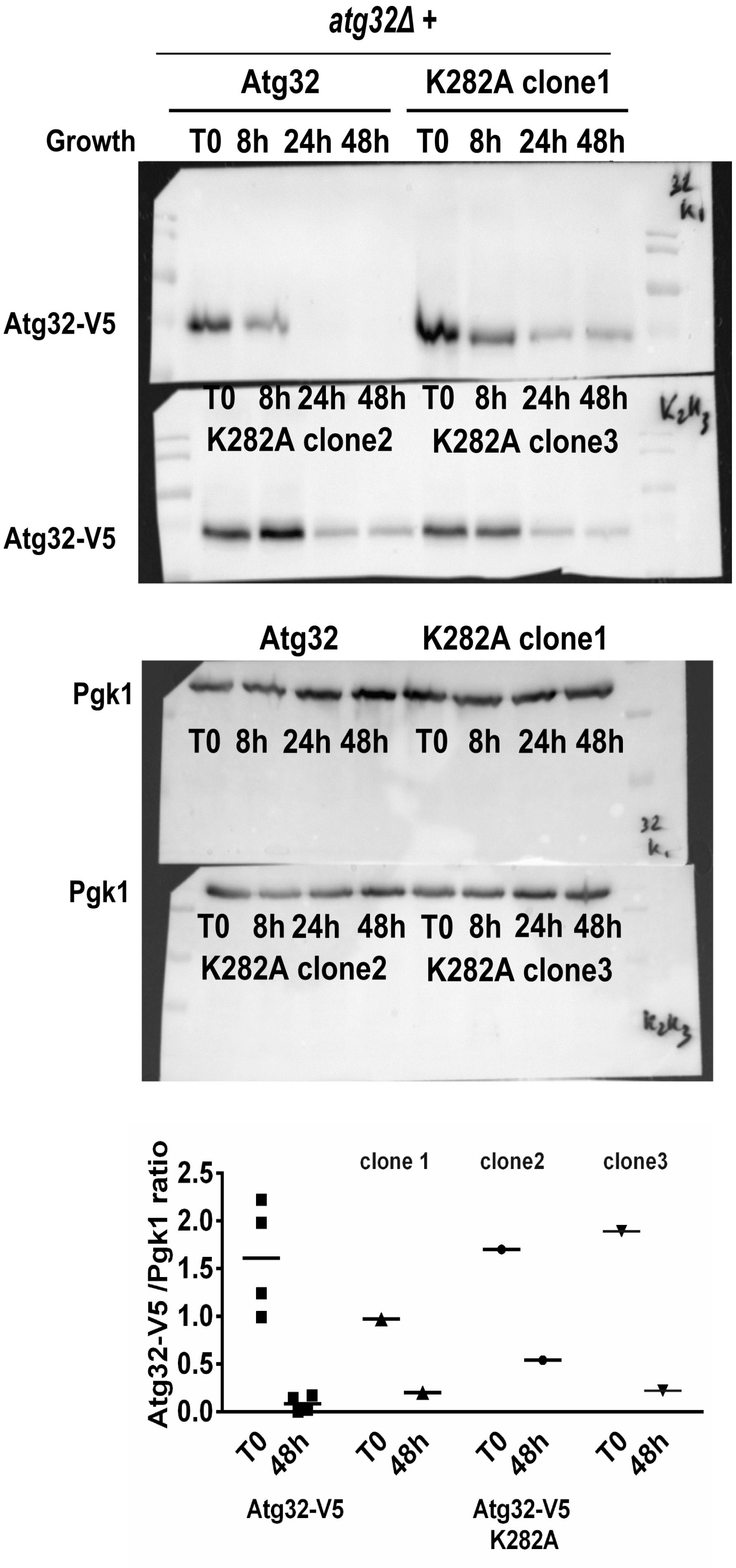

atg32Δ +
Atg32
K282A clone1
Growth
T0 8h 24h 48h T0 8h 24h 48h
Atg32-V5
T0 8h 24h 48h T0 8h 24h 48h
K282A clone2
K282A clone3
Atg32-V5
Atg32
K282A clone1
Pgk1
T0 8h 24h 48h T0 8h 24h 48h
Pgk1
T0 8h 24h 48h T0 8h 24h 48h
K282A clone2
K282A clone3
Atg32-V5 /Pgk1 ratio
2.5
2.0
1.5
1.0
0.5
0.0
clone 1
clone2
clone3
T0
48h
T0
48h
T0
48h
T0
48h
Atg32-V5
Atg32-V5
K282A

**Figure 8 : first soumission**

# Figure 8

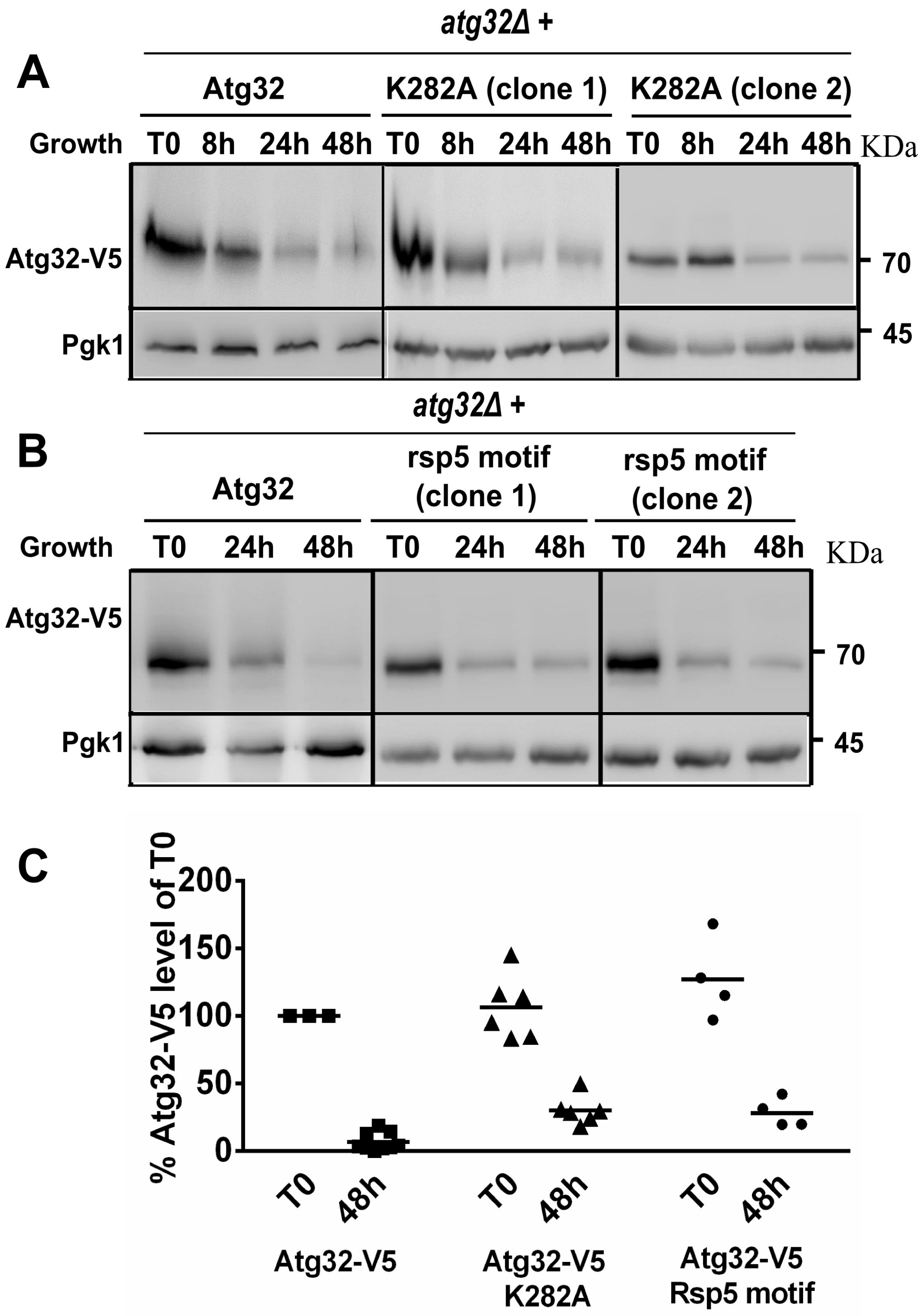

Figure 8 : second revision

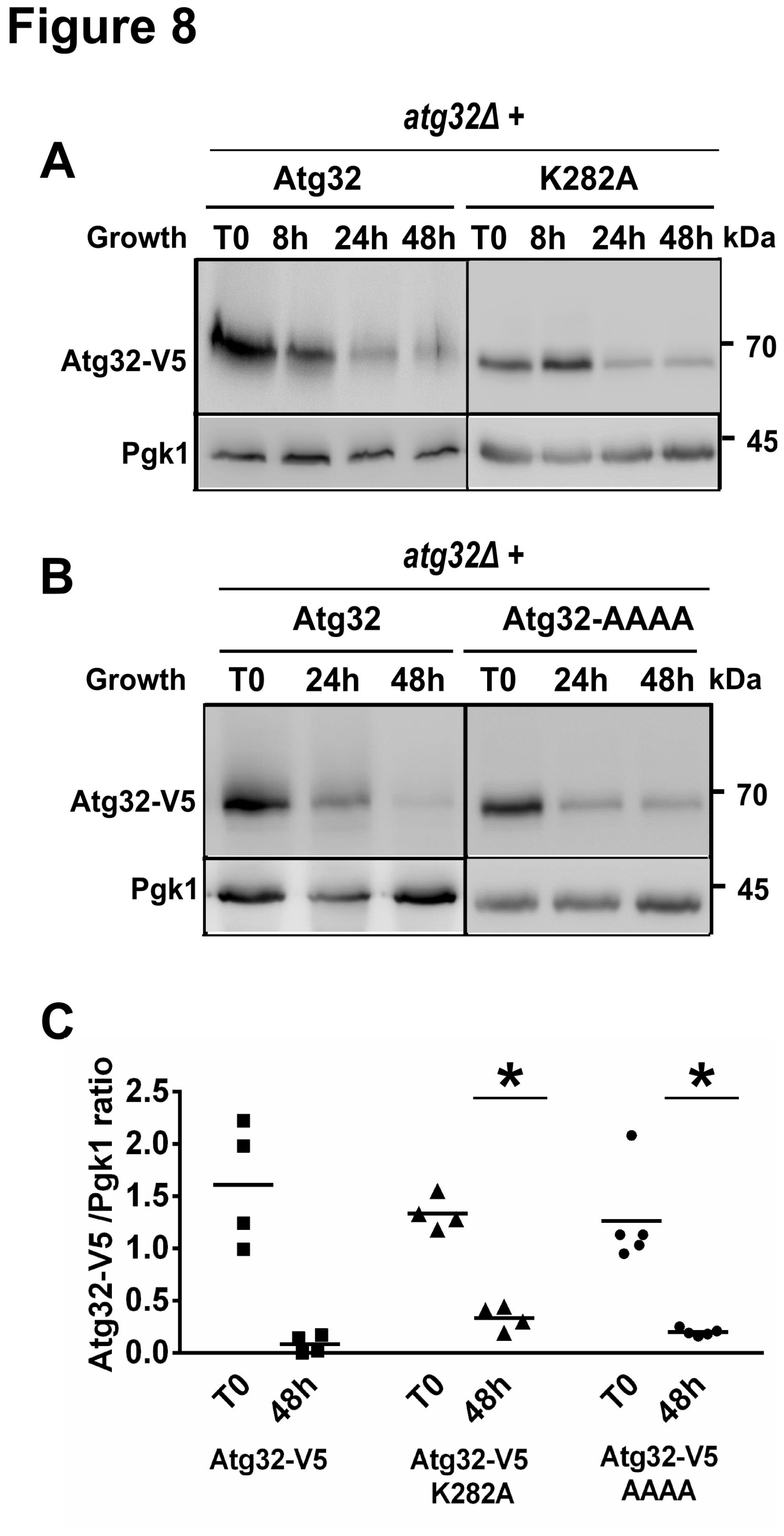

*My other primary concern regarding lengthy treatment with MG-132 was not addressed. In their response, the authors write that "we did not present results from shorter than 24 hours in the presence of inhibitors, so we are not sure what led the reviewer to raise a concern that stabilization of Atg32 occurs only after long incubation times and may be an indirect effect." This is exactly the point – because the authors do not examine other time points (for example, 1h, 2h, 4h) after MG-132 addition at 8h, they can only state that 16h of treatment leads to stabilization of Atg32 during stationary growth. While the authors now demonstrate that such treatment does not inhibit cell growth, they also cannot state whether the protein is acutely stabilized or stabilized as part of an adaptive cellular response to prolonged proteasomal inhibition.*

In our previous answer we tried to explain to the reviewer that we did not examine the shorter times (fewer than 16 hours; T24) of treatment with MG-132 because our aim was to study levels of Atg32 protein at the beginning of and during the stationary phase—the time of cell growth when mitophagy is induced. We believe that our results provide clear evidence that inhibition of proteasome activity with MG-132 leads to stabilization of Atg32 during the stationary phase and that this correlates with an increase in mitophagy activity.

However, to address the reviewer's comment, we performed an experiment in which MG-132 was added to the culture at T0 (instead of at T8 as we used during our study) and examined the levels of Atg32 after 8 hours (exponential phase, no appreciable mitophagy), 24 hours (early stationary phase, beginning of mitophagy induction), and 48 hours (late stationary phase). The obtained results were included in the manuscript as supplementary Fig. 4D (for your convenience the results are attached to this letter as well). As you can see, the level of Atg32p at T8 is about half compared to the level at T0, and it further decreases to where there is almost no detectable Atg32 when measured at T48. On the contrary, the presence of MG-132 drastically recovers Atg32 levels in both short (8h) and long (24h and 48h) incubation times. We provide the quantification of the immunoblot results here as well. We strongly believe these results support the view that the Atg32 protein is acutely stabilized instead of being stabilized as a part of an adaptive cellular response to prolonged proteasomal inhibition.

**New supplementary Figure S4 (with new results in part D - included into manuscript):**

## Figure S4

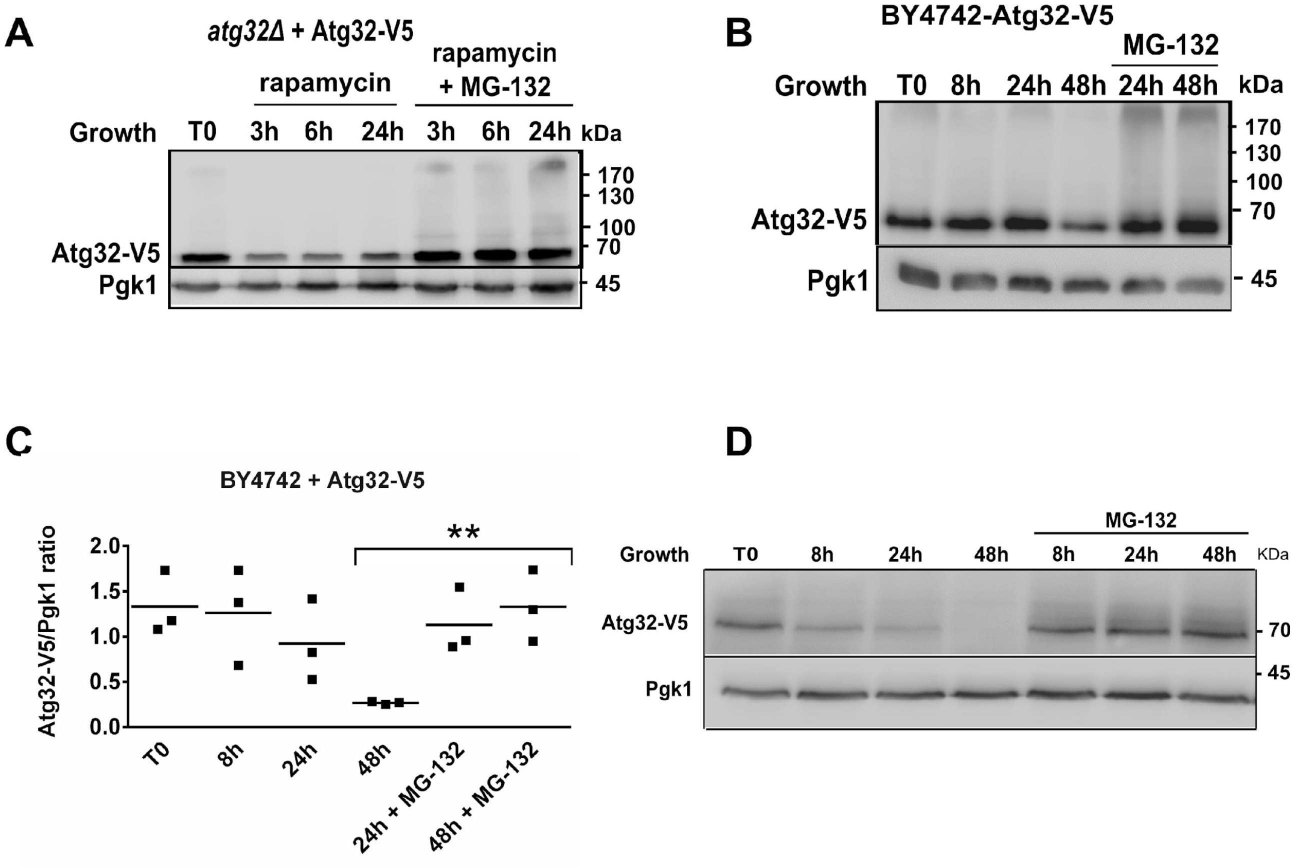


**Quantification figure S4D- an average from 2 independent experiment (for reviewer only):**

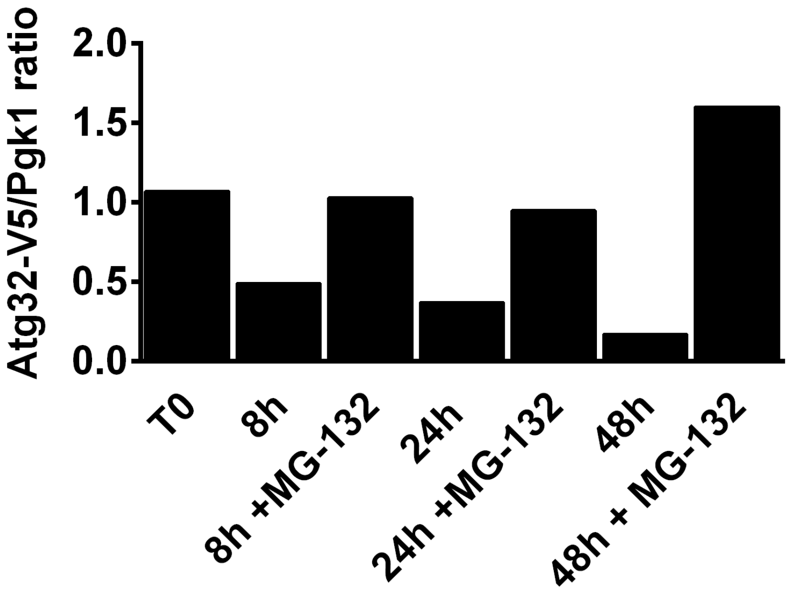

*In exponentially growing cells, where there is no appreciable mitophagy, the authors use short-term MG132 treatment in combination with cycloheximide and observe a very modest inhibition of proteasomal turnover. So while the authors can conclude that Atg32 can be targeted to the proteasome during exponential growth, it is not clear that this is an acute cellular response to regulate mitophagy when cells reach stationary phase. If the authors are unwilling to perform new experiments, they must substantially improve the clarity of the manuscript and acknowledge the potential caveats and alternative explanations of their results.*

Beside our aforementioned explanation, our experiments with cycloheximide (Figs. 4B and 4C) showed that the turnover of the Atg32 protein is extremely rapid compared to other proteins (porin, Pgk1) and that this turnover involves the activity of the proteasome. This also explains why the activity of the promoter increases during growth (Fig. 4A). After one hour with the cycloheximide, the effect of MG-132 is striking and significant (P=0.0146; we included data for “1h+cycloheximide+MG-132” in Fig. 4C that were missing in the previous version). Respectfully, we cannot agree with the reviewer’s statement that this change is “very modest.”

Our results support the conclusion that Atg32 can be targeted to the proteasome during exponential growth as well as favoring an acute cellular response to regulate levels of Atg32 protein (as an essential mitophagy regulator) when cells reach the stationary phase.

We slightly modified some parts of the Results and Discussion sections to make the text clearer.

The manuscript was proofread by a native English-speaking person.
