# Supplementary material for: The yeast mitophagy receptor Atg32 is ubiquitinated and degraded by the proteasome
Source: PLoS One. 2020 Dec 23;15(12):e0241576. doi: 10.1371/journal.pone.0241576 (PMC7757876; doi:10.1371/journal.pone.0241576)
Supplement: Attachment — Submitted filename: PONE-D-20-11526.docx [file pone.0241576.s012.docx]

In this manuscript, the authors found that the mitophagy receptor Atg32 is degraded upon mitophagy induction or under nitrogen starvation conditions. They further showed that the degradation of Atg32 is mediated by the 26S proteasome but not by autophagy. Using mass spectrometry analysis, they identified Lys 282 is an ubiquitination site on Atg32. Intriguingly, the replacement of Lys 282 with Ala did not slow down the degradation rate of Atg32 in their experimental conditions. Overall, the authors concluded that the yeast mitophgy receptor Atg32 can be ubiquitinated and degraded by the proteasome.

Based on the presented data, this reviewer think this manuscript is not technically sound and many critical controls are missing (see below for details). The quantification data are confused, as some blots contain 5 repeats whereas some blots in the same experiments contain 6 repeats (for example, 8 hrs vs. 24 hrs in Fig. 2B and Fig. 3B). Most quantification assays lack statistical analyses. The writing basically sounds good, but typos and errors avoid the reviewer to fully understand the context.

Major problems:

1. The Atg32 degradation assay were performed by using an epitopic-tagged Atg32 construct instead of integrated tagged Atg32, which makes the full story less convincing. This reviewer suggest the authors at least re-examine the stability and ubiquitination of Atg32 using integrated form.
2. In Figure 1A, the authors should examine the localization of Atg32-V5 protein by immunoflorescent staining instead of gradient assay alone. Co-fraction of two protein does not necessarily mean that they spatially localized together. Moreover, in previous results, Dr. Koji Okamoto showed that the C-terminal tagging in Atg32 would disrupt mitochondrial targeting and out-membrane insertion. Therefore, they generated an internally 3xHA-tagged Atg32 variant (Atg32-3HAn) to replace the endogenous protein. This is the major problem in this manuscript.
3. CHX assay is critical to confirm the decrease of Atg32 over the time (upon to 48 hrs) under mitophagy induction is due to protein degradation but not the synthesis of mRNA transcripts. Unfortunately, the whole Figure 4 are somehow disrupted and this reviewer can not see it.
4. In Figure 3, the ubiquitination levels of Atg32 should be examined by immunoprecipitation of Atg32. The ubiquitination levels of total proteins does not mean Atg32 is also ubiquitinated. In addition, PMSF is unstable in aqueous solutions, and its half-life is about 30 min. How the authors exclude the possibility that less protein levels of Atg32 is due to no effect of PMSF after 24 or 48 hours incubation.
5. In Figure 5A, the strain source of *pre2-2* should be referred. The authors should clearly describe how they inactivate 20S proteasome using this mutant strain. The positive control is missing in 5A and 5C.
6. In Figure 6D, *ATG32* deletion strain should be included as a negative control.
7. From Figure 7 to 10, these data may remove to the supplemental data since they are negative results and the model figure is not closely related to the main conclusion.
8. The discussion part should be more concise.

Minor issues:

1. Page 8, line 170, the font of “10^7^” is weird.
2. Page 11, line 221, remove “we”
3. Page 14, line 291, the blue label should be removed.
4. Page 31, line 709, where shows “against histidine”?
5. Figure 7B, “imput” should be “input”.
